# Expanding Peptide Chemical Space via Acid-Mediated Arginine Modification

**DOI:** 10.1021/acs.orglett.5c03977

**Published:** 2025-10-30

**Authors:** Pinki Sihag, Minyoung Kwon, Ankita Misra, Monika Raj

**Affiliations:** Department of Chemistry, 1371 Emory University, Atlanta, Georgia 30322, United States

## Abstract

We describe an acid-mediated chemoselective method for the targeted modification of arginine residues in peptides. Malonaldehyde efficiently converts guanidinium side chains into amino pyrimidine moieties with near-quantitative conversion across diverse substrates. Side products are reversible with butylamine, underscoring the method’s robustness. The resulting amino pyrimidine peptides exhibit enhanced cellular permeability and allow late-stage diversification into imidazo­[1,2-*a*]­pyrimidinium salts. This strategy expands the chemical space of peptide modification, providing a versatile platform for peptides with improved drug-like properties.

## Graphical abstract

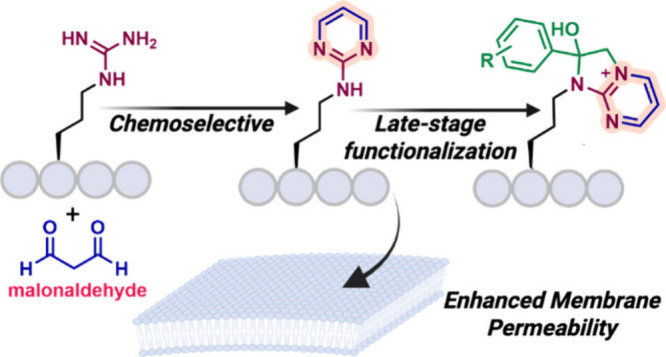

## Introduction

Targeted functionalization of amino acids within peptides is a powerful strategy to expand chemical diversity and tailor biological function, surpassing the constraints of de novo amino acid incorporation.^1^ While most of the existing methods exploit highly reactive amino acid side chains, such as lysine, serine, cysteine, tyrosine, tryptophan, or histidine, arginine remains underexplored despite its prevalence (∼5.8% of residues in proteins) and its biologically essential guanidinium group.^2^ This functional group stabilizes DNA, RNA, and protein interactions, yet its high p*K*_a_ (∼12.5) and low nucleophilicity render selective modification highly challenging.^3^ Current labeling strategies for arginine, largely based on 1,2-dicarbonyls such as phenylglyoxal or 1,2-cyclohexanedione, generate complex mixtures (Scheme 1).^4^ Thus, developing robust chemoselective strategies for arginine modification represents a major opportunity to advance peptide engineering and chemical biology.

**Scheme 1:**
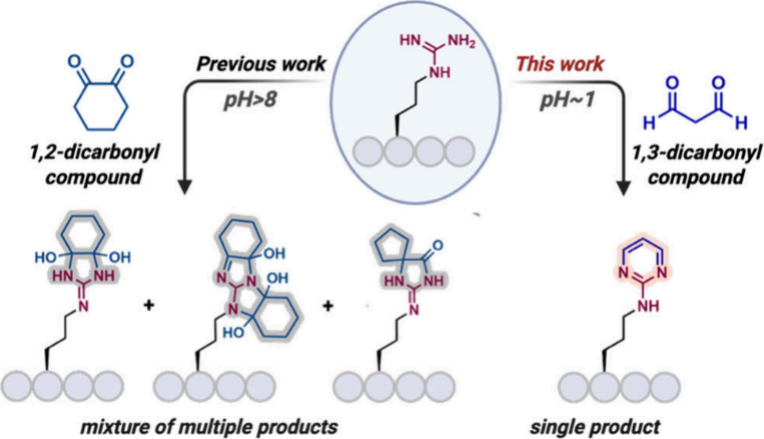
Background and Reaction Development

We recognized that arginine, uniquely enriched with heteroatoms in its side chain, can be harnessed as a gateway to a heteroaromatic motif. By converting guanidinium into stable amino pyrimidine derivatives, we envisioned expanding the functional space of peptides. Amino pyrimidines offer aromatic stability, dual hydrogen-bond donor/acceptor capacity, and broad pharmacological relevance, having been widely employed in drug development as anti-infective, anticancer, and neuroactive scaffolds.^5^ Their integration into peptides could confer enhanced stability, cell permeability, and drug-like properties while retaining the specificity of peptide-based recognition.^6^ This hybridization of peptide and heteroaromatic chemistries promises to overcome key limitations of peptide therapeutics including poor bioavailability and inefficient cellular uptake.

Herein, we report an acid-mediated, chemoselective approach for incorporating amino pyrimidine rings into peptides via selective reaction of 1,3-dicarbonyls such as malonaldehyde with arginine side chains. The transformation efficiently converts arginine residues to amino pyrimidine derivatives with high conversion rates and minimal byproduct formation. Undesired byproducts formed due to the reaction of malonaldehyde with other reactive amino acid residues are effectively reversed by butylamine, highlighting the chemoselectivity of the method. Beyond simple modification, the resulting amino pyrimidines are versatile handles, and we demonstrate the late-stage functionalization (LSF) into diverse imidazo­[1,2-*a*]­pyrimidinium salts using bromoacetophenone derivatives, further expanding structural diversity. Importantly, peptides bearing amino pyrimidine moieties exhibit enhanced stability and permeability, highlighting the translational potential of this chemistry to generate peptide-based drugs with improved pharmacological profiles.

## Results and Discussion

To establish a chemical method for selective arginine labeling, we first examined the reaction of the model peptide Ac-RYF (**1a**) with malonaldehyde (MDA) under various solvent conditions at room temperature (Table 1). Initial trials with 75 equiv of MDA in water, sodium carbonate buffer (pH 10), NaOH (1 M), or citrate buffer (pH 4) showed no detectable formation of any product (entries 1–4). Changing the reaction solvent to 6 M HCl yielded 13% conversion to the amino pyrimidine product **2a** (entry 5), which increased to 23% upon extending the reaction time to 2 h (entry 6). Raising MDA loading to 100 equiv significantly enhanced the conversion to 66% (entry 7). Increasing the HCl molarity to 8 M improved product formation at lower loading of MDA (entries 8–9). Further optimization with strong acid revealed that 12 M HCl provided the highest efficiency, affording 75% conversion with 25 equiv of MDA (entry 10). Increasing the MDA equivalents from 50 to 100 under these conditions resulted in nearly quantitative conversion (>99%) (entries 11–13). Using TFA as a solvent does not result in the formation of the product (entry 14).

**Table 1:**
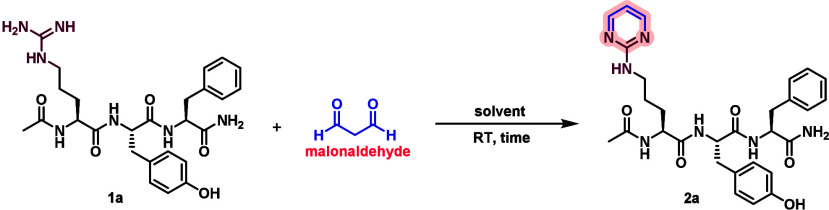
Optimization of the Reaction Conditions^a^

Entry	Solvent	MDA (equiv)	% Conversion^b^
1	H_2_O	75	0
2	Na_2_CO_3_ (pH 10)	75	0
3	1 M NaOH	75	0
4	C_6_H_8_O_7_ (pH 4)	75	0
5	6 M HCl	75	13
6^c^	6 M HCl	75	23
7	6 M HCl	100	66
8	8 M HCl	75	26
9^c^	8 M HCl	75	33
10	12 M HCl	25	75
11	12 M HCl	50	90
12	12 M HCl	75	96
**13** ^d^	**12 M HCl**	**100**	**>99**
14	TFA	100	0

a Unless otherwise noted, all reactions were carried out using **1a** (0.002 mmol, 1.0 equiv), MDA (0.2 mmol, 100 equiv) in 500 μL solvent at room temperature for 1 h.

b Conversion is determined by HPLC at 220 nm.

c Reaction time is 2 h.

d Optimized reaction conditions.

Together, these results establish that reaction of arginine-containing peptides with 100 equiv of MDA in 12 M HCl at room temperature for 1 h provides optimal conditions for quantitative conversion of arginine into amino pyrimidine.

The chemoselectivity of the optimized protocol was evaluated using peptides containing other reactive residues, including Trp (**1b**), Lys (**1c**), and Trp/His (**1d**) (Figure 1). In all cases, MDA modification produced the expected Arg-derived amino pyrimidine along with condensation byproducts from nucleophilic residues (Figure 1). Importantly, these undesired modifications were selectively reversed by butylamine treatment, restoring a single Arg-modified amino pyrimidine product. Unlike traditional arginine-labeling methods with 1,2-dicarbonyls that often yield complex mixtures, this strategy uniquely enables selective reversal of side products and generates a single amino pyrimidine product, underscoring its robustness and adaptability in diverse peptide environments. Notably, in the absence of Arg, a histidine-containing peptide (**1e**) generated only a trace amount of condensation byproduct with MDA, which was efficiently removed by BuNH_2_ treatment, regenerating >96% of the starting peptide (see Supporting Information). It has been also found that cysteine does not show reactivity toward MDA under optimized reaction conditions (see Supporting Information). Use of 1,3-diketone or acetylacetone resulted only in 25% amino pyrimidine product along with a minor amount of C-terminal amide hydrolysis product.

**Figure 1:**
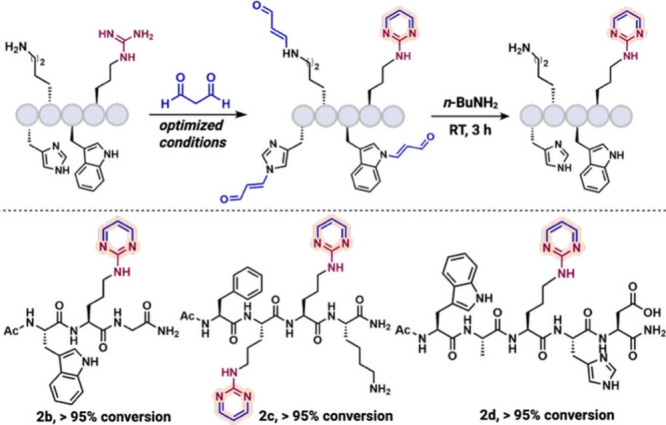
Chemoselectivity study of MDA with peptides containing reactive amino acids including tryptophan, lysine, and histidine along with arginine. Unless otherwise noted, all reactions were carried out using **1b**–**1d** (0.002 mmol, 1.0 equiv) and MDA (0.2 mmol, 100 equiv) in 500 μL of 12 M HCl at room temperature for 1 h. Reversal of side products with Trp, Lys, and His is carried out by reacting with BuNH_2_ (75 equiv) at room temperature for 3 h.

The scope of the protocol was extended to various arginine-containing peptides (**1f**–**1k**) (Figure 2). Under the optimized conditions, all substrates were efficiently converted to their amino pyrimidine derivatives (**2f**–**2k**) with >99% conversion. Notably, a peptide containing two arginine residues (**1k**) underwent modification to both amino pyrimidines (**2k**) quantitatively (>99%). To evaluate the impact of this modification on cellular uptake, we performed cell-based permeability assays (CAPA) on two amino pyrimidine chloroalkane-tagged peptides (**ct-2l** and **ct-2m**) and their unmodified controls (**ct-1l** and **ct-1m**) (Figure 3).^7^ The results demonstrated a 2-fold increase in membrane permeability following conversion of arginine into the amino pyrimidine derivative. This enhanced uptake can be attributed to redistribution of positive charge within the amino pyrimidine ring combined with increased hydrophobic character, which together facilitated more efficient transport across cell membranes.

**Figure 2:**
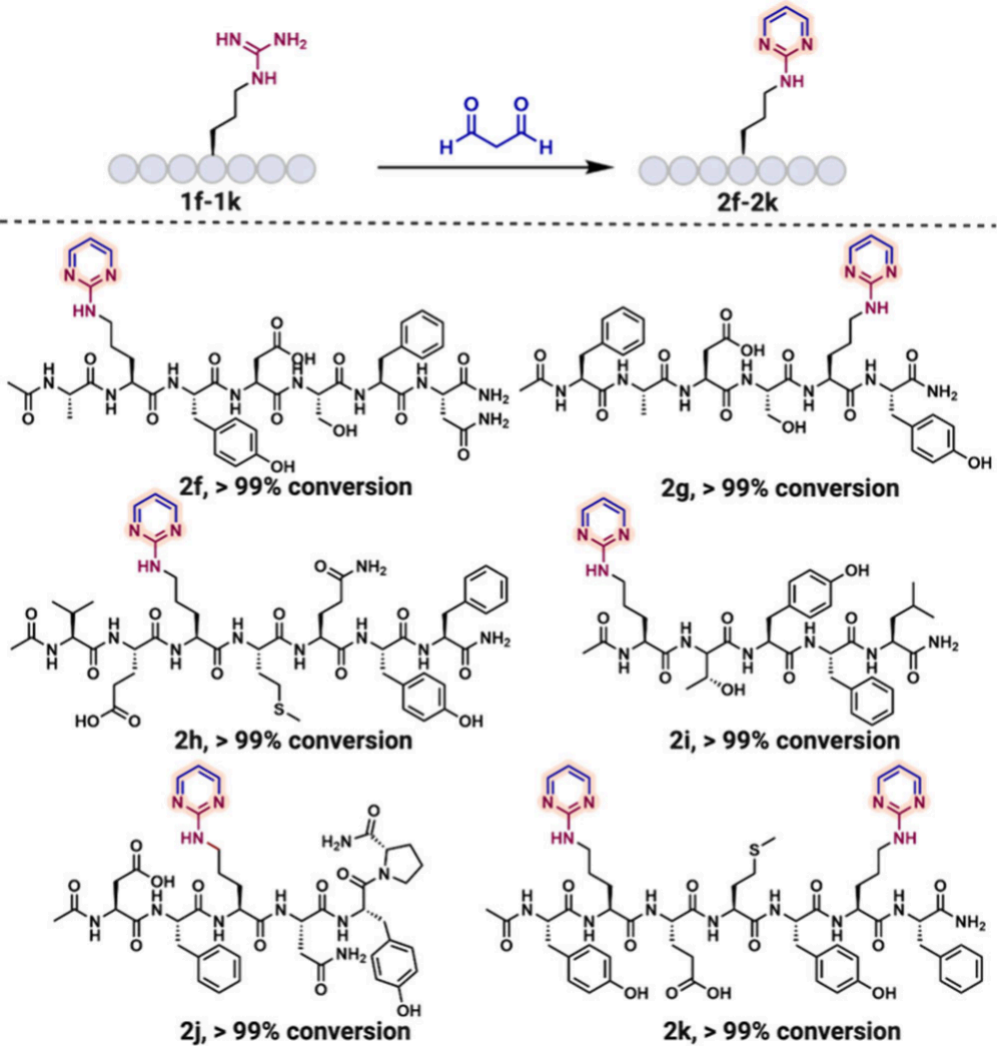
Substrate scope of arginine-containing peptides **1f**–**1k** to amino pyrimidine peptides **2f**–**2k**. Unless otherwise noted, all reactions were carried out using **1f**–**1k** (0.002 mmol, 1.0 equiv) and MDA (0.2 mmol, 100 equiv) in 500 μL of 12 M HCl at room temperature for 1 h.

**Figure 3:**
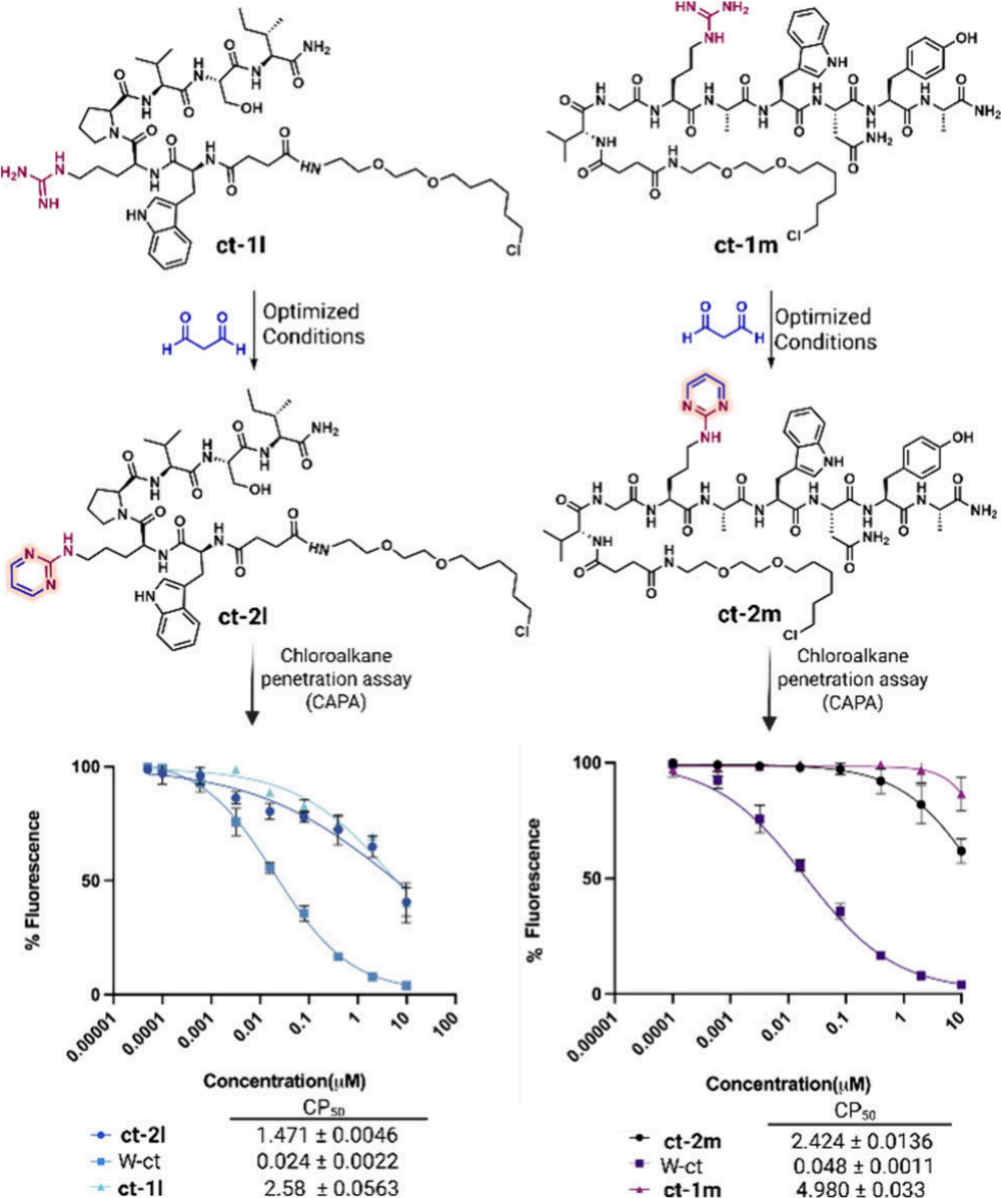
CAPA assay of unmodified (**ct-1l** and **ct-1m**) and modified (**ct-2l** and **ct-2m**) peptides to determine their cell permeability. Unless otherwise noted, all reactions were carried out using **1l** or **1m** (0.002 mmol, 1.0 equiv) and MDA (0.2 mmol, 100 equiv) in 500 μL of 12 M HCl at room temperature for 1 h. W-ct = “Wild-type chloroalkane” control (in this case TAMRA with chloroalkane tag).

Late-stage functionalization of the amino pyrimidine **2n** obtained from the arginine derivative was achieved by reaction with various substituted 2-bromoacetophenone derivatives **3a**–**3d** in the presence of catalytic base (Figure 4).^8^ This transformation furnished a series of fused heteroaromatic systems, imidazo­[1,2-*a*]­pyrimidinium salts (**4a**–**4d**), in good conversions (63–75%, Figure 4). These salts represent versatile intermediates, offering opportunities for structural diversification and seamless incorporation into peptide frameworks. This strategy extends beyond simple arginine modification, enabling the creation of heteroaromatic and fused heteroaromatic scaffolds with broad potential for drug discovery and peptide engineering. The chemoselectivity of 2-bromoacetophenone has been extensively characterized in the literature and is known to be highly biased toward cysteine without modifying any other amino acids in the proteins.^9^

**Figure 4:**
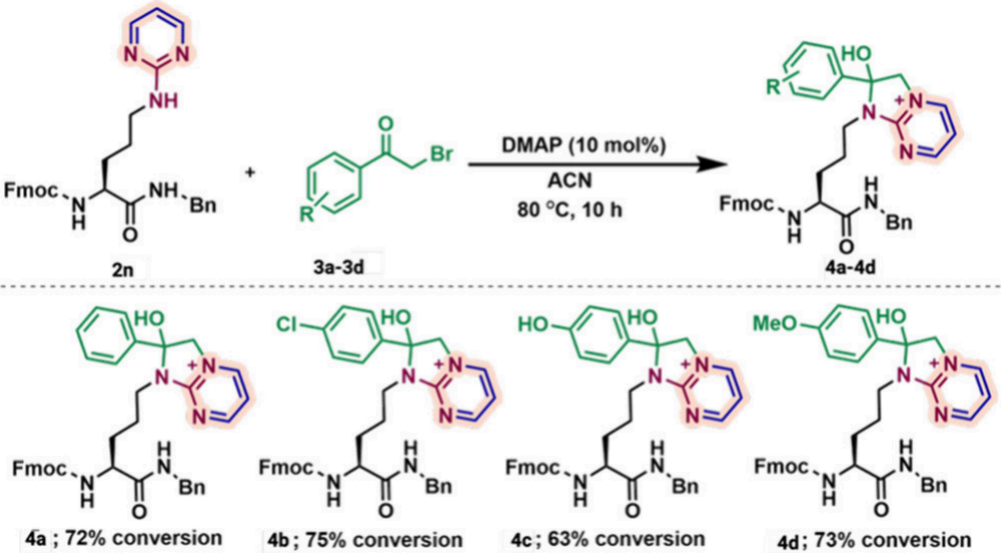
Late-stage functionalization of the amino pyrimidine arginine **2n** with 2-bromoacetophenone derivatives **3**. Unless otherwise noted, all reactions were carried out using **2n** (0.02 mmol, 1.0 equiv), **3a**–**3d** (0.02 mmol, 1.1 equiv) in acetonitrile (2 mL) at 80 °C for 10 h.

In summary, we have established a novel acid-mediated chemoselective strategy for the selective modification of arginine residues in peptides. Using malonaldehyde, guanidinium side chains were efficiently transformed into amino pyrimidine moieties with near-quantitative conversion and broad substrate scope. Undesired side products arising from other nucleophilic residues can be readily reversed with butylamine, underscoring the robustness and selectivity of the method. The resulting amino pyrimidine peptides exhibit significantly enhanced cellular permeability, while the amino pyrimidine motif also serves as a versatile handle for late-stage diversification into imidazo­[1,2-*a*]­pyrimidinium salts. This chemistry opens an unconventional route to expand the chemical space of peptides by harnessing arginine reactivity. Beyond efficient and selective modification, the method improves peptide stability and cellular uptake while enabling access to new heteroaromatic scaffolds. Together, these advances open avenues for designing peptide therapeutics with enhanced bioavailability, drug-like properties, and broader pharmacological potential.

## Supplementary Material



## Data Availability

The data underlying this study are available in the published article and its Supporting Information.

## Notes

General experimental procedures and characterization details, including HPLC, HRMS, and NMR spectra of all reported compounds (PDF)
