# Supplementary material for: Expanding Peptide Chemical Space via Acid-Mediated Arginine Modification
Source: Org Lett. 2025 Oct 30;27(45):12574–7. doi: 10.1021/acs.orglett.5c03977 (PMC12624831; doi:10.1021/acs.orglett.5c03977)
Supplement: Supplementary file 1 [file ol5c03977_si_001.pdf]

Supporting Information

Expanding Peptide Chemical Space *via* Acid-Mediated Arginine Modification

Pinki Sihag,[‡a] Minyoung Kwon,[‡a] Ankita Misra[a] and Monika Raj[a*]

[a]Department of Chemistry, Emory University, Atlanta, Georgia 30322, United States
Email: *monika.raj@emory.edu

Table of Contents

I. General. 2

II. Materials. 2

III. Purification. 2

IV. Instrumentation and sample analysis. 3

IVa. NMR 3

IVb. Analytical HPLC. 3

IVc. LC/MS. 3

IVd. HRMS. 3

V. Fmoc-Solid Phase Peptide Synthesis. 3

VI. BD FACSymphony™ Flow Cytometer. 4

VII. General Cell Culture Techniques. 4

VIII. Synthesis of malondialdehyde sodium salt[2] 5

IX. Conversion of N-benzyl guanidine to N-benzyl pyrimidin-2-amine 6

^{1}H NMR (400 MHz,DMSO) of compound N-benzyl pyrimidin-2-amine 6

^{13}C (101MHz, DMSO) of compound N-benzyl pyrimidin-2-amine 7

X. Supplementary Table 1. Optimization table for modification of peptide 1a with MDA 7

XI. Chemoselectivity studies on modification of peptides containing arginine and other reactive residues with MDA. 15

For Peptide **1b**: 16

For Peptide **1c**: 18

For Peptide **1d:** 21

For Peptide **1e**: 24

XII. Substrate scope of Modification of arginine containing peptides 1f-1k with MDA . 27

For Peptide 1f: 27

For Peptide 1g:.......... 29

For Peptide 1h:.......... 30

For Peptide 1i: 32

For Peptide 1j 34

For Peptide 1k:.......... 35

XIII Synthesis of chloroalkane carboxylate: 37

XIV Synthesis of Chloroalkane Tag Peptides ct-1l & 1m and ct-2l & 2m: 38

XV. Chloroalkane Permeability Assay 41

XVI. Late-stage functionalization of the MDA-modified protected arginine amino acid 44

XVII References:.......... 51

I. General. All commercial materials (Sigma-Aldrich, TCI America, Oakwood Chemical) were used without further purification. All solvents were reagent or HPLC (Fisher) grade. All reactions were performed under air in glass dram vials. Yields refer to chromatographically pure compounds; percent yields were obtained by comparing HPLC peak areas of products and starting materials. HPLC and MS were used to monitor reaction progress, and product elucidation was done using HRMS and NMR.

II. Materials. Fmoc-amino acids, Rink amide resin, 3-[bis(dimethylamino)methyliumyl]-3H-benzotriazol-1-oxide hexafluorophosphate (HBTU), 1-hydroxy-7-azabenzotriazole (HOAt), N,N′-iisopropylcarbodiimide (DIC) was obtained from CreoSalus (Louisville, Kentucky) and N,N-diisopropylethylamine (DIPEA) was obtained from TCI America. Piperidine, trifluoroacetic acid (TFA), were obtained from Alfa Aesar (Ward Hill, Massachusetts). N,N-dimethylformamide (DMF), dichloromethane (DCM), methanol (MeOH), HCl, 1,1,3,3 tetramethoxypropane, N-Benzylguanidine, Acetic Anhydride and acetonitrile (ACN) were obtained from VWR (100 Matsonford Road Radnor, Pennsylvania). All small molecules were obtained from commercial vendors.

III. Purification. HPLC: Purification of peptide starting materials was performed using high performance liquid chromatography (HPLC) on an Agilent 1100 series HPLC equipped with a C-18 reverse phase column with a particle size of 5 µm. All separations involved a mobile phase of water (solvent A) and acetonitrile (solvent B). The HPLC method used a linear gradient of 0-80% solvent B over 30 minutes at ambient temperature with a flow rate of 1 mL/min. The eluent was monitored by absorbance at 220 nm.

IV. Instrumentation and sample analysis.

IVa. NMR. ^1H and ^{13}C spectra were acquired at 25 °C using an Agilent DD2 (400 or 600 MHz) spectrometer with a 3-mm He triple resonance (HCN) cryoprobe. All ^1H NMR chemical shifts (δ) were referenced relative to the residual DMSO-d_6 peak at 2.50 ppm or $CDCl_3$ at 7.26 ppm. ^{13}C NMR chemical shifts were referenced to DMSO-d_6 at 39.52 ppm or $CDCl_3$ at 77.16 ppm. ^{13}C NMR spectra were proton decoupled. NMR spectral data are reported as chemical shift (multiplicity, coupling constants (J), integration). Multiplicity is reported as follows: singlet (s), broad singlet (br s), doublet (d), doublet of doublets (dd), doublet of triplets (td), triplet (t) and multiplet (m). Coupling constant (J) in hertz (Hz).

IVb. Analytical HPLC. Analytical HPLC chromatography (HPLC) was performed on an Agilent 1100 series HPLC equipped with a 4.6 x 150 mm RediSep Prep C_{18} Aq, 100 Å, 5 µm column. The reaction was monitored by analytical reverse phase HPLC using a gradient of water versus acetonitrile in linear gradients with a constant flow rate of 1 mL/min. Separations involved a mobile phase of 0.1% formic acid in water (solvent A) and 0.1 % formic acid in acetonitrile (solvent B) or mobile phase of water (solvent A) and acetonitrile (solvent B). The eluent was monitored with a detection wavelength of 220 nm.

HPLC Method A: Gradient: 0 to 80 % B (0.1% formic acid in ACN) in 30 min; 80-100 % B in 31-35 min at a flow rate of 1 mL/min.

HPLC Method B: Gradient: 0 to 30 % B (0.1% formic acid in ACN) in 30 min; 30-100 % B in 31-35 min at a flow rate of 0.5 mL/min.

IVc. LC/MS. High resolution LC-MS conditions for all purified peptides: Analyses were performed on an ultraperformance LC system (ACQUITY, Waters Corp., USA) coupled with a quadrupole 3 time-of-flight mass spectrometer (Q-ToF Premier, Waters) with electrospray ionization (ESI) in positive mode using Mass lynx software (V4.1) or high-performance LC system (Agilent, 1100 series) coupled with triple quadrupole.

LC-MS (Agilent technologies 6460) with electrospray ionization (ESI) in positive mode using Agilent mass hunter (10.0). Unless otherwise mentioned a sample was injected either onto a C4 column (Phenomenex Aeris™ 3.6 µm WIDEPORE C_4 200 Å, LC Column 50 x 2.1 mm) with a 400 µL/min flow rate of mobile phase of solution A (90 % H_2O, 10 % acetonitrile and 0.1 % formic acid (FA)) and solution B (95 % acetonitrile, 5 % H_2O, and 0.1 % formic acid) beginning gradient- Time- 0 min 10 % B; 5 min 28 % B; 20 min 38 % B; 22 min 90 % B; C18 column (ACQUITY UPLC BEH 1.7 µm 1x 50 mm) with a 200 µL/min flow rate of mobile phase of solution A (90 % H_2O, 10 % acetonitrile and 0.1 % formic acid) and solution B (90 % acetonitrile, 10 % H_2O, and 0.1 % formic acid) beginning gradient- Time- 1 min 0% B; 1-10 min 100% B for chromatography analysis (or) directly injected with mobile phase 90 % H_2O: 10 % ACN, 0.1% formic acid at 400 µL/min flow rate in ESI positive mode.

IVd. HRMS. High resolution MS data were acquired on Thermo Exactive Plus using a heated electrospray source. The solution was infused at a rate of 10-25 µL/min/electrospray using 3.3 KV. The typical settings were Capillary temp 320 °C. S-lens RF level was between 30-80 with an AGC setting of 1 E6. The maximum injection time as set to 50 ms. Spectra were taken at 140,000 resolutions at *m/z* 200 using Tune software and analyze with Thermo's Freestyle software.

V. Fmoc-Solid Phase Peptide Synthesis.[1] Peptides were synthesized manually on a 0.25 mmol scale using Rink amide resin. Resin was swollen with DCM for 1 h at room temperature. Fmoc was deprotected using 20% piperidine–DMF for 5 min to obtain a deprotected peptide-resin. First, Fmoc protected amino acid (1.25 mmol/5 equiv.) was coupled using HOAt (1.25 mmol/5 equiv.) and DIC (1.25 mm/5 equiv.) in DMF for 15 min at room temperature. Fmoc-protected amino acids (0.75 mmol/3 equiv.) were sequentially coupled on the resin using HBTU (0.75 mmol/3 equiv.) and DIEA (1.5 mmol/6 equiv.) in DMF for 5 min

at room temperature. Peptides were synthesized using standard protocols. Peptides were cleaved from the resin using a cocktail of 95:5, trifluoroacetic acid:water for 2 h. The resin was removed by filtration, and the resulting solution was concentrated via air flow. The residue was washed with cold ether (3 x 10 mL), precipitating a white solid. Ether was removed via vacuum overnight. The white solid was diluted with ACN/H_2O mixture. The resulting solution was purified by HPLC.

VI. BD FACSymphony™ Flow Cytometer. Flow cytometry analysis was performed on a BD FACSymphony™ system equipped with decagon (2-10 PMTs) or cascade (2-20 PMTs) detector arrays. All analyses utilized a combination of lasers: violet (405 nm, 100 mW), blue (488 nm, 100 mW), UV (355 nm, 100 mW), yellow-green (561 nm, 100 mW), and red (637 nm, 140 mW), with additional wavelength options from 375-980 nm at 20- 1000 mW power levels. The system operates with a pressure-regulated fluidics system (10L capacity) and digital signal processing enabling simultaneous 28-parameter detection. Signal detection combines PMTs (side scatter/fluorescence) and photodiodes (forward scatter), with longpass dichroic and bandpass filters for wavelength selection. Data acquisition and analysis was performed using BD FACSDiva™ software with Cytometer Setup and Tracking (CS&T) for quality control and standardization.YG 586 as a detector channel. The absorbance of the molecule is 533 nm and emission is 588 nm.

VII. General Cell Culture Techniques. Cells were maintained at 37 °C and 5% CO2. T-47D cells were cultured in RPMI supplemented with 10% (V/V) fetal bovine serum (FBS), 1% (V/V) penicillin/streptomycin (100 µg/mL), and amphotericin-b (2 µg/mL). BT474 cells were cultured in DMEM supplemented with 10% (V/V) fetal bovine serum (FBS), 1% (V/V) penicillin/streptomycin (100 µg/mL), and amphotericin-b (2 µg/mL). MCF10A cells were cultured in DMEMF12 supplemented with 5% (V/V) fetal bovine serum (FBS), insulin (10 µg/mL), epidermal growth factor (10 ng/mL), 1% (V/V) penicillin/streptomycin (100 µg/mL), hydrocortisone (0.5 µg/mL), cholera toxin (1 ng/mL), and amphotericin-b (2 µg/mL). The experiments were conducted using HEK cells stably expressing a HaloTag-GFP-mitochondrial localized construct (HEK cells), which were constructed as previously described (Ballister et al., 2014). The cells were maintained in high-glucose DMEM supplemented with 10% fetal bovine serum (FBS), 1% penicillin/streptomycin, and 1 µg/mL puromycin. Initial cell preparation involved thawing HEK cells in 20 mL of antibiotic-free cell culture media (DMEM + 10% FBS), followed by one passage and selection with 20 µg/mL puromycin in cell culture media. After recovery from selection and reaching near-confluency, the cells were deemed ready for CAPA experiments. Cell culture conditions were maintained at 37°C with 5% CO2, with regular passaging every 2–3 days. CAPA experiments were routinely conducted when cells demonstrated healthy morphology and robust HaloTag-GFP expression, typically between passages 3 and 14.

VIII. Synthesis of malondialdehyde sodium salt[2]

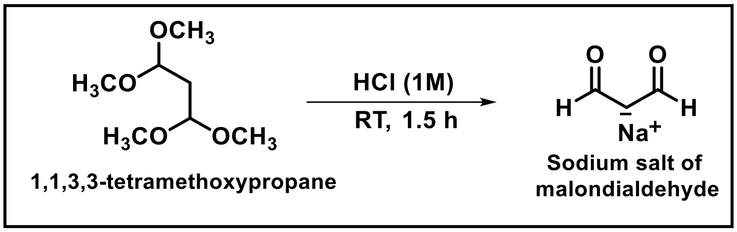

In an oven-dried 250 mL round-bottom flask with stir bar, 15 mL of 1,1,3,3-tetramethoxypropane is added then 8 mL of 1 M HCl solution in water at room temperature. The reaction mixture was stirred for 90 minutes till the two liquid phases became miscible and colorless liquid turned pale yellow. Then the pH of the solution was adjusted to ~7-8 with NaOH solution (5 M in water) to limit polymerization. The intensity of the solution color is dependent on the MDA polymerization. MDA was precipitated by cooling the solution to 0 °C, in 150 mL of acetone for 1 hour. Solid MDA salt was separated *via* vacuum filtration. **^{1}H NMR (400 MHz, D_2O)**: δ 8.67 – 8.58 (m, 2H), 5.35 – 5.24 (m, 1H).

^{1}H NMR (400 MHz, $CDCl_3$) of compound **sodium salt of malondialdehyde**

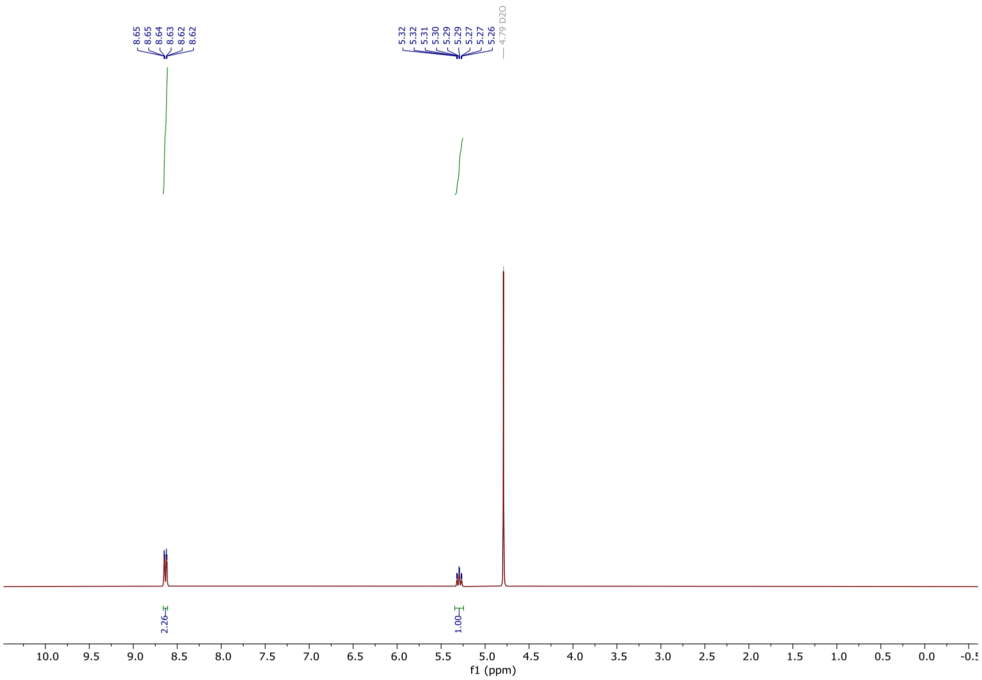

IX. Conversion of N-benzyl guanidine to N-benzyl pyrimidin-2-amine

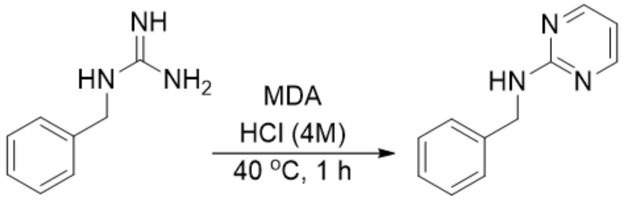

In an oven-dried 50 mL round-bottom flask equipped with magnetic stirrer, N-benzylguanidine hydrochloride (40 mg, 0.216 mmol, 1 eq), malondialdehyde (91 mg, 0.27 mmol, 20 eq) were added. Followed by dissolving them into 4mL MeOH, and 1mL of 12 M HCl were added. The mixture was allowed to react at 40°C for 1 hour. After the reaction was completed, 4 M NaOH was used to quench the reaction and extracted with 3*20 mL CH_2Cl_2 and brine. The organic layer was dried using sodium sulfate and solvent was removed via rotavapor. The final product was then purified using column chromatography to obtain the N-benzyl pyrimidin-2-amine product[3].

^{1}H NMR (400 MHz, DMSO): δ 8.25 (d, J = 4.8 Hz, 2H), 7.68 (t, J = 6.4 Hz, 1H), 7.33 – 7.26 (m, 4H), 7.29 – 7.15 (m, 1H), 6.56 (t, J = 4.8 Hz, 1H). **^{13}C NMR (101 MHz, DMSO)**: δ 162.3, 158.0, 140.4, 128.1, 127.0, 126.5, 110.2, 43.8.

^{1}H NMR (400 MHz,DMSO) of compound N-benzyl pyrimidin-2-amine

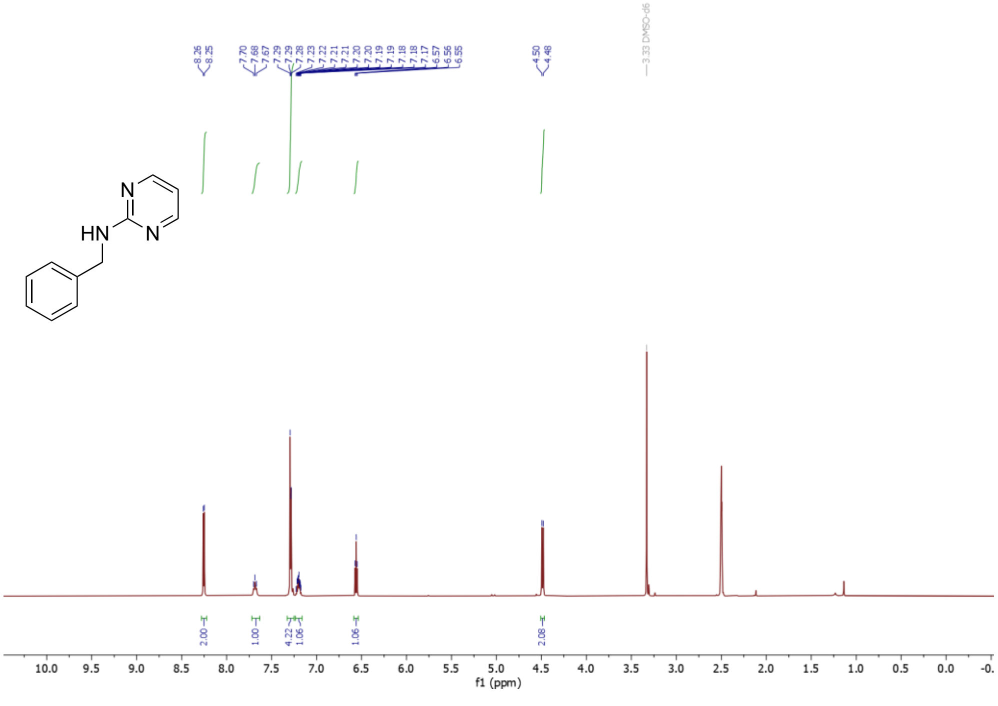

^{13}C (101MHz, DMSO) of compound N-benzyl pyrimidin-2-amine

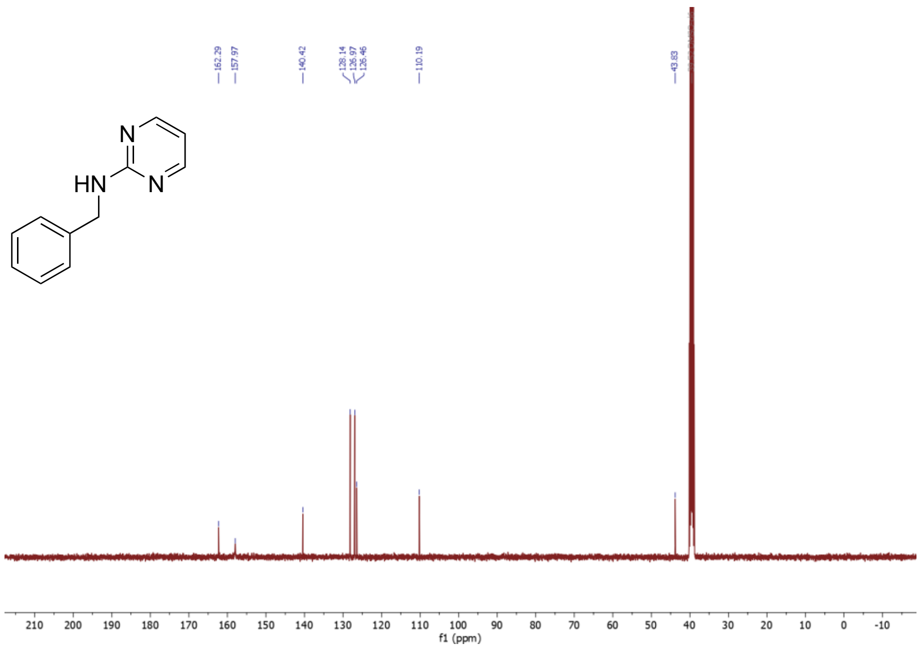

X. Supplementary Table 1. Optimization table for modification of peptide 1a with MDA

General procedure A: In a one-dram vial, peptide **1a** (1.0 mg, 0.002 mmol, 1 equiv.) and different equivalents of MDA were added. Followed by 500 µL of varying solvents (table S1). The reaction was allowed to proceed for different durations at room temperature then diluted to 2 mL with buffer (pH 7). Subsequently, the reaction mixture was injected into the HPLC to determine the % conversion of peptide **1a** to the labeled peptides **2a** and their mass confirmed with LC-MS. HPLC analysis was carried out utilizing **HPLC Method A** at detection wavelength 220 nm. Refer to optimization table below for reaction conditions. The masses of the products were confirmed with LC-MS.

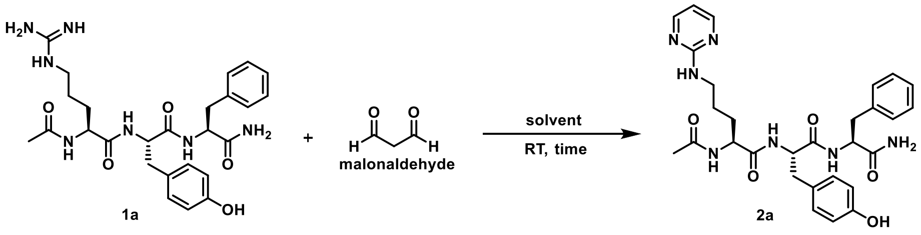

Table S1: Optimization table

Entry	Solvent	MDA	% Conversion[b]
1	H_2O	75 equiv.	0
2	Na_2CO_3 (pH 10)	75 equiv.	0
3	1M NaOH	75 equiv.	0
4	$C_6H_8O_7$ (pH 4)	75 equiv.	0
5	6 M HCl	75 equiv.	13
6[c]	6 M HCl	75 equiv.	23
7	6 M HCl	100 equiv.	66
8	8 M HCl	75 equiv.	26
9[c]	8 M HCl	75 equiv.	33
10	12 M HCl	25 equiv.	75
11	12 M HCl	50 equiv.	90
12	12 M HCl	75 equiv.	96
13[d]	12 M HCl	100 equiv.	>99
14	TFA	100 equiv.	0

[a]Unless otherwise noted, all reactions were carried out using **1a** (0.002 mmol, 1.0 equiv), MDA (0.2 mmol, 100 equiv.) in 500 µL solvent at room temperature for 1 h. [b]Conversion is determined by HPLC at 220 nm. [c] Reaction time is 2 h. [d]Optimized reaction conditions.

HPLC Trace of Ac-RYF-CONH$_2$ Starting Peptide 1a at 220 nm

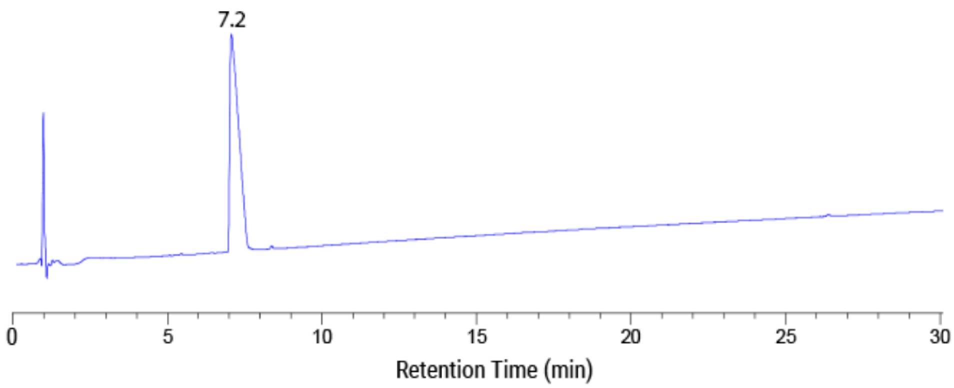

HRMS Trace of Peak at 7.2 min

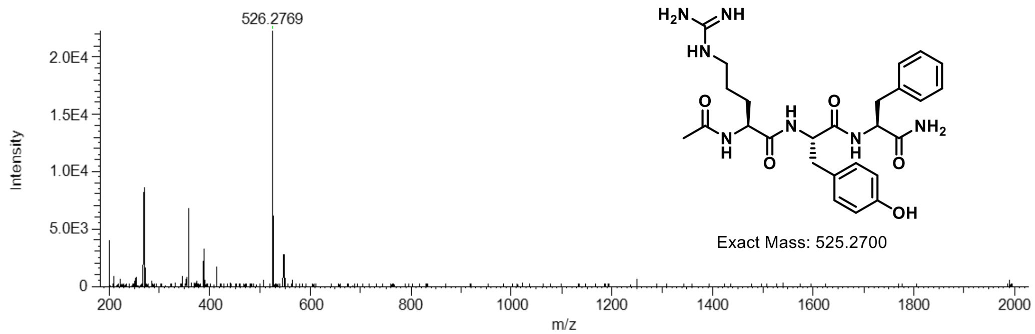

Ac-RYF-CONH$_2$ starting peptide 1a. LCMS for $C_{26}H_{35}N_7O_5$: *m/z* 526.2769 (calcd $[M+H]^+$ = 526.2772) (HPLC analysis at 220 nm). Retention time in HPLC: 7.2 min

HPLC Trace of Ac-RYF-CONH$_2$ Reaction Mixture after Entry 1 at 220 nm

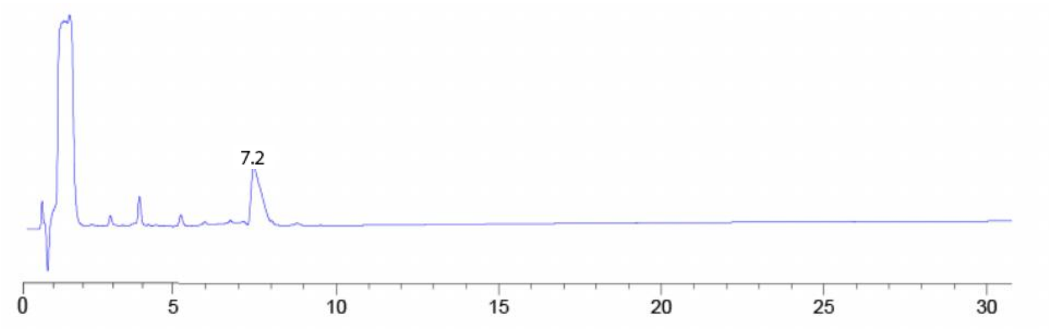

HPLC Trace of Ac-RYF-CONH$_2$ Reaction Mixture after Entry 2 at 220 nm

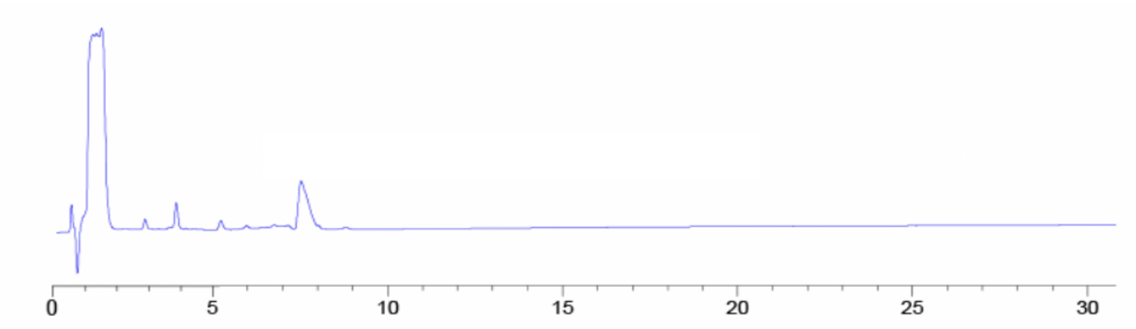

HPLC Trace of Ac-RYF-CONH$_2$ Reaction Mixture after Entry 3 at 220 nm

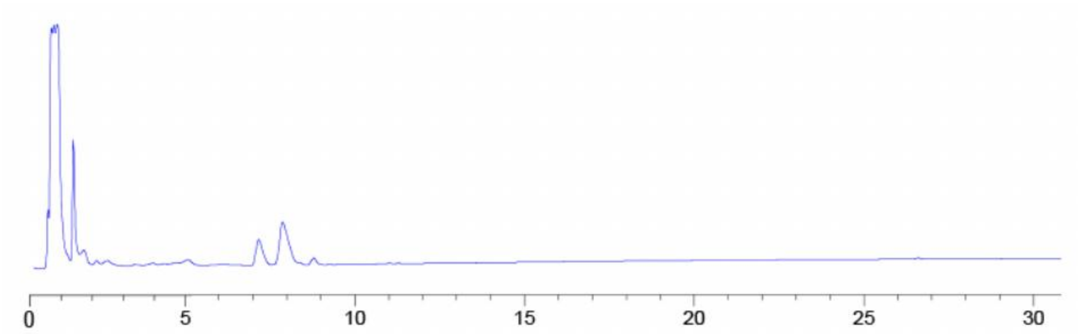

HPLC Trace of Ac-RYF-CONH$_2$ Reaction Mixture after Entry 4 at 220 nm

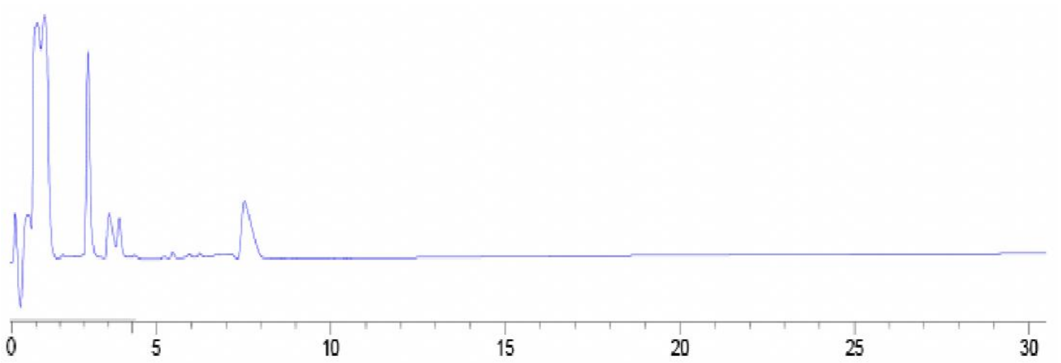

HPLC Trace of Ac-RYF-CONH$_2$ Reaction Mixture after Entry 5 at 220 nm

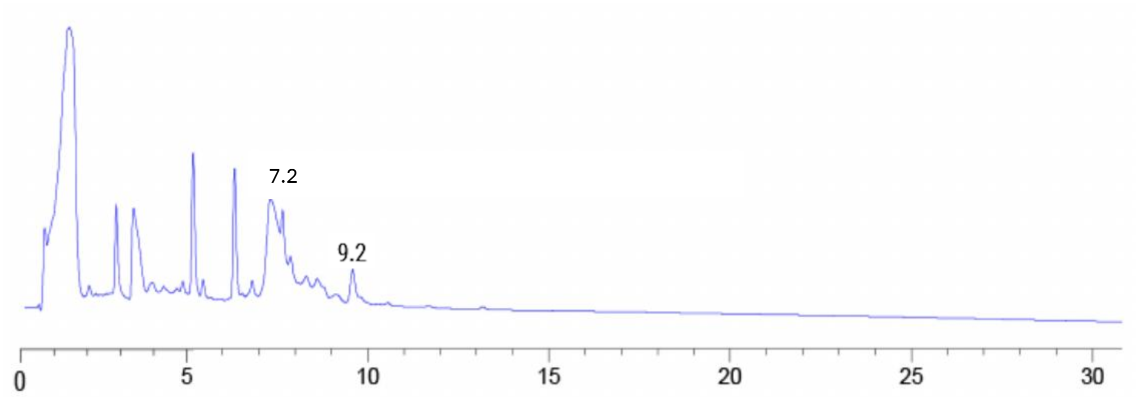

HPLC Trace of Ac-RYF-$CONH_2$ Reaction Mixture after Entry 6 at 220 nm

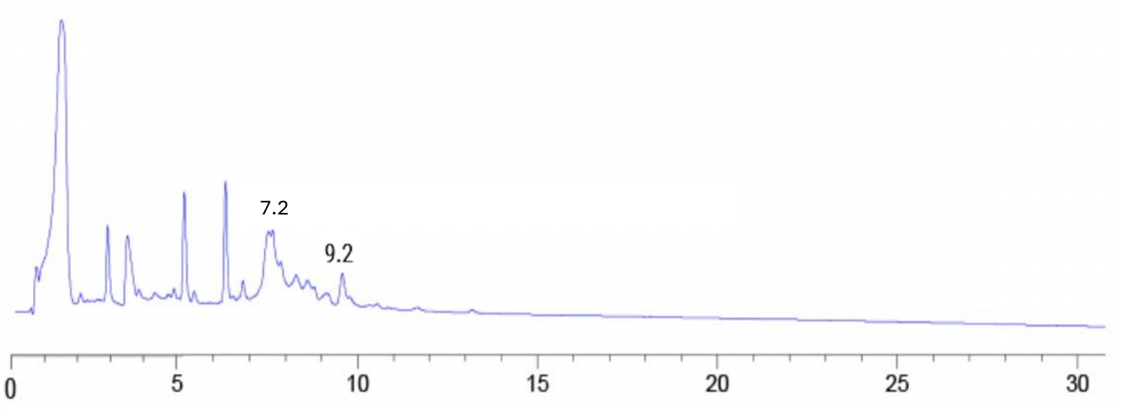

HPLC Trace of Ac-RYF-$CONH_2$ Reaction Mixture after Entry 7 at 220 nm

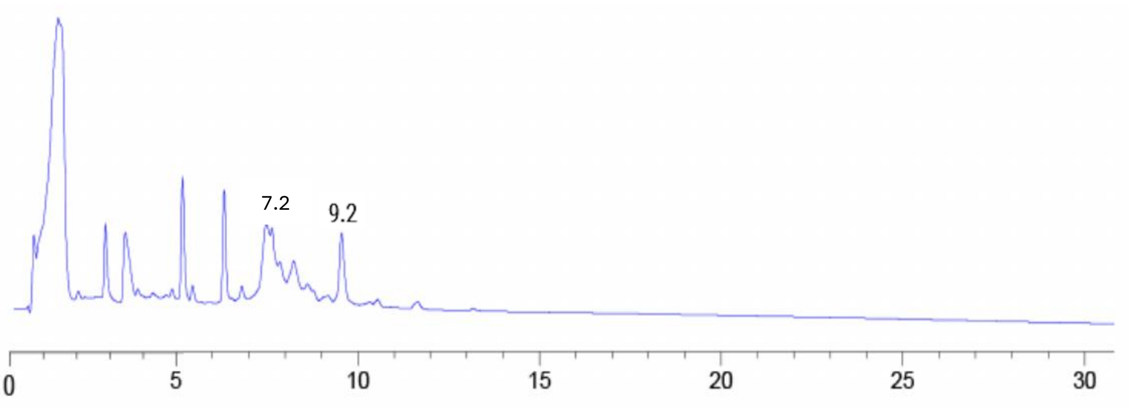

7HPLC Trace of Ac-RYF-$CONH_2$ Reaction Mixture after Entry 8 at 220 nm

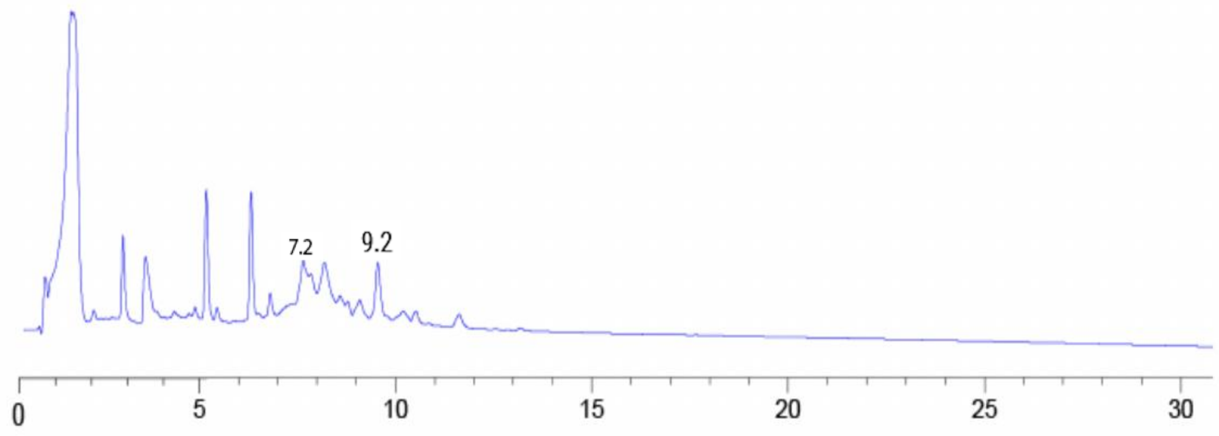

HPLC Trace of Ac-RYF-CONH$_2$ Reaction Mixture after Entry 9 at 220 nm

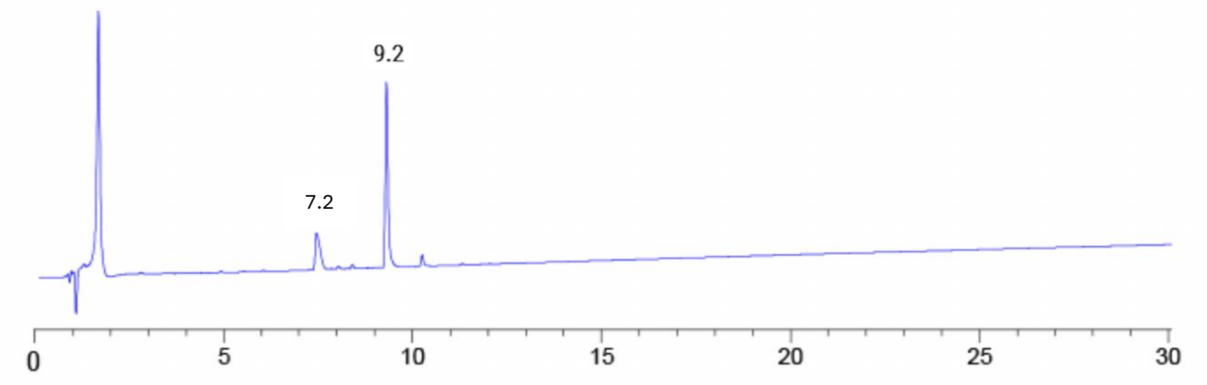

HPLC Trace of Ac-RYF-CONH$_2$ Reaction Mixture after Entry 10 at 220 nm

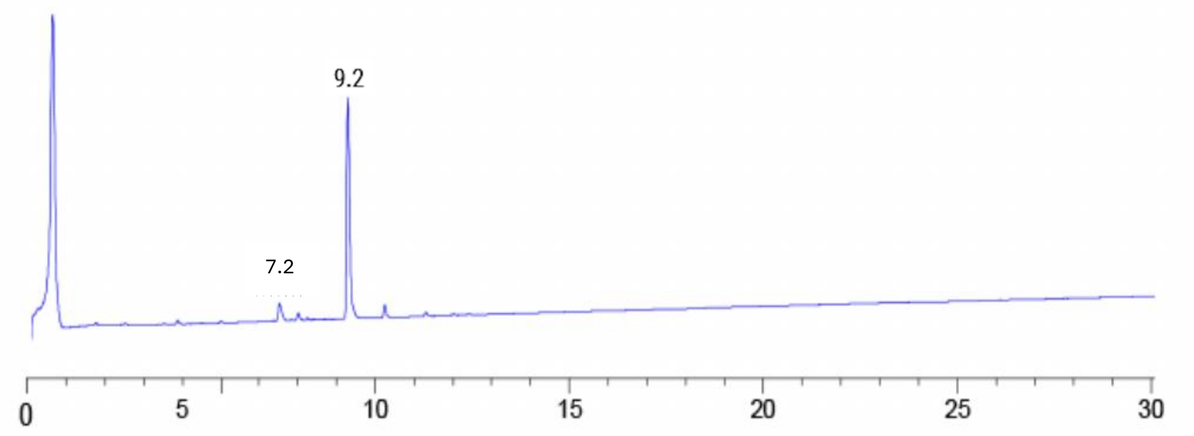

HPLC Trace of Ac-RYF-CONH$_2$ Reaction Mixture after Entry 11 at 220 nm

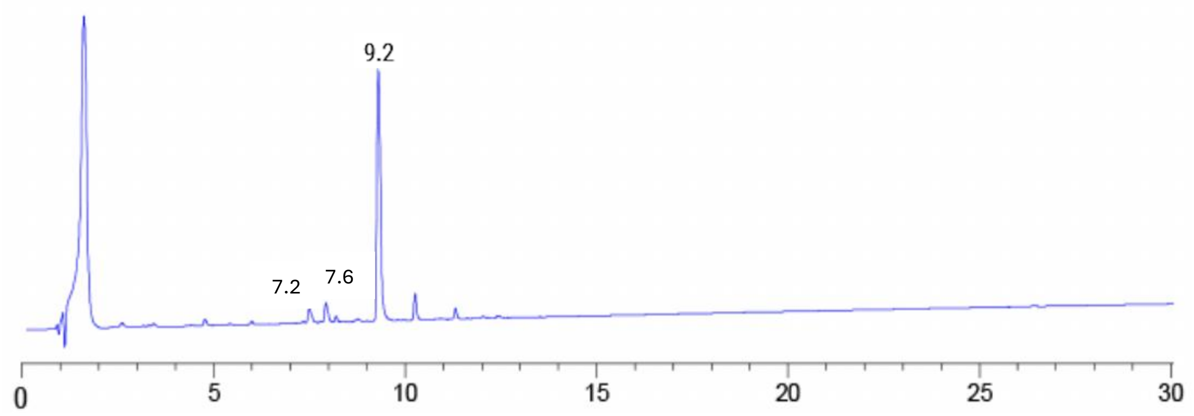

HPLC Trace of Ac-RYF-CONH$_2$ Reaction Mixture after Entry 12 at 220 nm

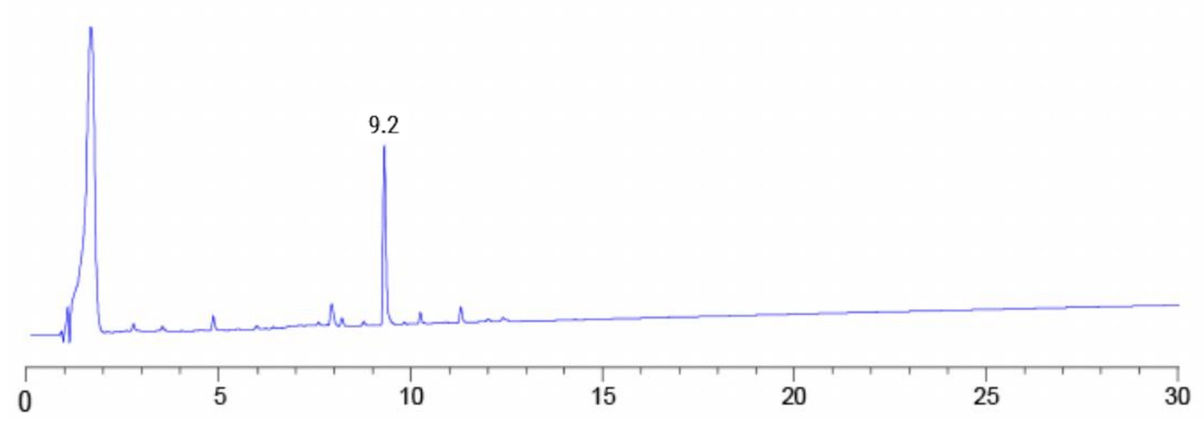

HPLC Trace of Ac-RYF-CONH$_2$ Reaction Mixture after Entry 14 at 220 nm

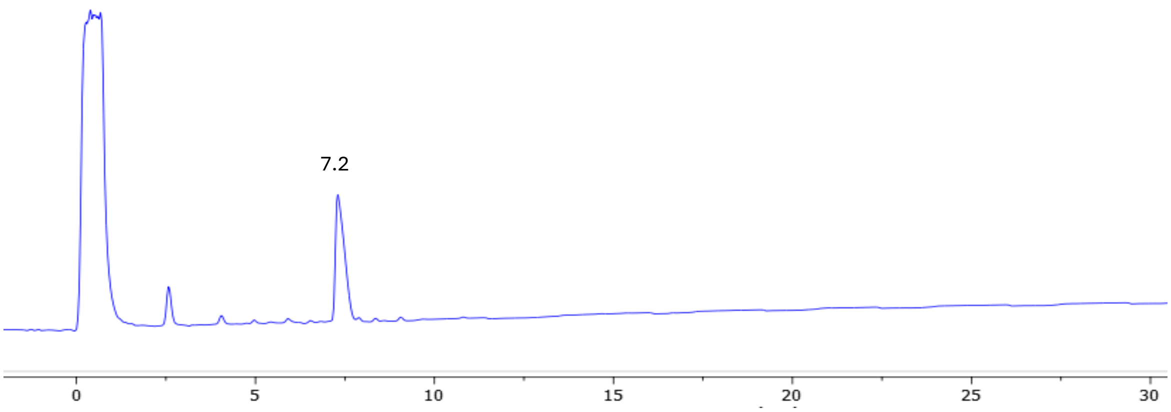

HRMS Trace of Peak at 7.6 min

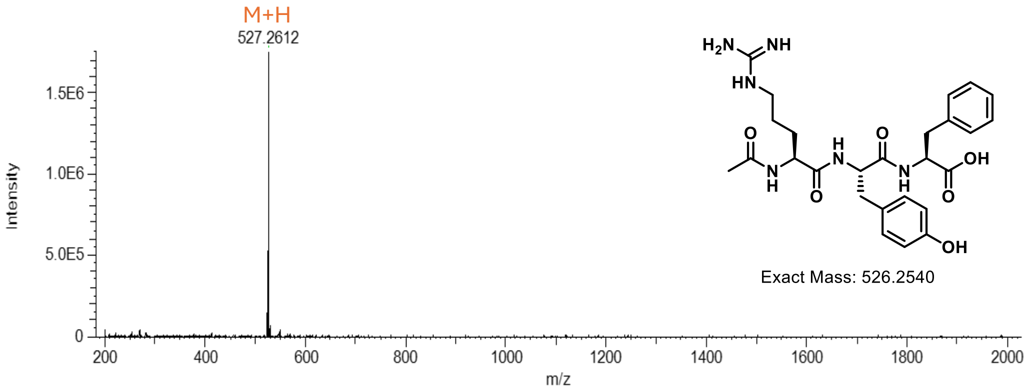

Ac-RYF-CONH$_2$ hydrolyzed peptide 1a'. LCMS for $C_{26}H_{35}N_6O_6$: *m/z* 527.2612 (calcd $[M+H]^+$ = 527.2613), (HPLC analysis at 220 nm). Retention time in HPLC: 7.6 min.

HRMS Trace of Peak at 9.2 min

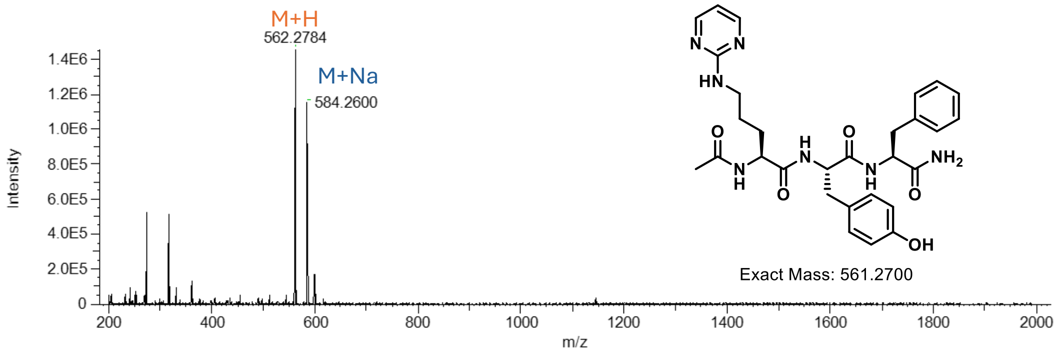

Ac-RYF-CONH$_2$ labeled product 2a. LCMS for $C_{29}H_{36}N_7O_5$: *m/z* 562.2784 (calcd $[M+H]^+$ = 562.2772), *m/z* 584.2600 (calcd $[M+Na]^+$ = 584.2592) (HPLC analysis at 220 nm). Retention time in HPLC: 9.2 min.

Reaction of 1b with acetylacetone:

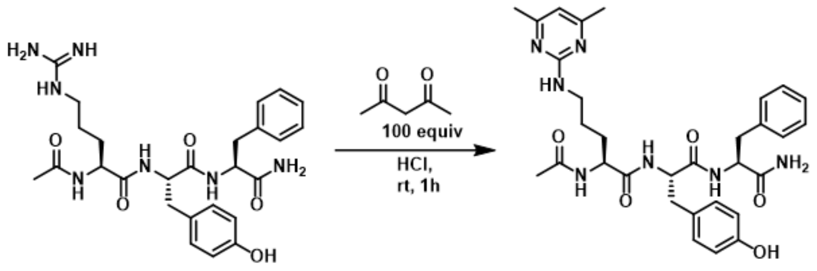

HPLC trace of reaction of Ac-RYF with acetylacetone in 220 nm.

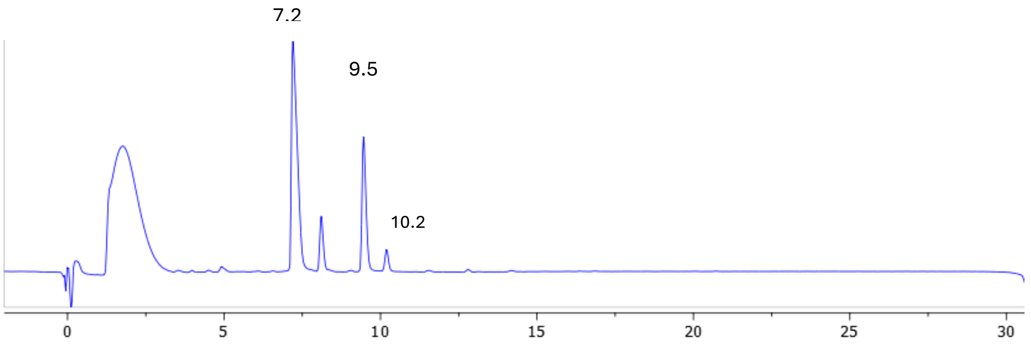

HRMS Trace of Peak at 9.5 min

Ac-RYF-CONH$_2$ acetylacetone labeled product: LCMS for $C_{31}H_{40}N_7O_5$: *m/z* 590.3075 (calcd $[M+H]^+$ =590.3085) (HPLC analysis at 220 nm). Retention time in HPLC: 9.5 min.

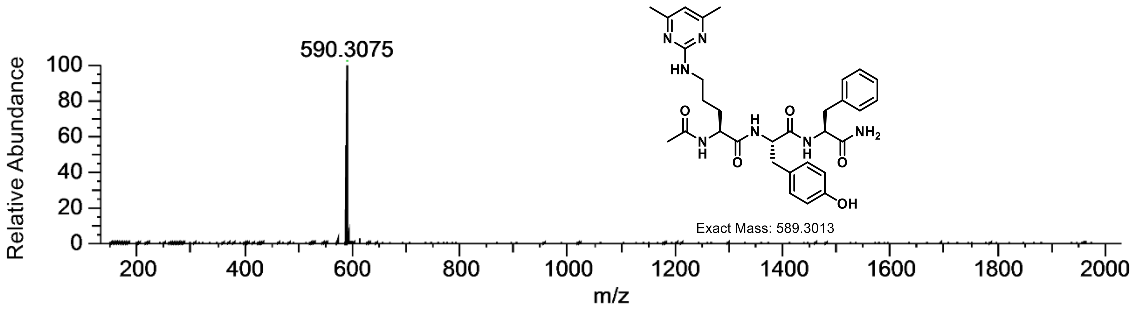

Ac-RYF-COOH acetylacetone labeled hydrolyzed product. LCMS for $C_{31}H_{39}N_6O_6$: *m/z* 591.2919 (calcd $[M+H]^+$ =591.2926) (HPLC analysis at 220 nm). Retention time in HPLC: 10.2 min.

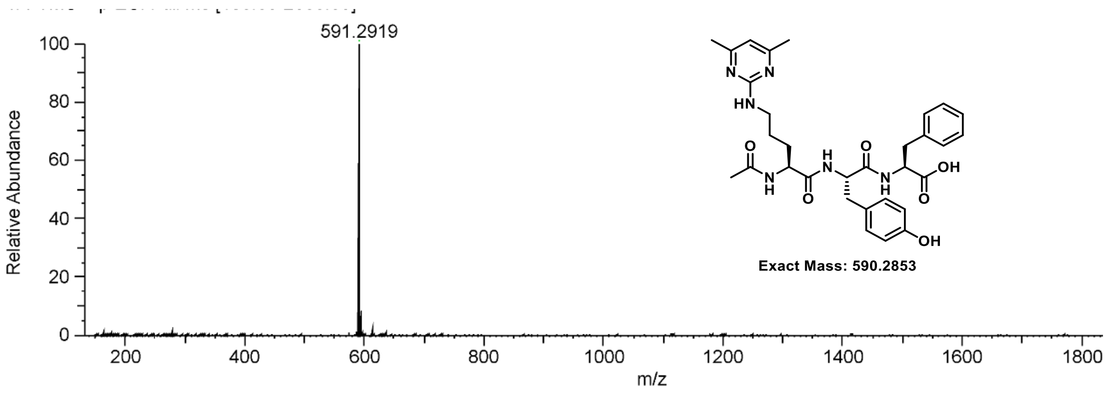

XI. Chemoselectivity studies on modification of peptides containing arginine and other reactive residues with MDA.

In a one-dram vial equipped with a magnetic stirrer, the starting peptide (**1b-1e**) (0.002 mmol, 1 equiv), malondialdehyde (MDA) (0.2 mmol, 100 equiv) was added. Followed by 500 µL of 12M HCl. The reaction was allowed to proceed for 1 hour at room temperature then diluted to 2 mL with buffer (pH 7). For reversing the unwanted side products, the reaction was diluted to 2 mL and pH was raised to 6 by using 4M NaOH solution. N-butylamine (150 mmol, 75 eq) was added, and the reaction was diluted to 2 mL and pH was raised 6 using 4M NaOH solution. The reaction is allowed to proceed for 3 hours at room temperature. After 3 h, the reaction mixture was injected into the HPLC to determine the % conversion of peptide **1b-1e** and product mass confirmed with LC-MS. HPLC analysis was carried out utilizing **HPLC Method A** at detection wavelength 220 nm. The masses of the products were confirmed with LC-MS.

For Peptide **1b**:

HPLC Trace of Ac-WRG-CONH$_2$ Starting Peptide 1b at 220 nm

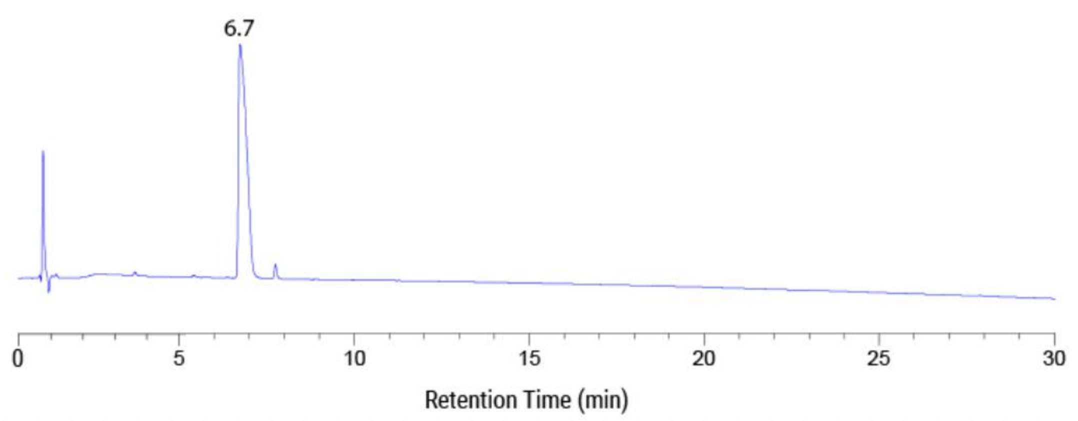

HRMS Trace of Peak at 6.7 min

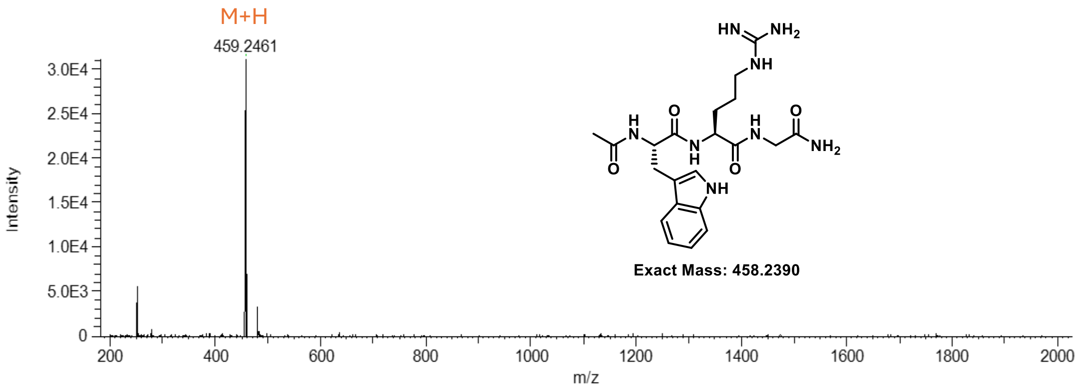

Ac-WRG-CONH$_2$ starting peptide 1b. LCMS for $C_{21}H_{31}N_8O_4$: *m/z* 459.2461 (calcd $[M+H]^+$ = 459.2463) (HPLC analysis at 220 nm). Retention time in HPLC: 6.7 min.

HPLC Trace Ac-WRG-CONH$_2$ Reaction Mixture at 220 nm

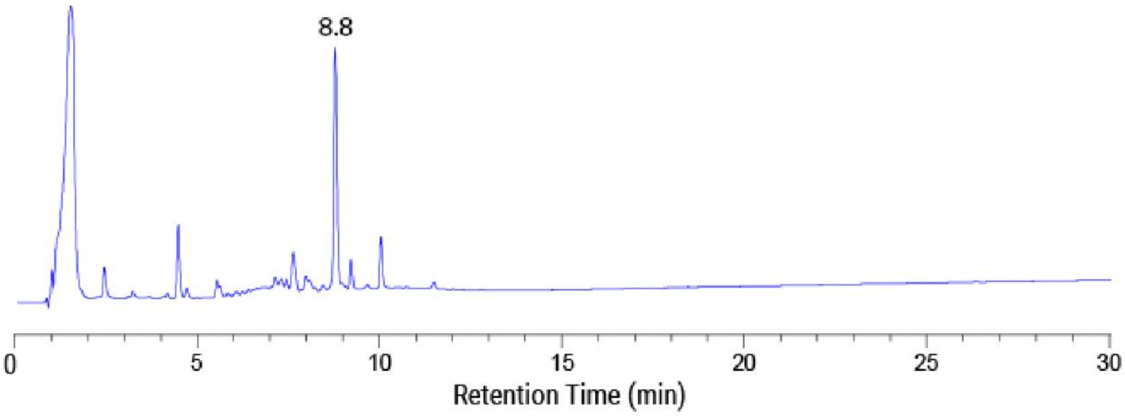

HRMS Trace of Peak at 8.8 min

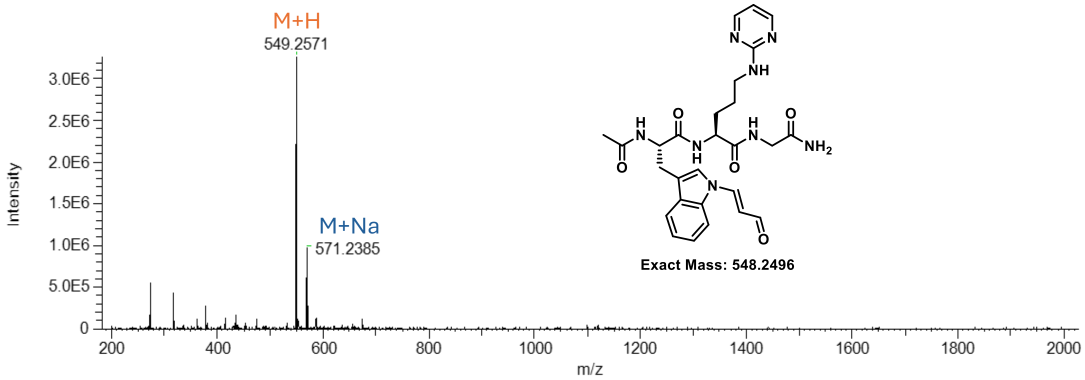

Ac-WRG-CONH$_2$ intermediate 2b'. LCMS for $C_{27}H_{33}N_8O_5$: *m/z* 549.2571 (calcd $[M+H]^+$ = 549.2568), *m/z* 571.2385 (calcd $[M+Na]^+$ = 571.2388) (HPLC analysis at 220 nm). Retention time in HPLC: 8.8 min

HPLC Trace Ac-WRG-CONH$_2$ Reaction Mixture after addition of BuNH$_2$ at 220 nm

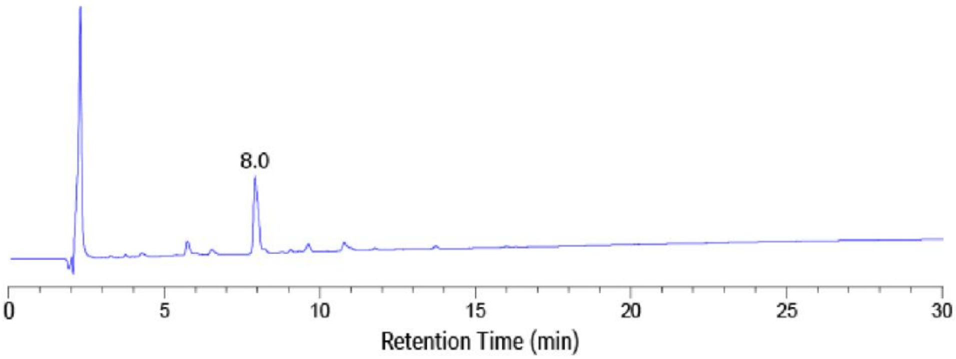

HRMS Trace of Peak at 8.0 min

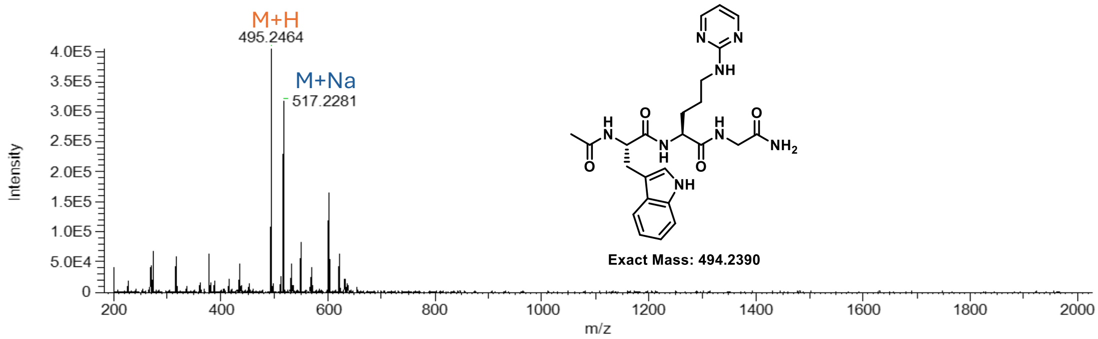

Ac-WRG-CONH$_2$ labelled product 2b. LCMS for $C_{24}H_{31}N_8O_4$: *m/z* 495.2464 (calcd $[M+H]^+$ = 495.2463), *m/z* 517.2281 (calcd $[M+Na]^+$ = 517.2282) (HPLC analysis at 220 nm). Retention time in HPLC: 8.0 min.

For Peptide **1c**:

HPLC Trace of Ac-FRRK-CONH$_2$ Starting Peptide 1c at 220 nm

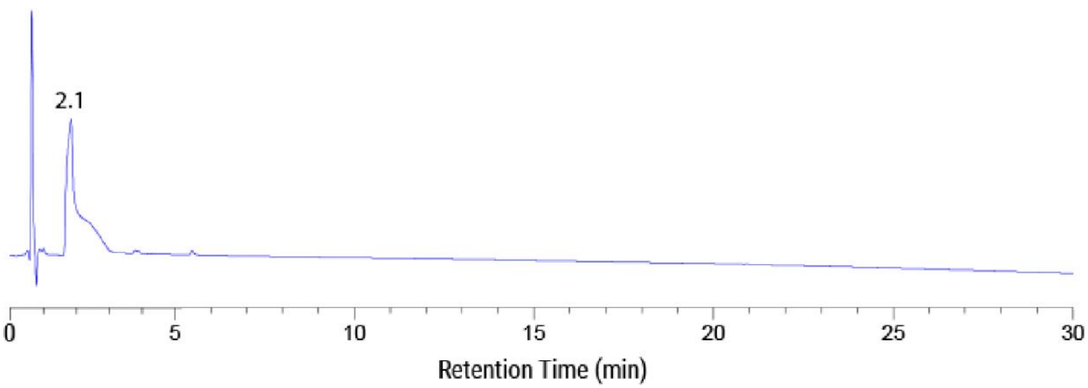

HRMS Trace of Peak at 2.1 min

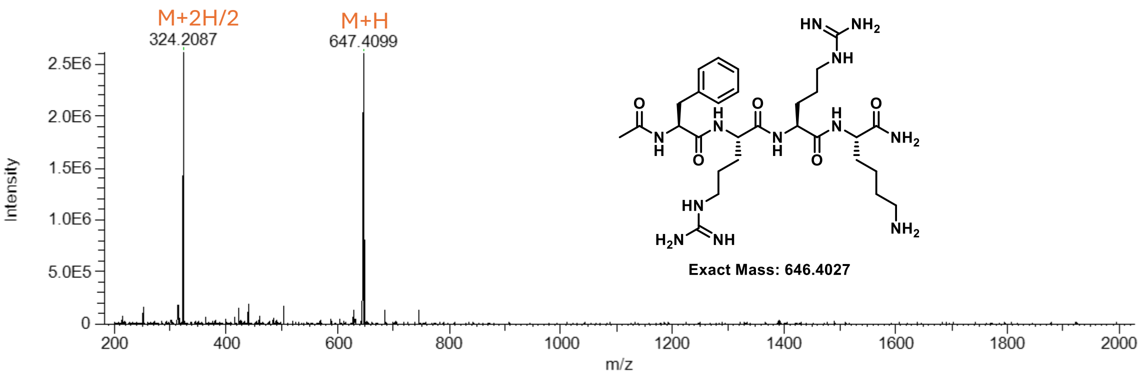

Ac-FRRK-CONH₂ starting peptide 1c. LCMS for $C_{29}H_{51}N_{12}O_5$: *m/z* 647.4099 (calcd $[M+H]^+$ = 647.4100), *m/z* 324.2087 (calcd $[M+2H]/2^+$ = 324.2086) (HPLC analysis at 220 nm). Retention time in HPLC: 2.1 min.

HPLC Trace of Ac-FRRK-CONH₂ Reaction Mixture at 220 nm

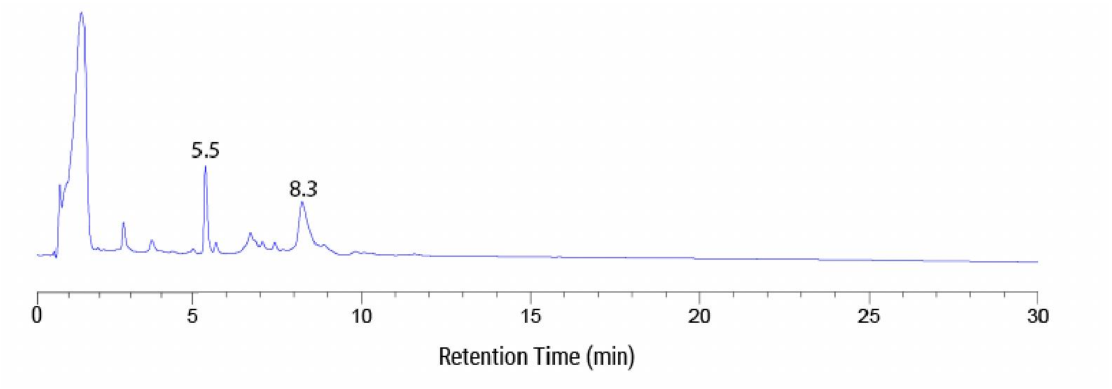

HRMS Trace of Peak at 5.5 min

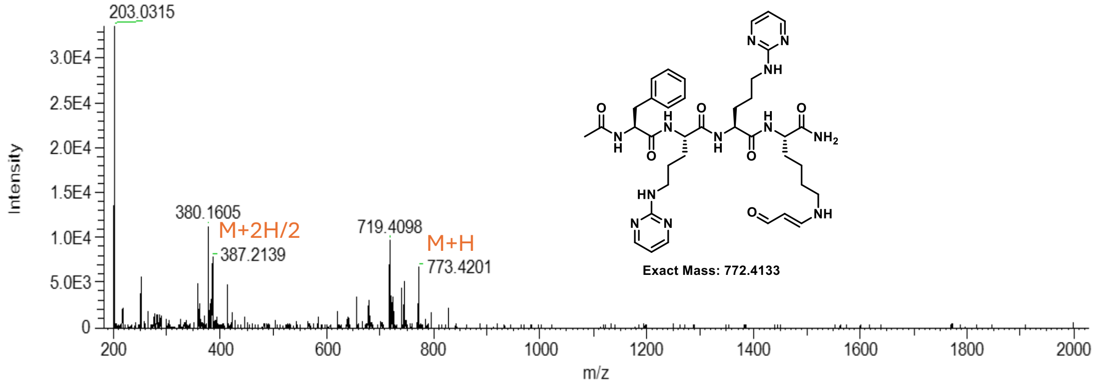

Ac-FRRK-CONH₂ intermediate peptide 2c'. LCMS for $C_{38}H_{53}N_{12}O_6$: *m/z* 773.4201 (calcd $[M+H]^+$ = 773.4206), *m/z* 387.2139 (calcd $[M+2H]/2^+$ = 387.2139) (HPLC analysis at 220 nm). Retention time in HPLC: 5.5 min

HPLC Trace of Ac-FRRK-CONH$_2$ Reaction Mixture after addition of BuNH$_2$ at 220 nm

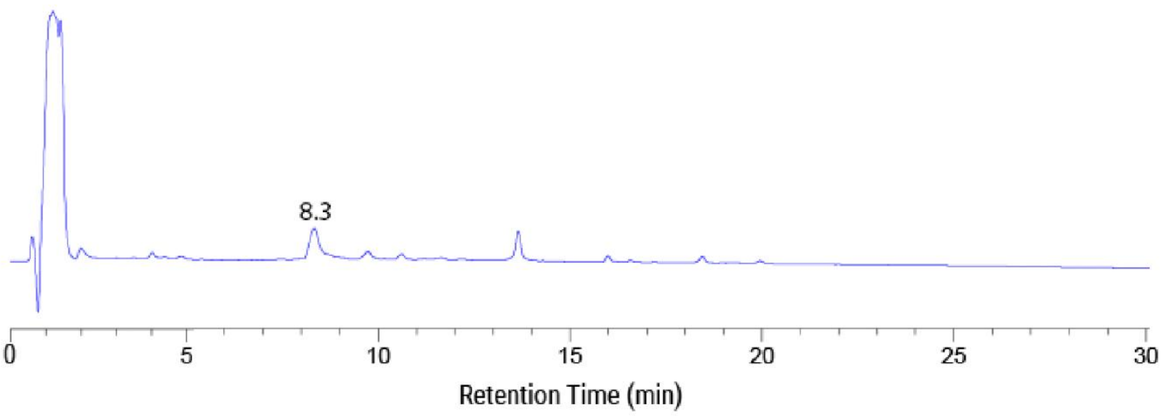

HRMS Trace of Peak at 8.3 min

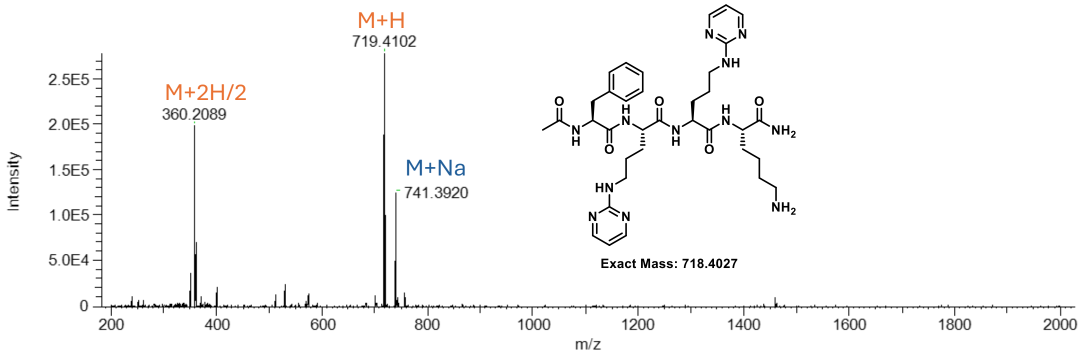

Ac-FRRK-CONH$_2$ product peptide 2c. LCMS for $C_{35}H_{51}N_{12}O_5$: *m/z* 719.4102 (calcd $[M+H]^+$ = 719.4100), *m/z* 360.2089 (calcd $[M+2H]/2^+$ = 360.2086), *m/z* 741.3920 (calcd $[M+Na]^+$ = 741.3919) (HPLC analysis at 220 nm). Retention time in HPLC: 8.3 min.

For Peptide **1d:**

HPLC Trace of Ac-WARHD-CONH₂ Starting Peptide 1d at 220 nm

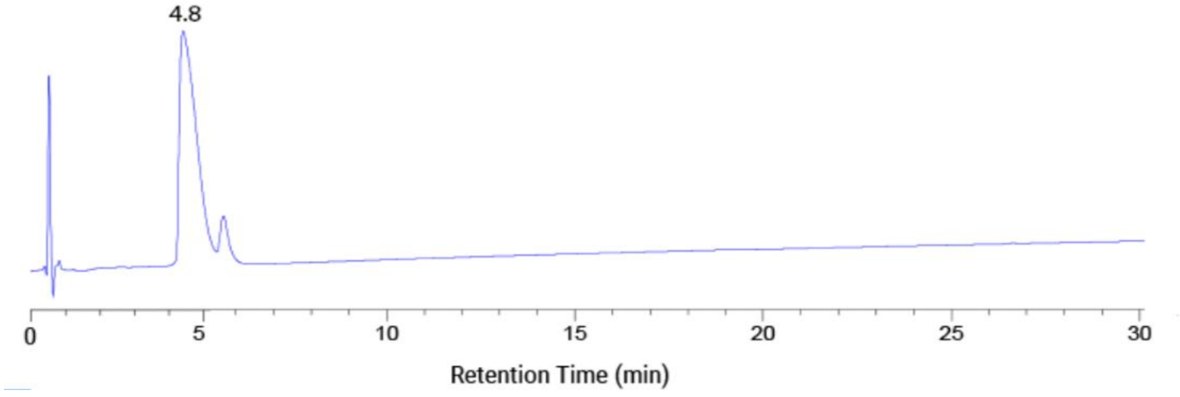

HRMS Trace of Peak at 4.8 min

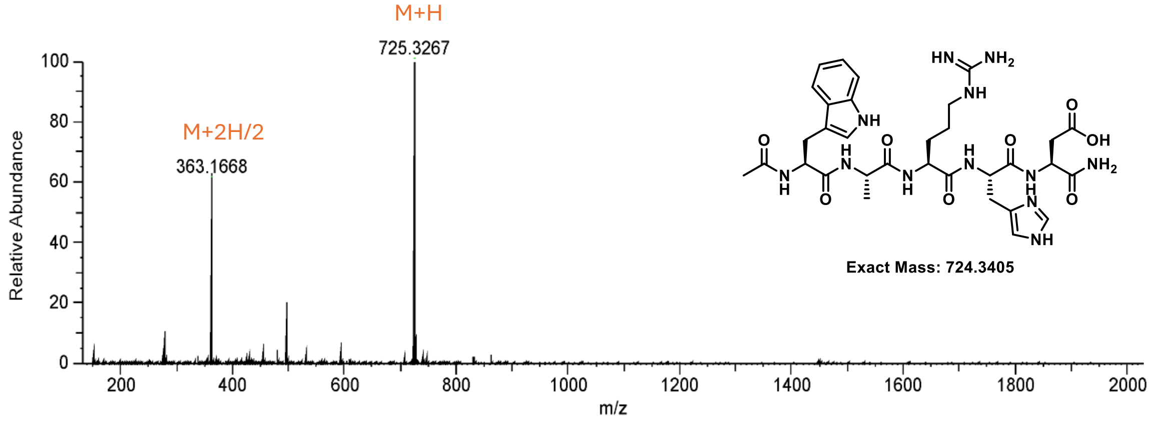

Ac-WARHD-CONH$_2$ starting peptide 1d. LCMS for $C_{32}H_{44}N_{12}O_8$: *m/z* 725.3267 (calcd $[M+H]^+$ = 725.3478), *m/z* 363.1668 (calcd $[M+2H]/2^+$ = 363.1775) (HPLC analysis at 220 nm). Retention time in HPLC: 4.8 min

HPLC Trace Ac-WARHD-CONH$_2$ Reaction Mixture at 220 nm

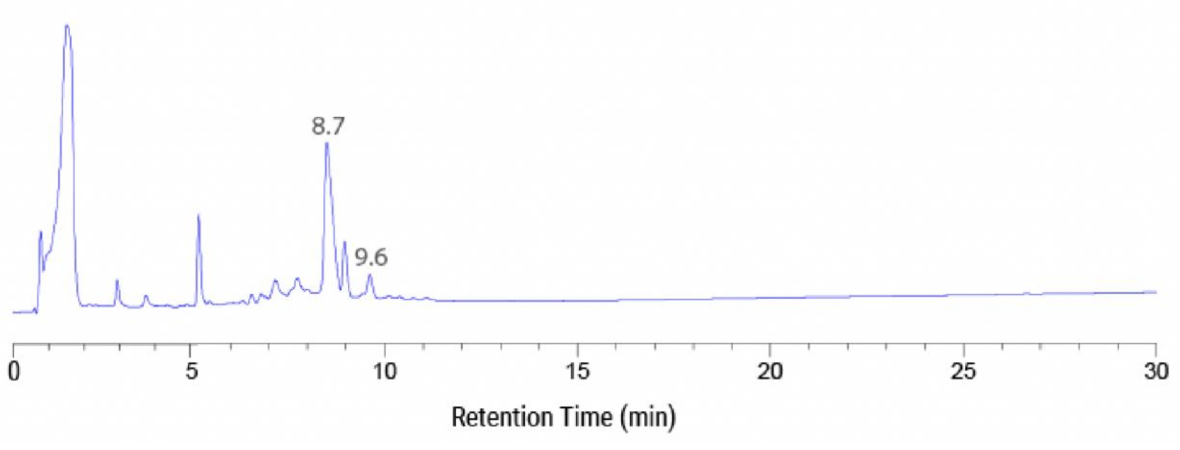

HRMS Trace of Peak at 8.7 min

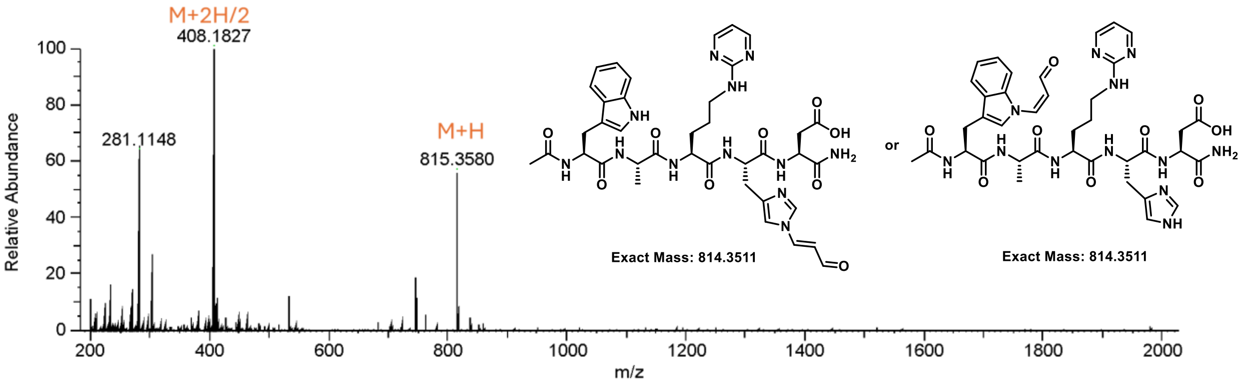

Ac-WARHD-CONH$_2$ intermediate peptide 2d'. LCMS for $C_{38}H_{47}N_{12}O_9$: *m/z* 815.3580 (calcd $[M+H]^+$ = 815.3583), *m/z* 408.1827 (calcd $[M+2H]/2^+$ = 408.1828) (HPLC analysis at 220 nm). Retention time in HPLC: 8.7 min.

HRMS Trace of Peak at 9.6 min

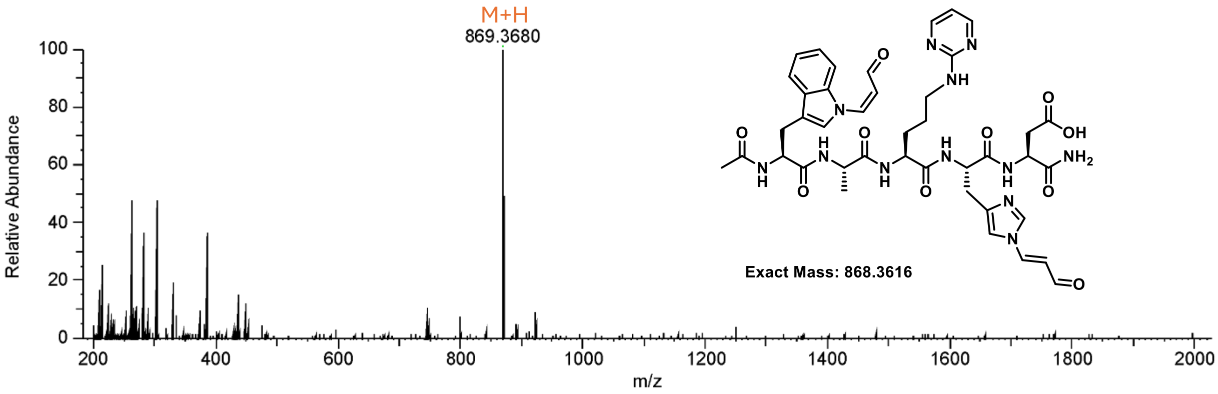

Ac-WARHD-CONH$_2$ intermediate peptide 2d". LCMS for $C_{41}H_{49}N_{12}O_{10}$: *m/z* 869.3680 (calcd $[M+H]^+$ = 869.3689) (HPLC analysis at 220 nm). Retention time in HPLC: 9.6 min

HPLC Trace of Ac-WARHD-CONH$_2$ Reaction Mixture After the Addition of BuNH$_2$ at 220 nm

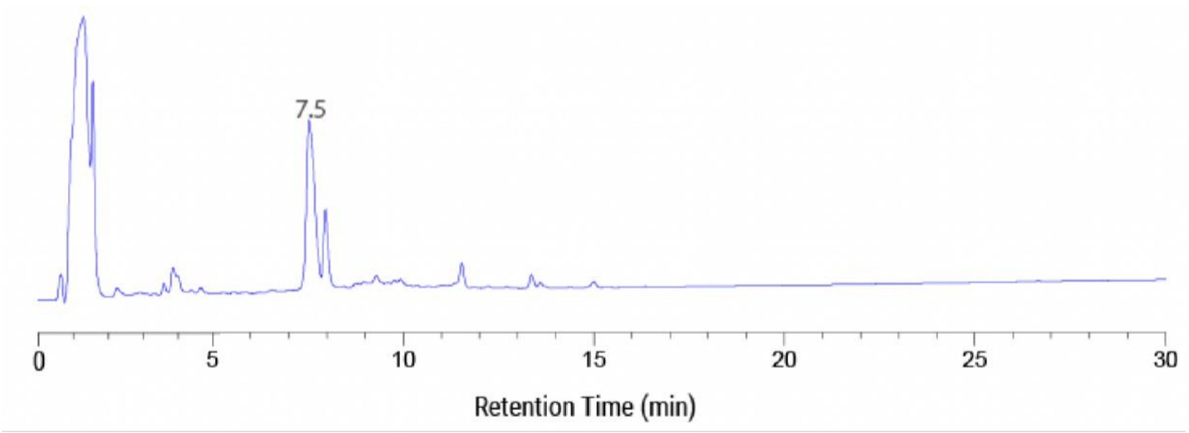

HRMS Trace of Peak at 7.5 min

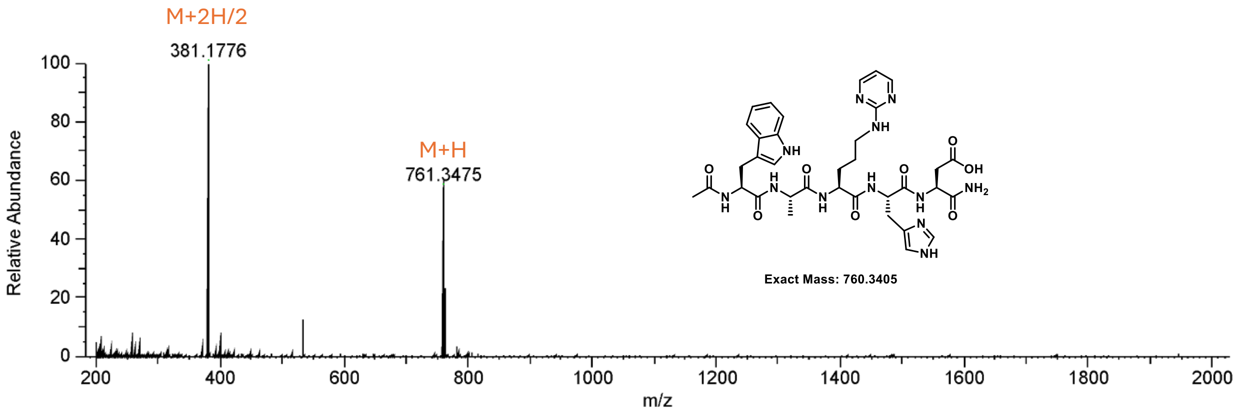

Ac-WARHD-CONH$_2$ product peptide 2d. LCMS for $C_{35}H_{45}N_{12}O_8$: *m/z* 761.3475 (calcd $[M+H]^+$ = 761.3478), *m/z* 381.1776 (calcd $[M+2H]/2^+$ = 381.1775) (HPLC analysis at 220 nm). Retention time in HPLC: 7.5 min.

For Peptide **1e**:

HPLC Trace of Ac-HYF-CONH$_2$ Starting Peptide 1e at 220 nm

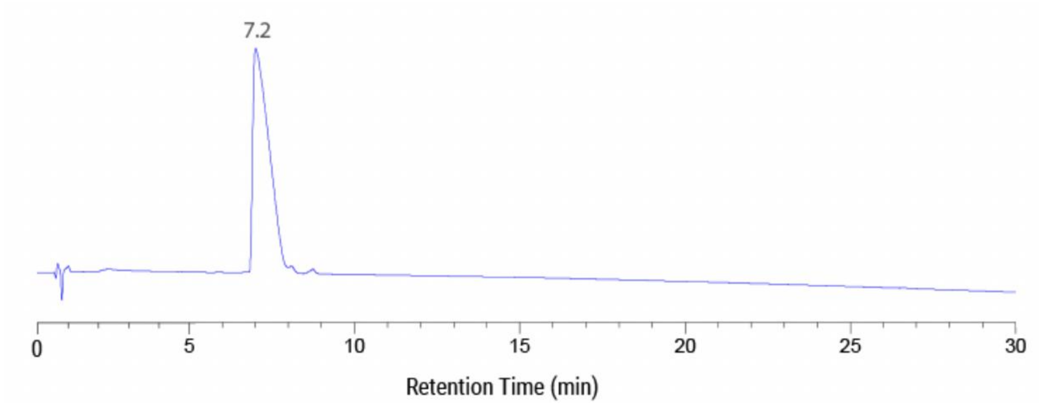

HRMS Trace of Peak at 7.2 min

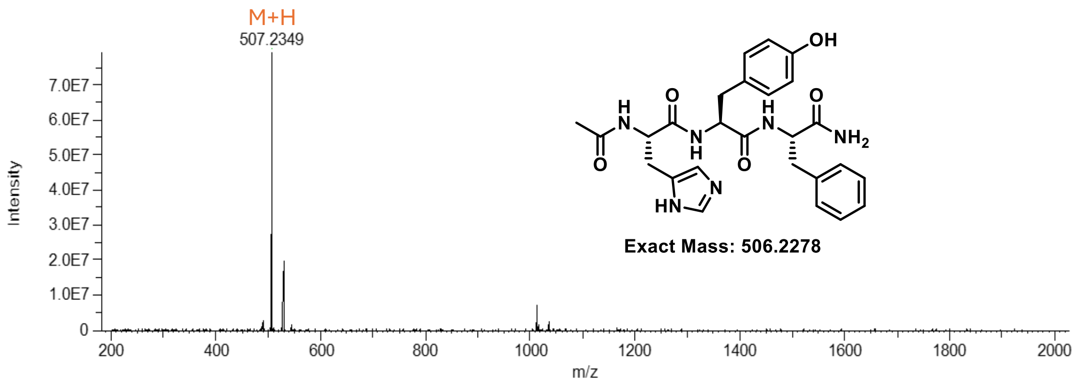

Ac-HYF-CONH$_2$ starting peptide 1e. LCMS for $C_{28}H_{31}N_6O_5$: *m/z* 507.2349 (calcd $[M+H]^+$ = 507.2350) (HPLC analysis at 220 nm). Retention time in HPLC: 7.2 min.

HPLC Trace of Ac-HYF-CONH$_2$ Reaction Mixture at 220 nm

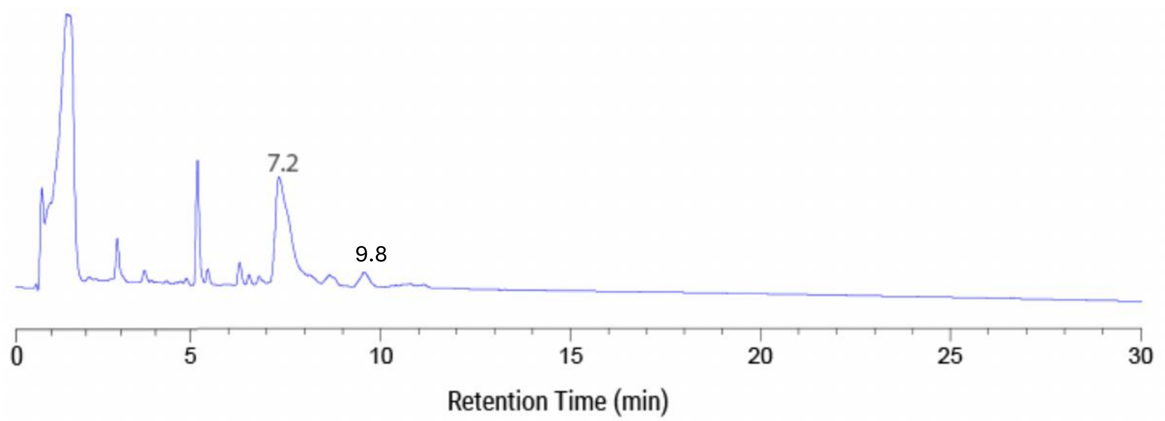

HRMS Trace of Peak at 9.8 min

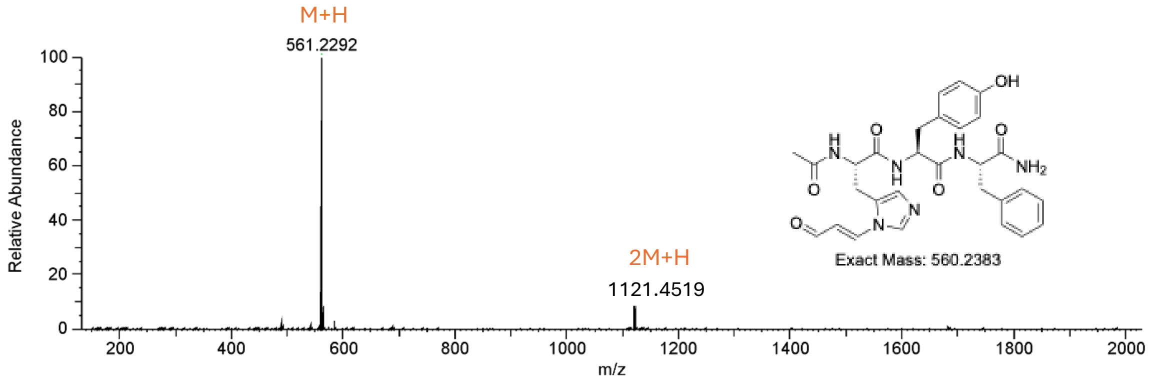

Ac-HYF-CONH$_2$ intermediate peptide 1e. LCMS for $C_{31}H_{33}N_6O_6$: *m/z* 561.2292 (calcd $[M+H]^+$ = 561.2383) LCMS: *m/z* 1121.4519 (calcd $[2M+H]^+$ = 1121.4766) (HPLC analysis at 220 nm). Retention time in HPLC: 9.8 min

HPLC Trace of Ac-HYF-CONH$_2$ Reaction Mixture After the addition of BuNH$_2$ at 220 nm

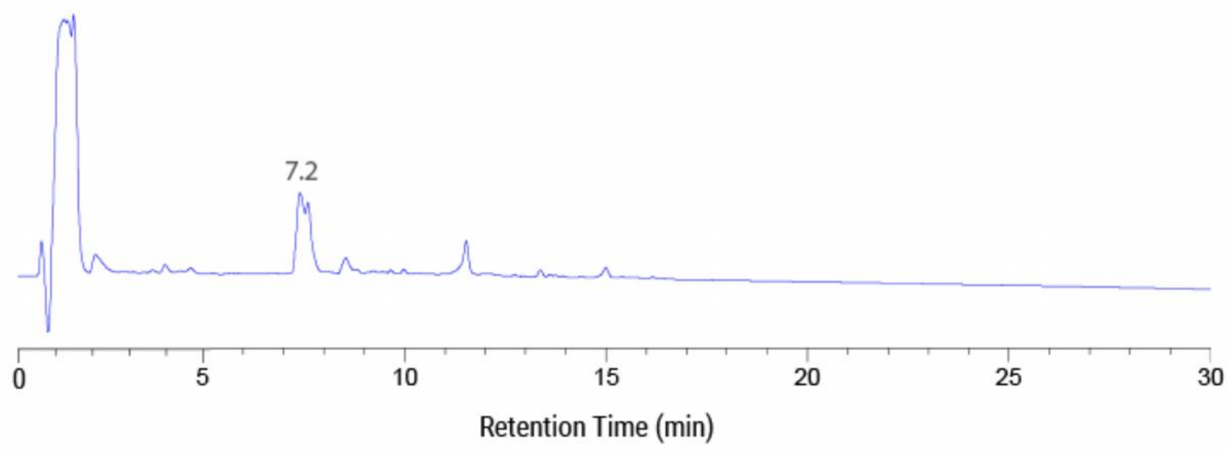

HRMS Trace of Peak at 7.2 min

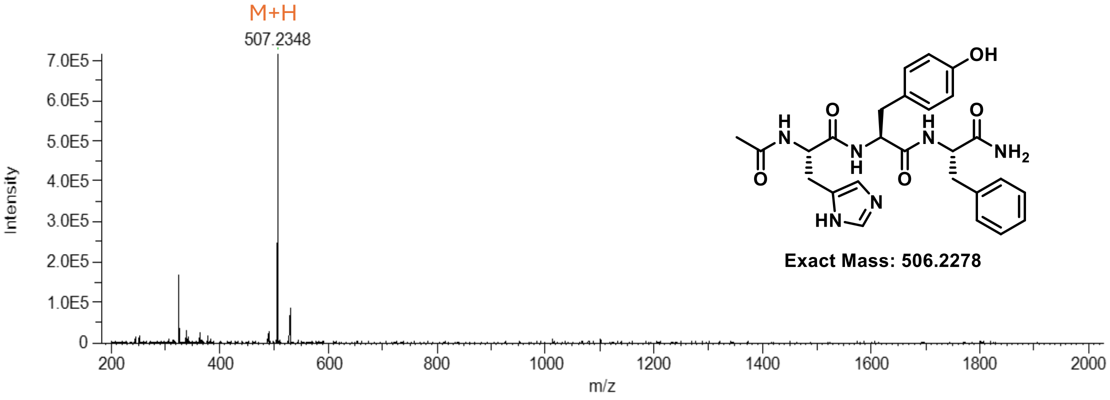

Ac-HYF-CONH$_2$ resulting peptide 1e. LCMS for $C_{28}H_{31}N_6O_5$: *m/z* 507.2348 (calcd $[M+H]^+$ = 507.2350) (HPLC analysis at 220 nm). Retention time in HPLC: 7.2 min.

Reactivity of Cysteine with MDA: To test the reactivity of cysteine towards MDA, reaction of Fmoc-Cys-OH was carried out with MDA under optimized reaction conditions. It was found that, under these conditions, cysteine does not show any reactivity towards MDA.

HPLC trace of Fmoc-Cys-OH at 220 nm.

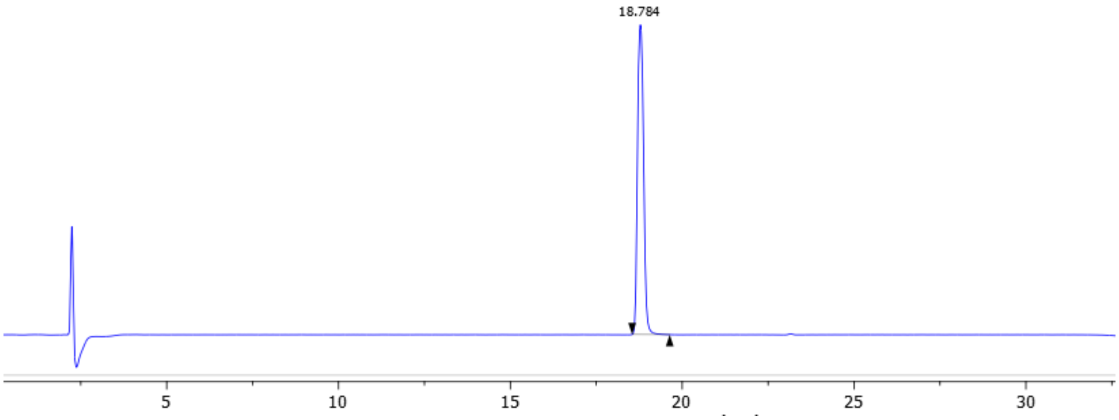

HPLC trace of Fmoc-Cys-OH reaction mixture with MDA at 220 nm.

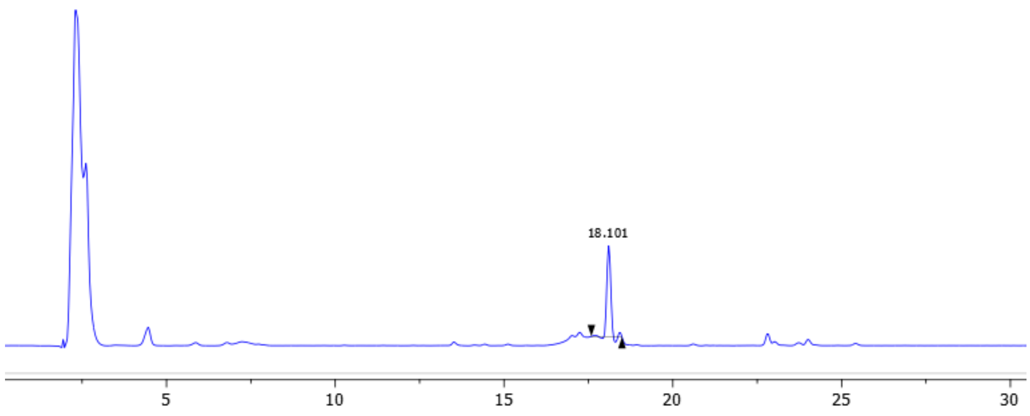

XII. Substrate scope of Modification of arginine containing peptides 1f-1k with MDA

Reactions of peptides **1f-1k** were carried out using **general procedure A**. In a one-dram vial equipped with a magnetic stirrer, the starting peptide (**1f-1k**) (0.002 mmol, 1 equiv), malondialdehyde (MDA) (0.2 mmol, 100 equiv) was added. Followed by 500 µL of 12M HCl. The reaction was allowed to proceed for 1 hour at room temperature then diluted to 2 mL with buffer (pH 7). Subsequently, the reaction mixture was injected into the HPLC to determine the % conversion of peptide **1f-1k** to the labeled peptides **2f-2k** and their mass confirmed with LC-MS. HPLC analysis was carried out utilizing **HPLC Method A** at detection wavelength 220 nm. Refer to optimization table below for reaction conditions. The masses of the products were confirmed with LC-MS.

For Peptide 1f:

HPLC Trace of Peptide of Ac-ARYDSFN-CONH$_2$ Starting Peptide 1f at 220 nm

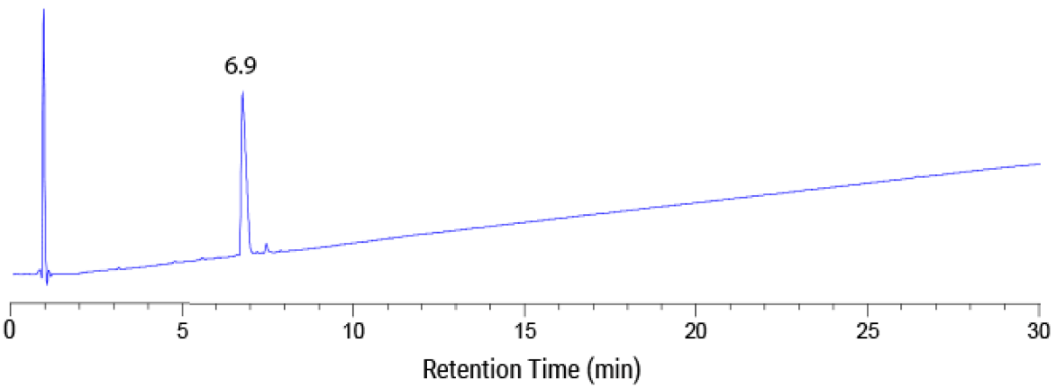

HRMS Trace of Peak at 6.9 min

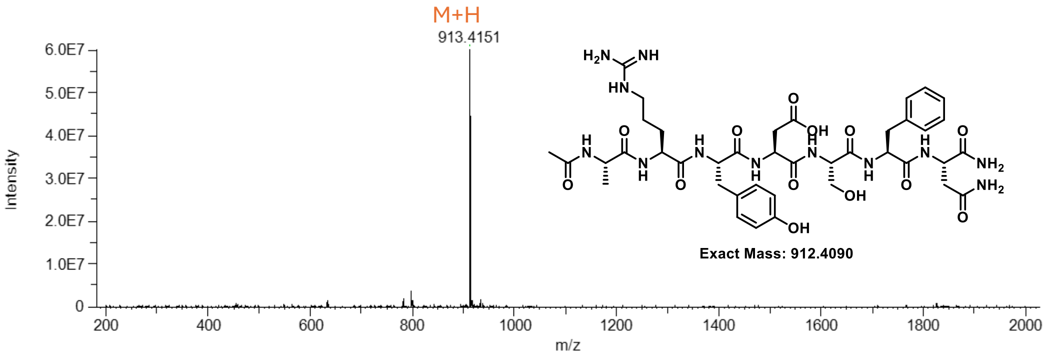

Ac-ARYDSFN-CONH$_2$ starting peptide 1f. LCMS for $C_{40}H_{57}N_{12}O_{13}$: *m/z* 913.4151 (calcd $[M+H]^+$ = 913.4163) (HPLC analysis at 220 nm). Retention time in HPLC: 6.9 min.

HPLC Trace of Peptide of Ac-ARYDSFN-CONH$_2$ Reaction Mixture at 220 nm

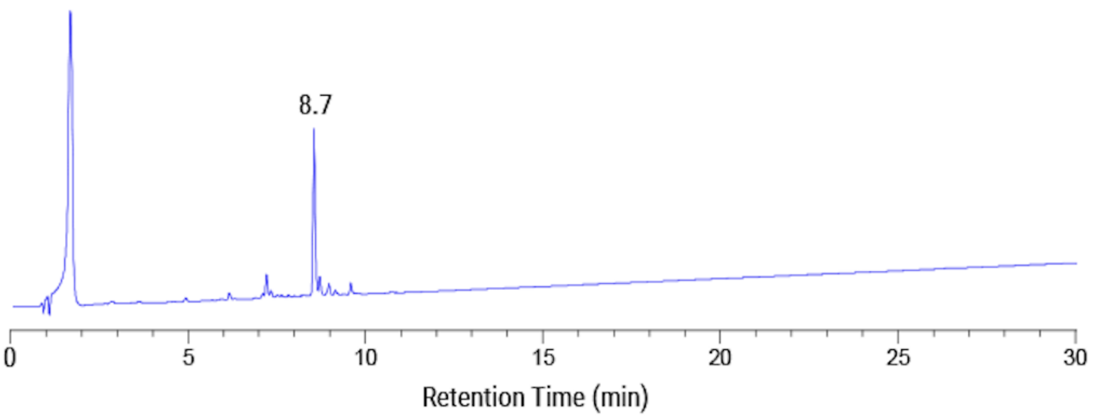

HRMS Trace of Peak at 8.7 min

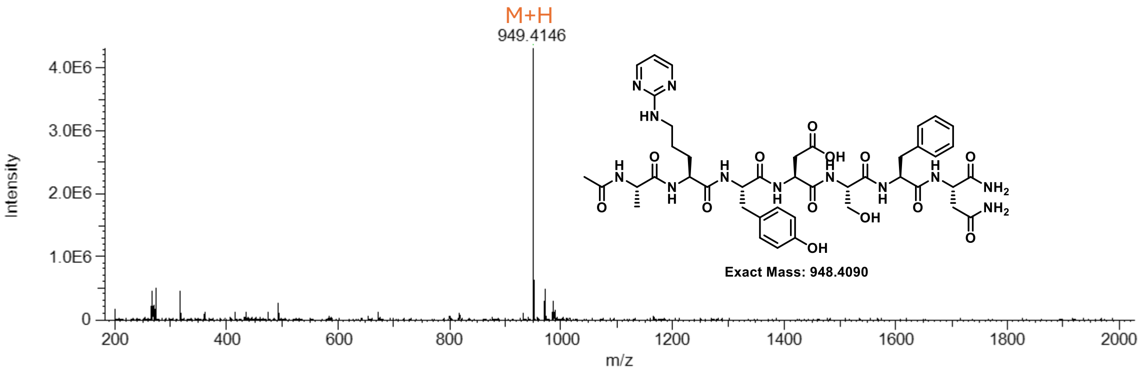

Ac-ARYDSFN-CONH$_2$ resulting peptide 2f. LCMS for $C_{43}H_{57}N_{12}O_{13}$: *m/z* 949.4146 (calcd $[M+H]^+$ = 949.4163) (HPLC analysis at 220 nm). Retention time in HPLC: 8.7 min.

For Peptide 1g:

HPLC Trace of Peptide of Ac-FADSRY-CONH₂ Starting Peptide 1g at 220 nm

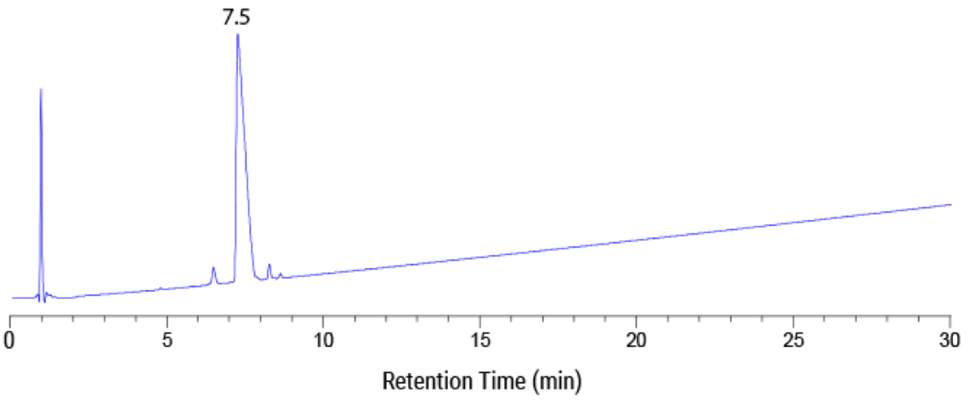

HRMS Trace of Peak at 7.5 min

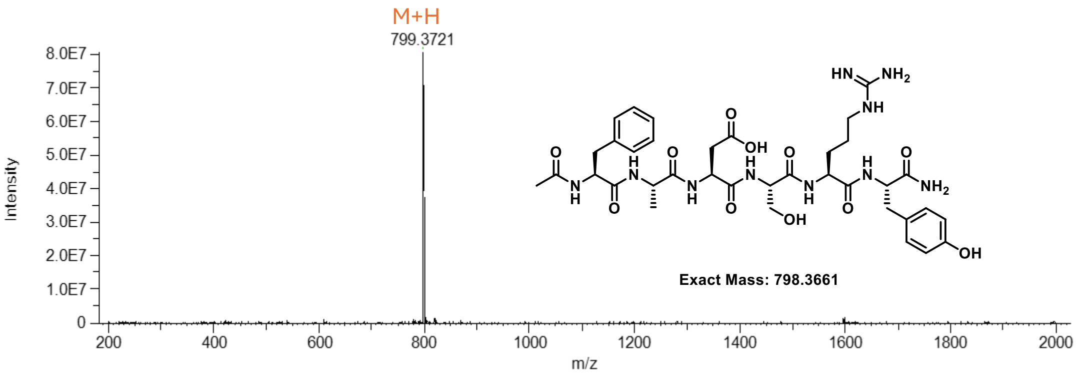

Ac-FADSRY-CONH₂ starting peptide 1g. LCMS for $C_{36}H_{51}N_{10}O_{11}$: *m/z* 799.3721 (calcd $[M+H]^+$ = 799.3733) (HPLC analysis at 220 nm). Retention time in HPLC: 7.5 min

HPLC Trace of Peptide of Ac-FADSRY-CONH₂ Reaction Mixture at 220 nm

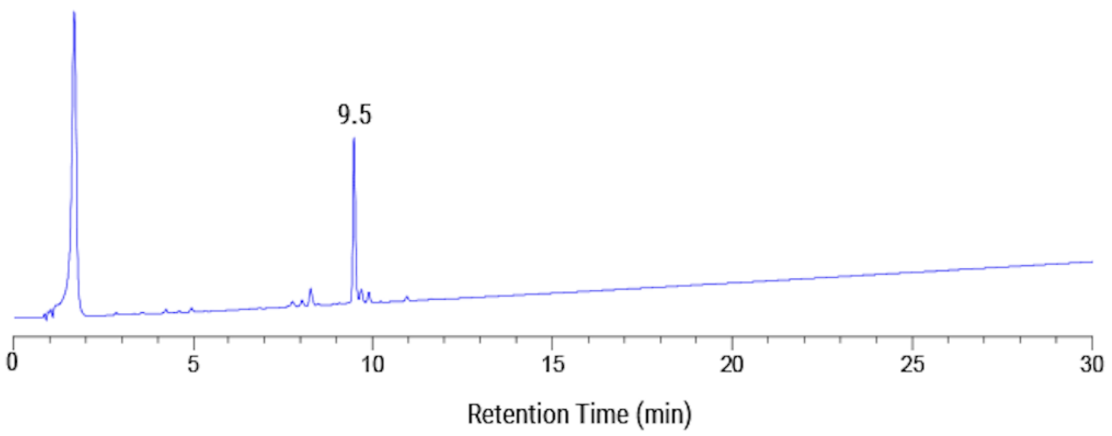

HRMS Trace of Peak at 9.5 min

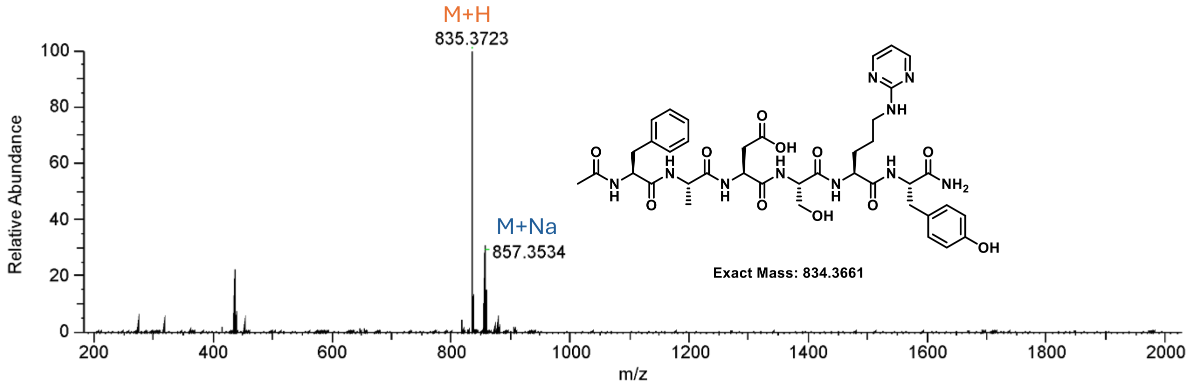

Ac-FADSRY-CONH$_2$ resulting peptide 2g. LCMS for $C_{39}H_{50}N_{10}O_{11}$: *m/z* 835.3723 (calcd $[M+H]^+$ = 835.3733), *m/z* 857.3534 (calcd $[M+Na]^+$ = 857.3553) (HPLC analysis at 220 nm). Retention time in HPLC: 9.5 min.

For Peptide 1h:

HPLC Trace of Peptide of Ac-VERMQYF-CONH$_2$ Starting Peptide 1h at 220 nm

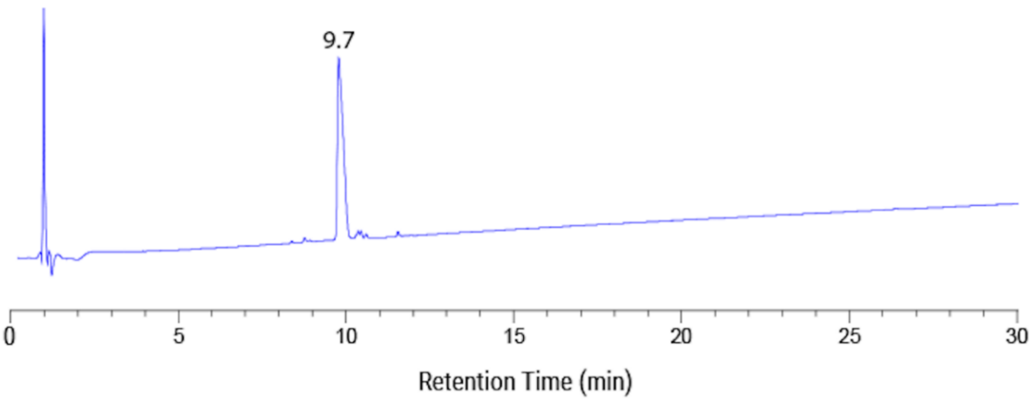

HRMS Trace of Peak at 9.7 min

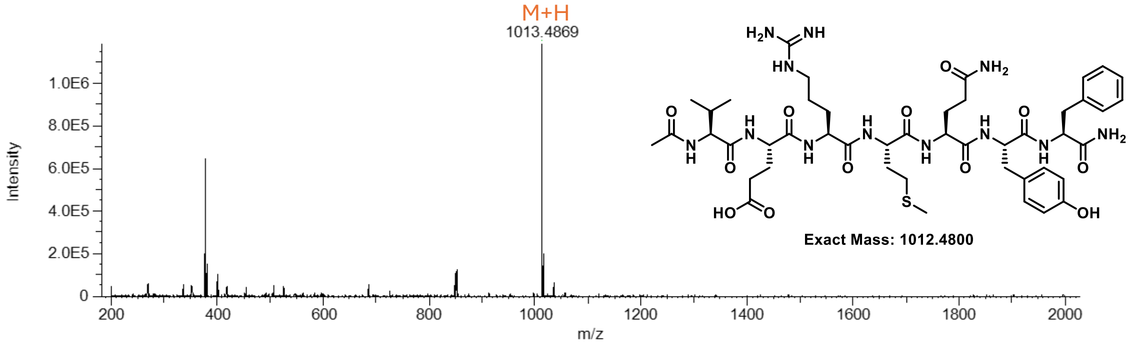

Ac-VERMQYF-CONH$_2$ starting peptide 1h. LCMS for $C_{46}H_{69}N_{12}O_{12}S$: *m/z* 1013.4869 (calcd $[M+H]^+$ = 1013.4873) (HPLC analysis at 220 nm). Retention time in HPLC: 9.7 min.

HPLC Trace of Peptide of Ac-VERMQYF-CONH$_2$ Reaction Mixture at 220 nm

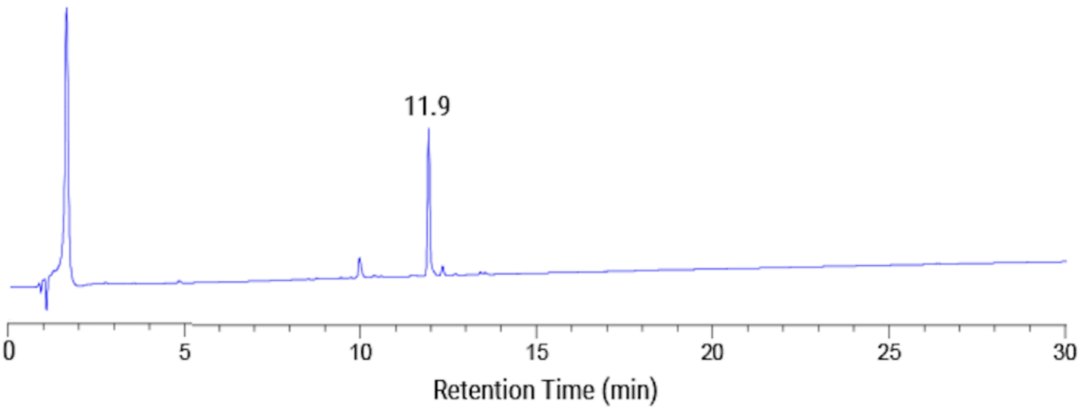

HRMS Trace of Peak at 11.9 min

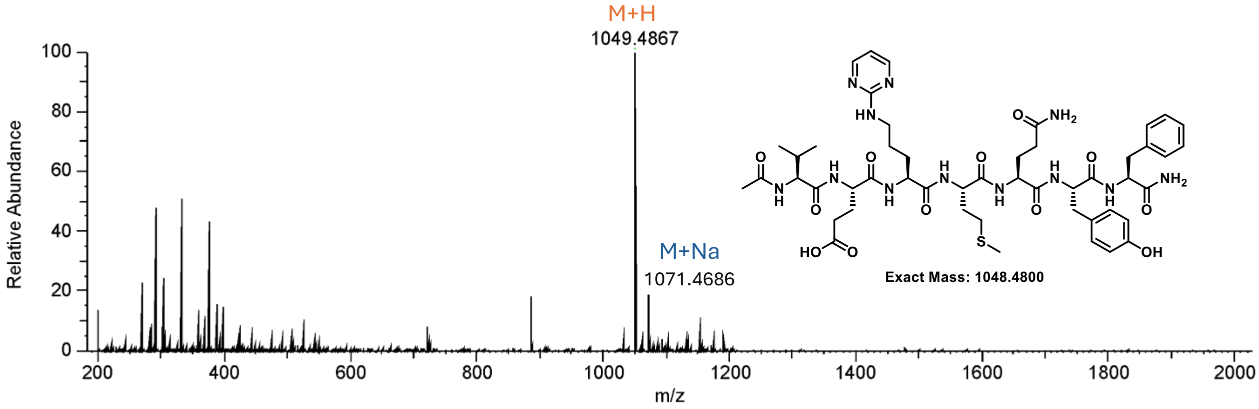

Ac-VERMQYF-CONH$_2$ resulting peptide 2h. LCMS for $C_{49}H_{69}N_{12}O_{12}S$: *m/z* 1049.4867 (calcd $[M+H]^+$ = 1049.4873) *m/z* 1071.4686 (calcd $[M+Na]^+$ = 1071.4693) (HPLC analysis at 220 nm). Retention time in HPLC: 11.9 min.

For Peptide 1i:

HPLC Trace of Peptide of Ac-RTYFL-CONH$_2$ Starting Peptide 1i at 220 nm

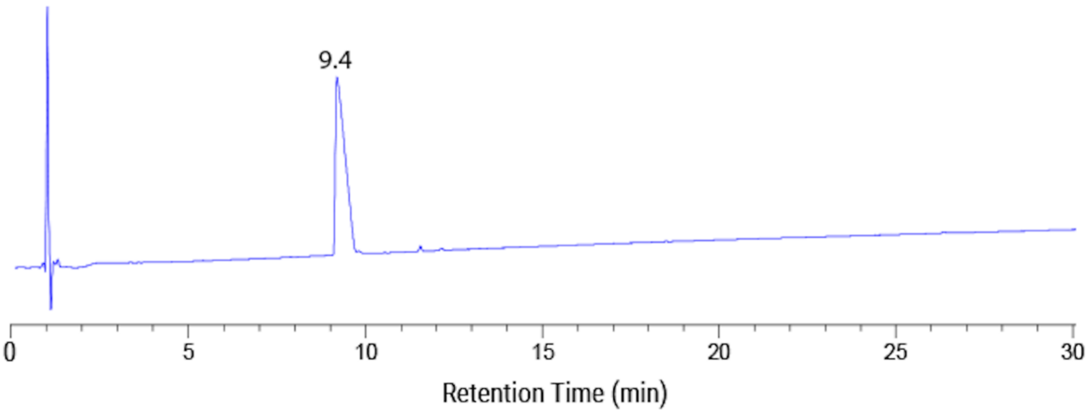

HRMS Trace of Peak at 9.4 min

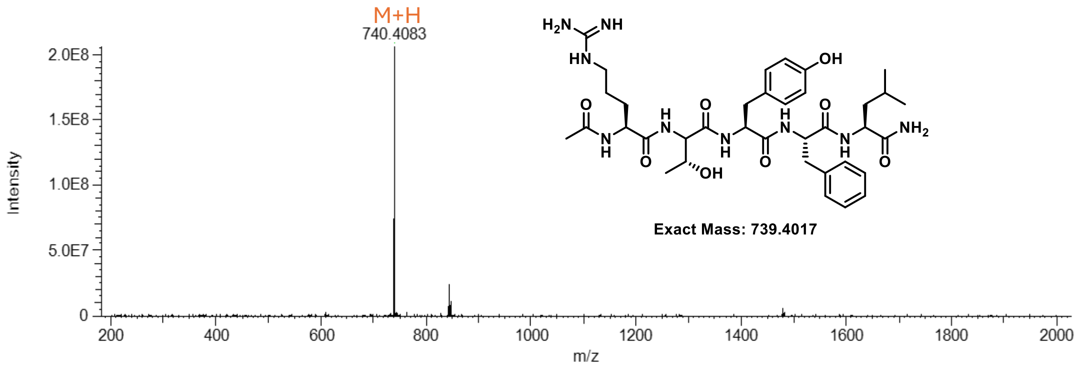

Ac-RTYFL-CONH$_2$ starting peptide 1i. LCMS for $C_{36}H_{54}N_9O_8$: *m/z* 740.4083 (calcd $[M+H]^+$ = 740.4090) (HPLC analysis at 220 nm). Retention time in HPLC: 9.4 min.

HPLC Trace of Peptide of Ac-RTYFL-CONH₂ Reaction Mixture at 220 nm

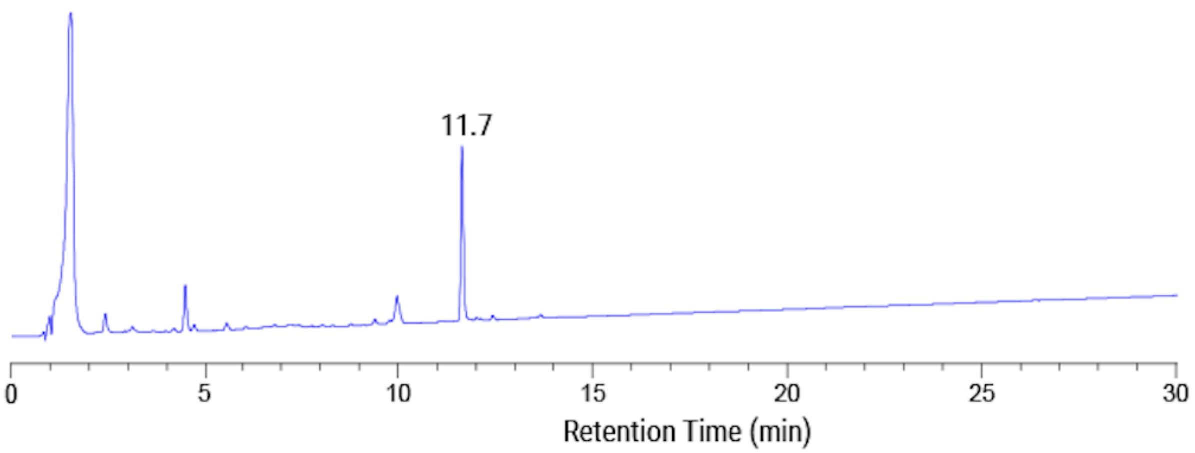

HRMS Trace of Peak at 11.7 min

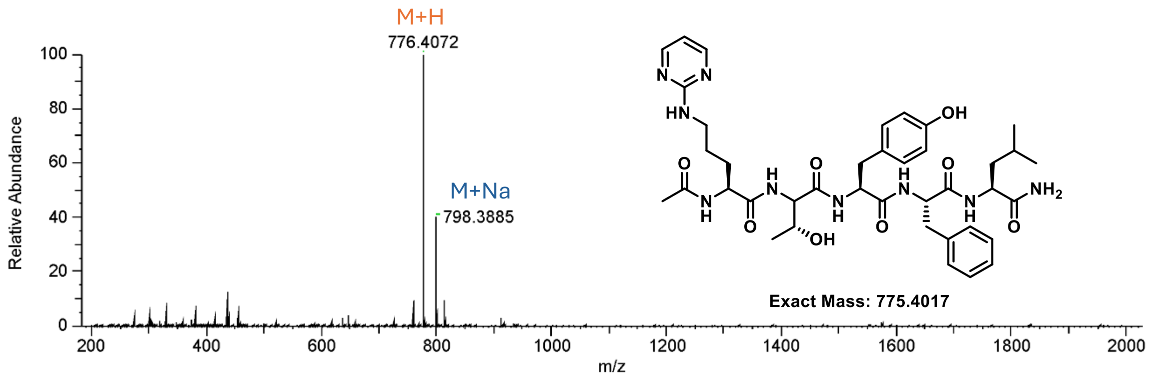

Ac-RTYFL-CONH$_2$ resulting peptide 2i. LCMS for $C_{39}H_{53}N_9O_8$: *m/z* 776.4089 (calcd $[M+H]^+$ = 776.4090) *m/z* 798.3885 (calcd $[M+Na]^+$ = 798.3909) (HPLC analysis at 220 nm). Retention time in HPLC: 11.7 min.

For Peptide 1j

HPLC Trace of Peptide of Ac-DFRNYP-CONH₂ Starting Peptide 2j at 220 nm

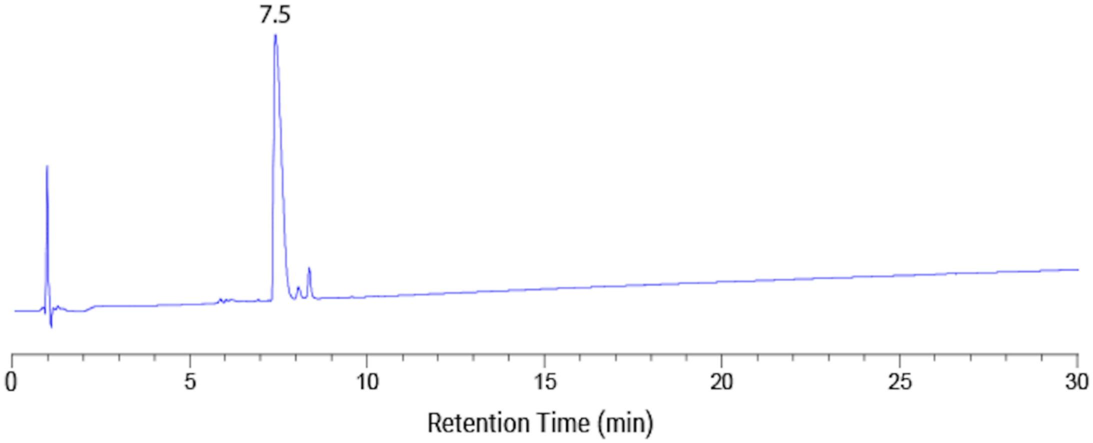

HRMS Trace of Peak at 7.5 min

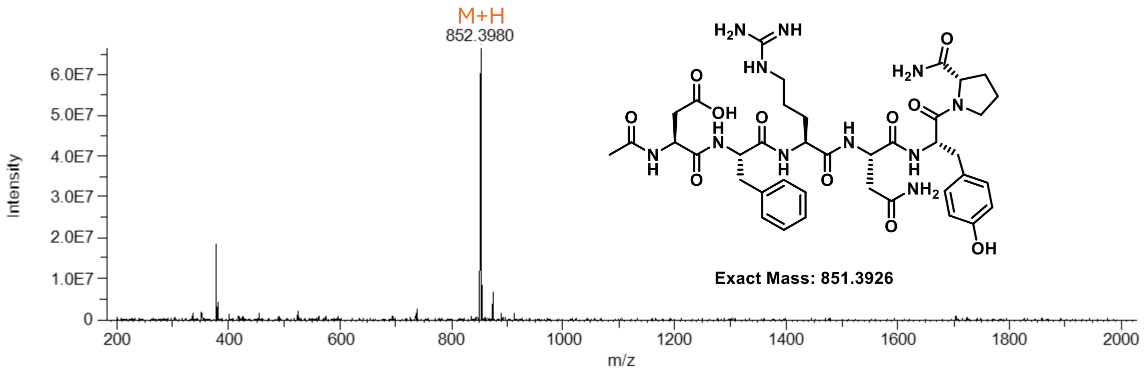

Ac-DFRNYP-CONH$_2$ starting peptide 1j. LCMS for $C_{39}H_{54}N_{11}O_{11}$: *m/z* 852.3980 (calcd $[M+H]^+$ = 852.3999) (HPLC analysis at 220 nm). Retention time in HPLC: 7.5 min.

HPLC Trace of Peptide of Ac-DFRNYP-CONH₂ Reaction Mixture at 220 nm

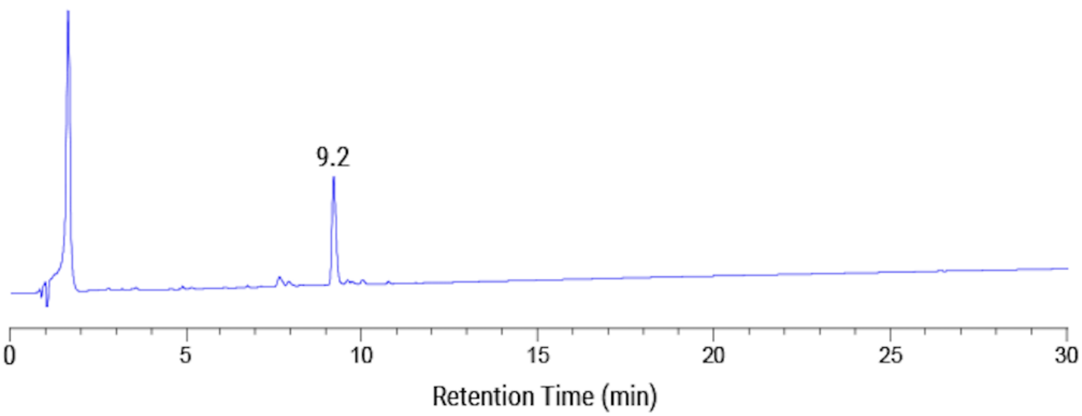

HRMS Trace of Peak at 9.2 min

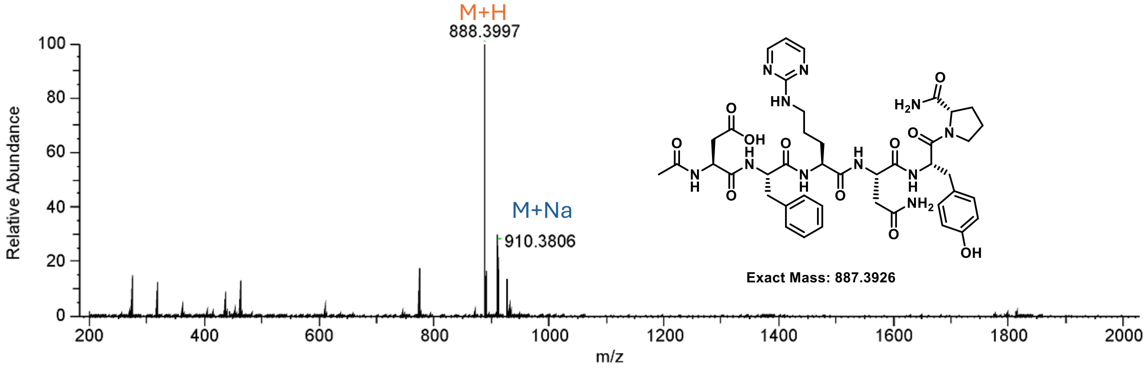

Ac-DFRNYP-CONH$_2$ resulting peptide 2j. LCMS for $C_{42}H_{53}N_{11}O_{11}$: *m/z* 888.3997 (calcd $[M+H]^+$ = 888.3999), *m/z* 910.3806 (calcd $[M+Na]^+$ = 910.3818) (HPLC analysis at 220 nm). Retention time in HPLC: 9.2 min.

For Peptide 1k:

HPLC Trace of Peptide of Ac-YREMYRF-CONH$_2$ Starting Peptide 2k at 220 nm

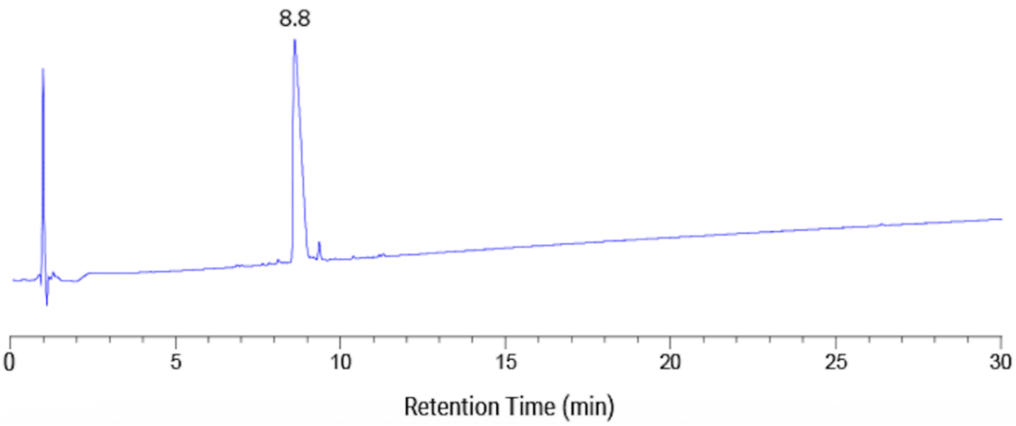

HRMS Trace of Peak at 8.8 min

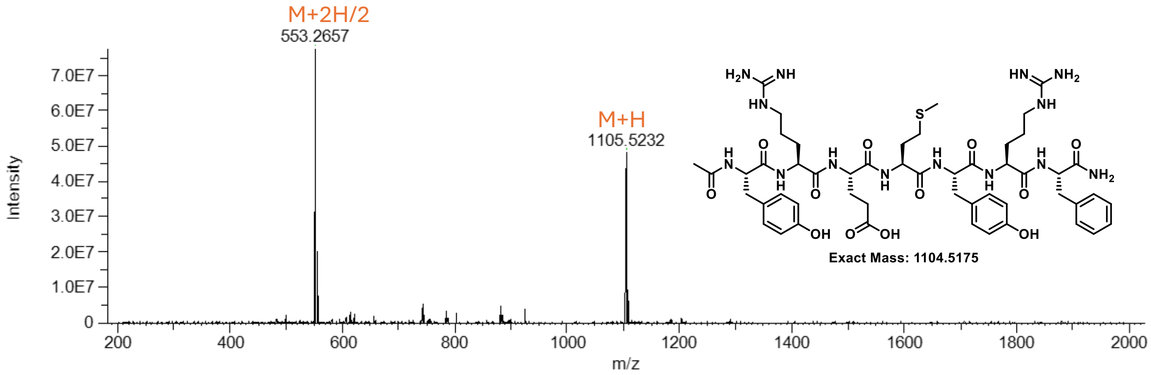

Ac-DFRNYP-CONH$_2$ starting peptide 1k. LCMS for $C_{51}H_{73}N_{14}O_{12}S$: *m/z* 1105.5232 (calcd $[M+H]^+$ = 1105.5248), *m/z* 553.2657 (calcd $[M+2H/2]^+$ = 553.2660) (HPLC analysis at 220 nm). Retention time in HPLC: 8.8 min.

HPLC Trace of Peptide of Ac-YREMYRF-CONH$_2$ Reaction Mixture at 220 nm

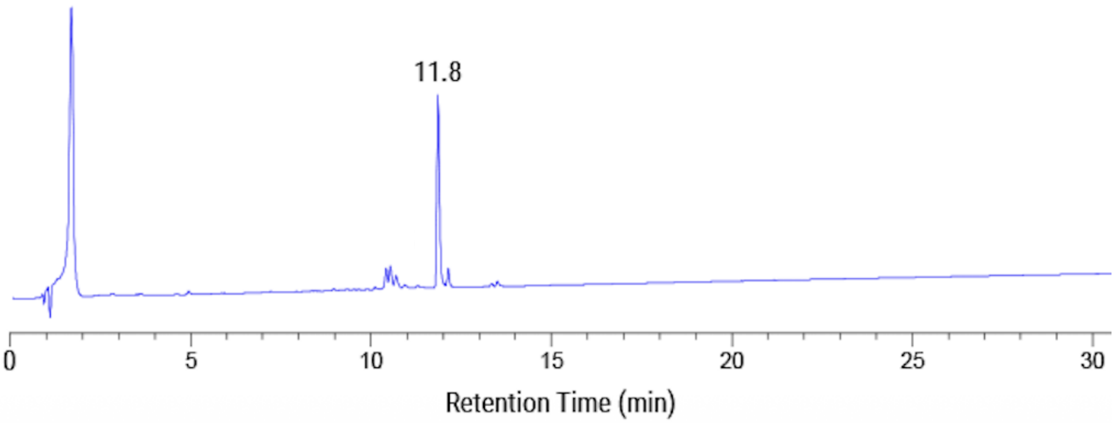

HRMS Trace of Peak at 11.8 min

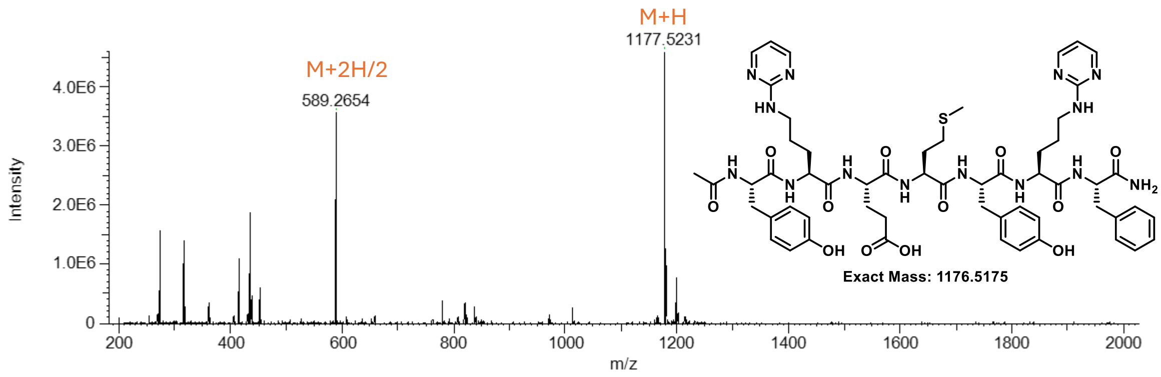

Ac-YREMYRF-CONH2 resulting peptide 2k. LCMS for $C_{57}H_{73}N_{14}O_{12}S$: *m/z* 1177.5231 (calcd $[M+H]^+$ = 1177.5248), *m/z* 589.2654 (calcd $[M+2H/2]^+$ = 589.266) (HPLC analysis at 220 nm). Retention time in HPLC: 11.8 min.

XIII Synthesis of chloroalkane carboxylate: The chloroalkane-carboxylic acid (Ct-COOH) tag synthesis consists of four major steps.[4]

In a 100 mL round bottom flask equipped with a magnetic stirrer, 2.1 g (20 mmol) of 2-(2-aminoethoxy)ethanol and 4.36 g (20 mmol) of Boc_2O was dissolved in 50 mL of ethanol at 0°C. The reaction was stirred for 2h at room temperature, diluted with CH_2Cl_2 and washed with brine solution. The organic layer was dried using MgSO4 and solvent was removed via rotavap to obtain a colorless oil compound **A**. Compound **A** (3.3 g, 16 mmol) was dissolved in anhydrous DMF and placed under N_2. The solution was cooled to 0°C, and 0.88 g of 60% NaH mineral oil was added. After stirring for 30 min, 6.1 g (24.7 mmol) of 1-chloro-6-iodohexane was added to the reaction mixture and was left to stir overnight. The reaction was quenched using 1M HCl and the resulting product was extracted using ethyl acetate and brine solution, then dried using MgSO4. The crude compound was purified by column chromatography using a gradient of 20:80 ethyl acetate/hexanes to 50:50 ethyl acetate/hexanes. Solvents were removed via rotavap to obtain a yellow oil **B**. Compound **B** was dissolved in 40 mL dry CH_2Cl_2 and 10 mL TFA was added to the solution at 0°C, then mixed for 2 h at room temperature. The solvent, TFA, and Terbutyloxycarbonyl was removed via rotavap to obtain compound **C**. Approximately 1 g of compound **C** (4.47 mmol) was dissolved in CH2Cl2 and placed under N_2. N,N-Diisopropylethyamine (1.35 mL), 4-dimethylaminopyridine (476 mg), and Succinic anhydride (800 mg, 8.0 mmol) were added to the reaction and stirred for 2h at room temperature under N_2. The reaction was quenched with 1 M HCl, and then extracted with CH2Cl2. The organic layer was dried using $MgSO_4$ and solvent was removed *via* rotavapor. The final product **D** was purified using HPLC Gradient B and analyzed via HRMS.

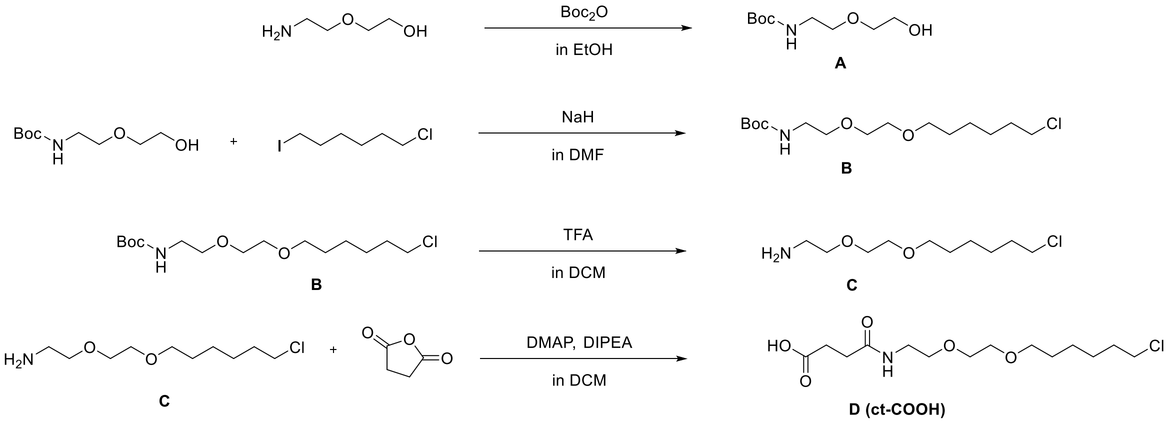

Synthesis of Chloroalkane-Tetramethyl rhodamine. The preparation of chloroalkane-tetramethylrhodamine (ct-TMR) begins with the dissolution of dry 5/6TAMRA-succinimidyl ester (100 µmol) in 2 mL molecular sieve-dried DMF. The reaction mixture is supplemented with 5 equivalents of diisopropylethylamine followed by 2 equivalents of chloroalkaneamine compound **C**. Following overnight incubation at room temperature with continuous agitation, the mixture is diluted with 3 mL water. Product purification is achieved through reverse-phase HPLC using a preparatory C18 column, employing a

gradient of 5-100% solvent B over 20 minutes, where solvent A consists of water with 0.1% trifluoroacetic acid and solvent B comprises acetonitrile with 0.1% trifluoroacetic acid.

XIV Synthesis of Chloroalkane Tag Peptides ct-1l & 1m and ct-2l & 2m: The synthesis of these peptides is carried out *via* on-resin amide bond formation. For peptide modification with an N-terminal chloroalkane tag, the coupling is performed while the peptide remains on the resin. The reaction mixture consists of 2.5 equivalents of chloroalkane carboxylic acid, 2.5 equivalents PyBOP, and 5 equivalents diisopropylethylamine in DMF, which is added to the N-terminally deprotected peptide on resin. The reaction proceeds at room temperature for 1 hour with agitation. Additional coupling cycles may be performed if incomplete reaction is observed. The final ct-peptide; **ct-1l and ct-1m** is cleaved using a mixture of 95% TFA, 2% H2O, 2% 1,2ethanedithiol, and 1% triisopropylsilane, followed by cold ether precipitation. For **ct-2l** and **ct-2m**, peptides **ct-1l** and **ct-1m** were modified with MDA (100 equiv) using optimized reaction conditions. Followed by addition of butyl amine to reverse the unwanted side reactivity of tryptophan. Subsequently, the reaction mixture was injected into the HPLC to determine the % conversion of peptide **ct-1l-ct-1m** to the labeled peptides **ct-2l-ct-2m** and their mass confirmed with LC-MS. HPLC analysis was carried out utilizing **HPLC Method A** at detection wavelength 220 nm.

HPLC Trace of Peptide of Ct-WRPVSI-CONH$_2$ Starting Peptide ct-1l at 220 nm

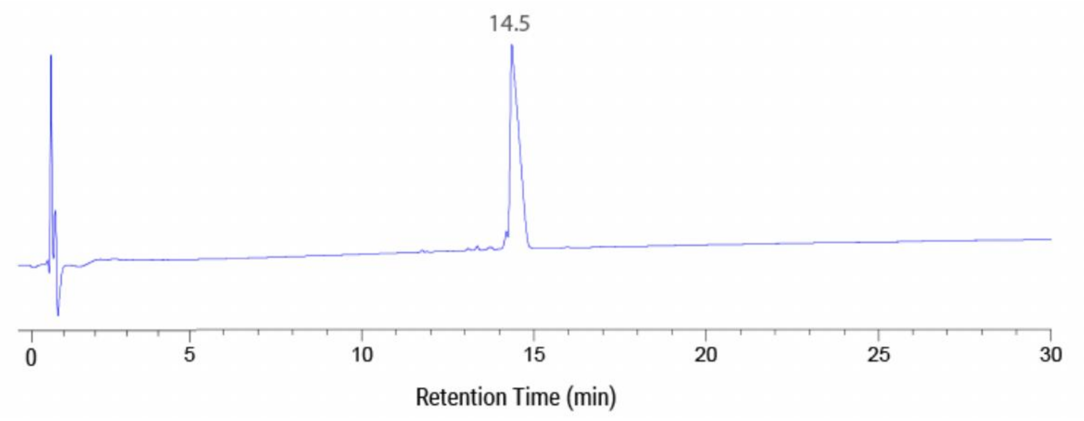

HRMS Trace of Peak at 14.5 min

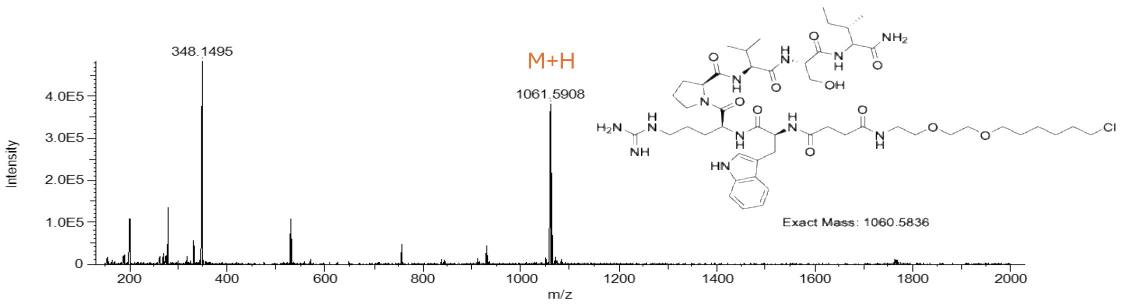

Ct-WRPVSI-CONH$_2$ starting peptide 1l. LCMS for $C_{50}H_{82}N_{12}O_{11}Cl$: *m/z* 1061.5908 (calcd $[M+H]^+$ = 1061.5909) (HPLC analysis at 220 nm). Retention time in HPLC: 14.5 min

HPLC Trace of Peptide of Ct-WRPVSI-CONH$_2$ Reaction Mixture at 220 nm

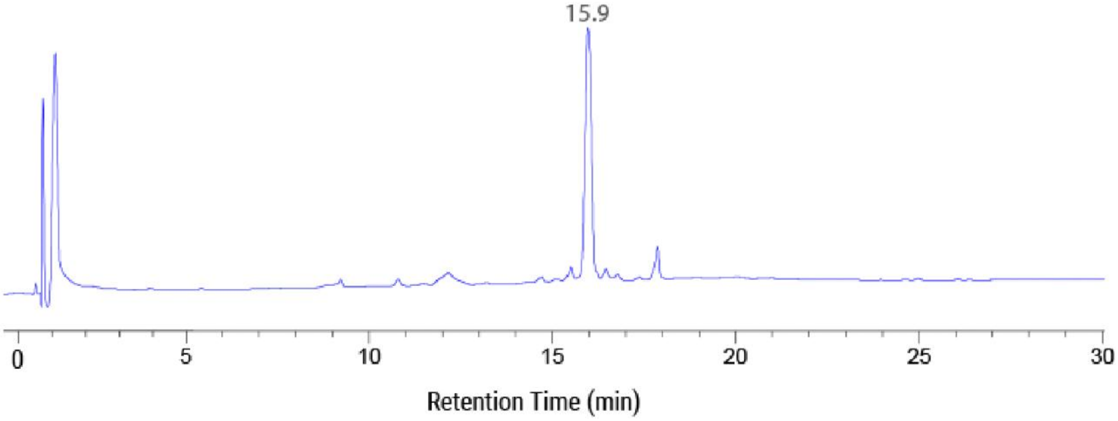

HRMS Trace of Peak at 15.9 min

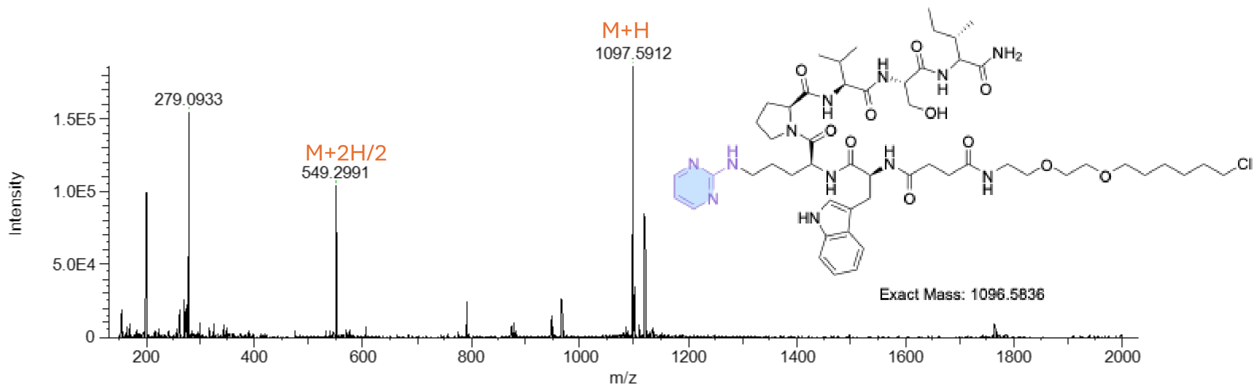

Ct-WRPVSI-CONH$_2$ resulting peptide 2l. LCMS for $C_{53}H_{82}N_{12}O_{11}Cl$: *m/z* 1097.5912 (calcd $[M+H]^+$ = 1097.5836), *m/z* 549.2291 (calcd $[M+2H/2]^+$ = 549.7956) (HPLC analysis at 220 nm). Retention time in HPLC: 15.9 min

HPLC Trace of Peptide of Ct-VGRAWNYA-CONH$_2$ Starting Peptide 1m at 220 nm

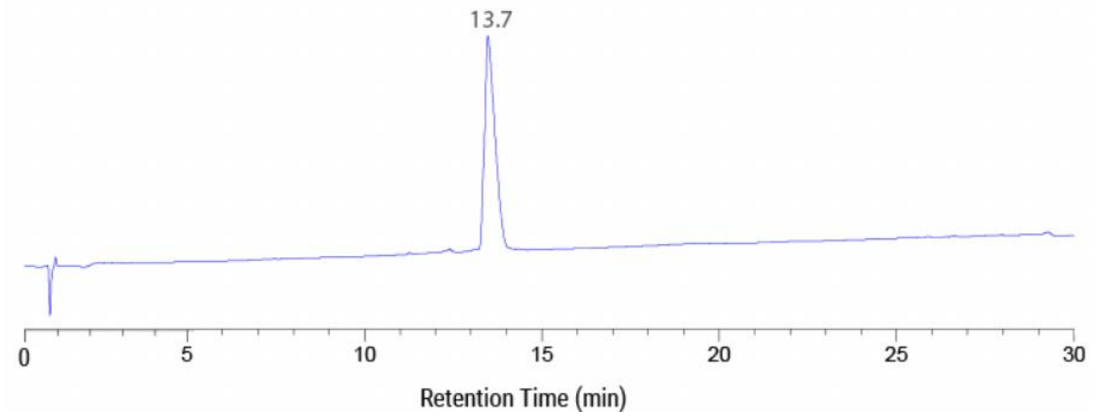

HRMS Trace of Peak at 13.7 min

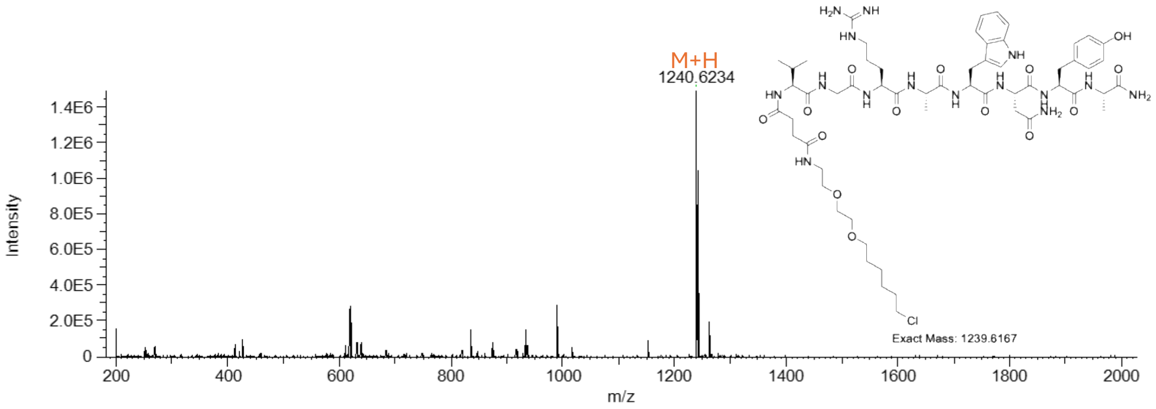

Ct-VGRAWNYA-CONH$_2$ starting peptide 1m. LCMS for $C_{57}H_{87}N_{15}O_{14}Cl$: *m/z* 1240.6234 (calcd $[M+H]^+$ = 1240.6167) (HPLC analysis at 220 nm). Retention time in HPLC: 13.7 min

HPLC Trace of Peptide of Ct-VGRAWNYA-CONH$_2$ Reaction Mixture at 220 nm

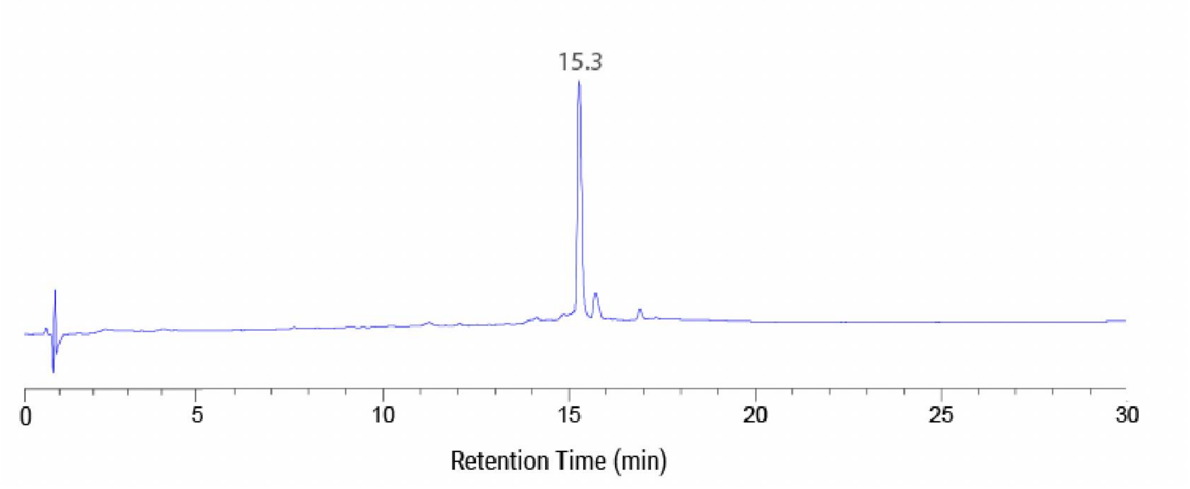

HRMS Trace of Peak at 15.3 min

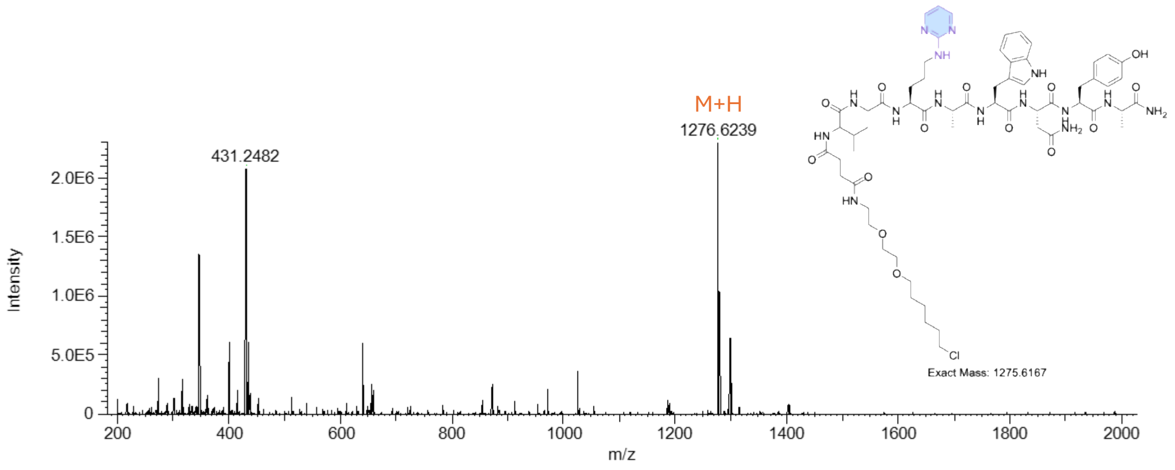

Ct-VGRAWNYA-CONH$_2$ resulting peptide 2m. LCMS for $C_{60}H_{87}N_{15}O_{14}Cl$: *m/z* 1276.6239 (calcd $[M+H]^+$ = 1276.6167) (HPLC analysis at 220 nm). Retention time in HPLC: 15.3 min

XV. Chloroalkane Permeability Assay

Experimental Protocol Design Control Implementation. The execution of CAPA requires careful consideration of several critical experimental design elements. Primary among these is the incorporation of appropriate controls to ensure reproducible and accurate data analysis. Each experimental plate must include both "no-molecule" and "no-dye" control wells to establish the maximum and minimum signals specific to the cellular conditions, format, and day of experimentation. No-molecule control wells receive only dilution buffer during the pulse step, thereby establishing the maximum red fluorescence baseline. This maximum signal is comparable to what would be observed with a ct-molecule displaying no appreciable cytosolic localization. Conversely, no-dye control wells receive plain media during the chase

step, establishing the minimum red fluorescence baseline attributed to cellular background fluorescence and any residual ct-dye remaining after wash steps.

For experimental plates containing numerous samples, particularly when high cell counts are desired or when working with reduced cell numbers per well, the extended time required for flow cytometry analysis of an entire 96-well plate may lead to reduced cell counts and increased cellular aggregation in later samples. To address this potential variation, control wells should be positioned both at the beginning and end of the plate. This arrangement enables verification of consistent fluorescence values across the entire analytical timeframe.

Small Molecule Controls and Quality Metrics: Beyond the basic controls, each experimental run incorporates a small molecule control to verify proper assay function. Any cell-penetrant ct-small molecule with accurately quantifiable concentration serves as an acceptable positive control, standard protocol employs chloroalkanetryptophan (ct-W) due to its straightforward synthesis and reliable quantitation via absorption at 280 nm. The inclusion of a complete dose-response experiment for ct-W on each plate serves multiple purposes: it validates proper assay function, ensures data comparability between independent trials, and provides reliable saturation values for curve fitting analyses.

The low-fluorescence saturation value, indicating high cytosolic localization, may exhibit slight variations depending on HaloTag expression levels and specific incubation durations. Therefore, the ct-W curve's low-fluorescence saturation value serves as the minimum value reference point for curve fitting analyses of all other ct-molecules tested on the same plate.

<u>Small molecule control standardization:</u>

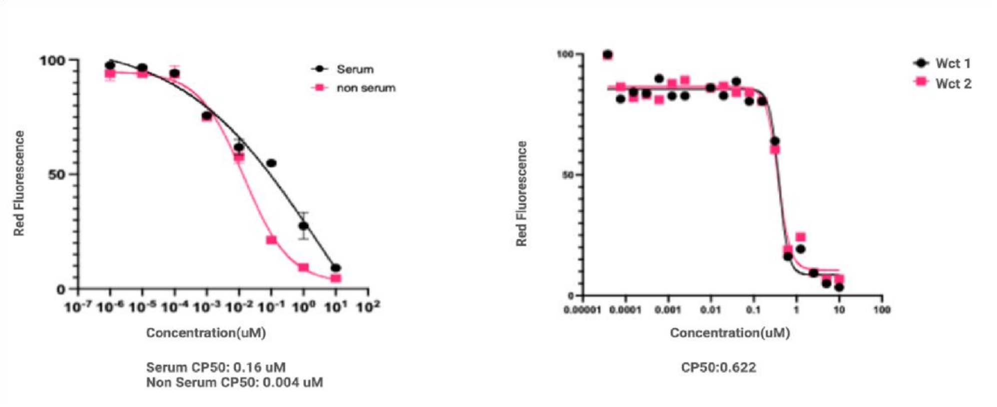

Data Analysis and Quality Control Population Gating and Signal Normalization: To ensure data quality, the analysis focuses exclusively on viable cells expressing HaloTag. The original construct by the Kritzer lab had GFP as a positive control. However, in absence of that, we gate the doublets. Take the highest 10% intensity of the cells as our data. Cases, where more than 60% of detection events are excluded may indicate problematic data resulting from unusually low HaloTag expression or compound toxicity. For each sample analysis, the mean red fluorescence of 5,000 cells undergoes normalization against the no-dye and no-molecule control values, representing background and maximum red fluorescence respectively.

Both mean and median values have proven suitable for representing individual data points, showing minimal differences in both raw and normalized values. The normalized fluorescence calculation follows the formula**: [(sample - no dye control) / (no molecule control - no dye control)] × 100%.**

Curve Fitting and Statistical Analysis: Dose-response relationships are visualized by plotting normalized red fluorescence values against ctmolecule concentration. The analysis employs a Hill equation curve fit with variable midpoint and slope parameters. The midpoint value, designated as CP50, represents the concentration at which 50% cytosolic penetration occurs under the given experimental conditions. The curve fitting equation incorporates the minimum value from the ct-W curve, the CP50 value, the curve fit slope, and the ctmolecule concentration. Maximum values for curve fitting derive from the empirically measured no-molecule control (100% normalized value). However, the minimum value utilizes the empirically measured saturation value observed at high concentrations of the small-molecule control (ct-W) rather than the no-dye control value. This adjustment accounts for the constitutive expression of HaloTag during dye incubation and wash steps, typically resulting in minimum values ranging from near-zero to 15%, with day-to-day variations. We use Prisim-Graphpad to plot the data and calculate the CP50 as stated above.

General workflow for CAPA assay

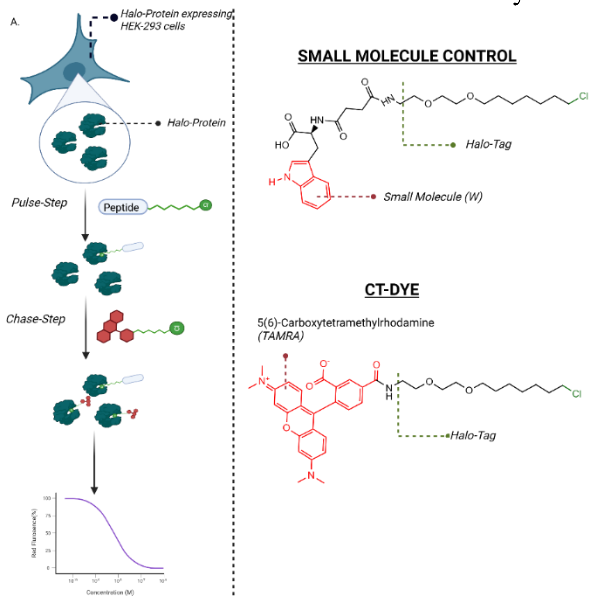

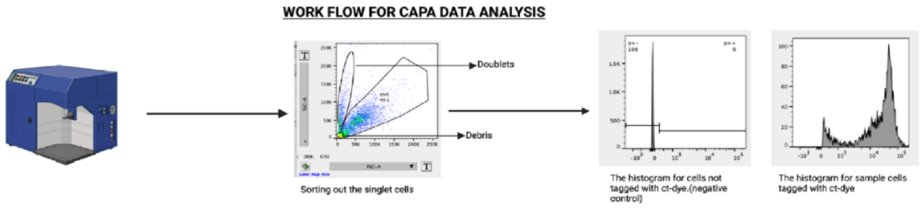

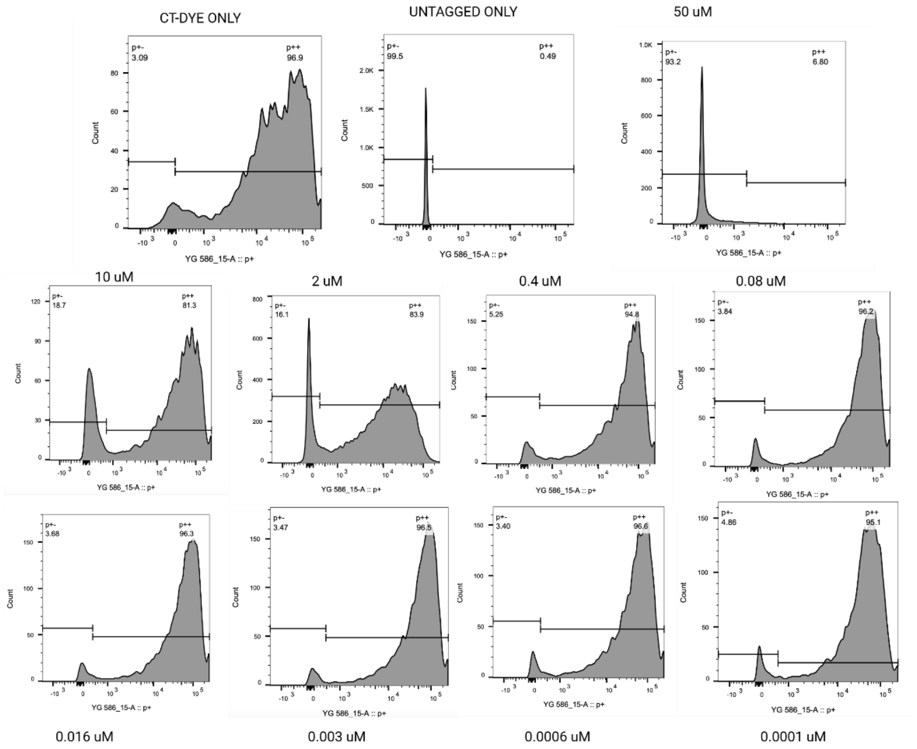

Flow Cytometry data for peptide

XVI. Late-stage functionalization of the MDA-modified protected arginine amino acid

In a 25 mL round-bottom flask equipped with a magnetic stir bar, N-butylpyrimidin-2-amine (**2**) (0.20 mmol, 1.0 equiv), 2-bromo-1-(4-chlorophenyl)ethan-1-one (**3b**) (0.22 mmol, 1.1 equiv), and DMAP (10 mol%) were added, followed by 5 mL of acetonitrile. The reaction mixture was stirred at room temperature for 10 h. Upon completion, the resulting white precipitate was collected by filtration and washed with acetone. The desired product (**4**) was isolated as a white solid in 90% yield and characterized by ^{1}H and ^{13}C NMR and confirmed by known literature.[4]

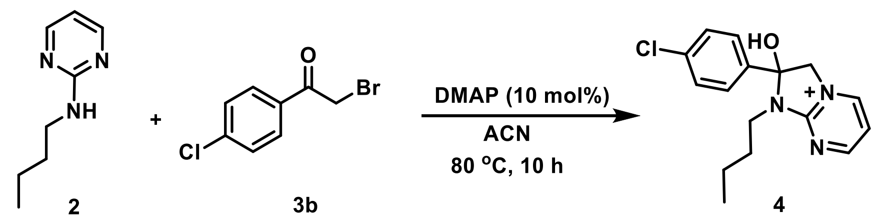

^{1}H NMR of compound 4 (400 MHz, DMSO): δ 9.04 (dd, J = 4.7, 2.0 Hz, 1H), 8.93 (dd, J = 6.3, 2.0 Hz, 1H), 7.94 (s, 1H), 7.80 (d, J = 8.7 Hz, 2H), 7.56 (d, J = 8.7 Hz, 2H), 7.33 (dd, J = 6.3, 4.7 Hz, 1H), 4.92 – 4.73 (m, 2H), 3.38 – 3.28 (m, 1H), 3.15 (ddd, J = 14.3, 9.4, 6.0 Hz, 1H), 1.50 – 1.27 (m, 2H), 1.15 (h, J =

7.4 Hz, 2H), 0.73 (t, J = 7.3 Hz, 3H). **^{13}C NMR of compound 4 (101 MHz, DMSO):** δ 167.6, 154.7, 148.2, 137.4, 134.1, 129.1, 128.4, 111.3, 90.4, 62.6, 40.6, 29.7, 19.3, 13.4.

^{1}H and ^{13}C NMR of compound 4 (400 MHz, DMSO)

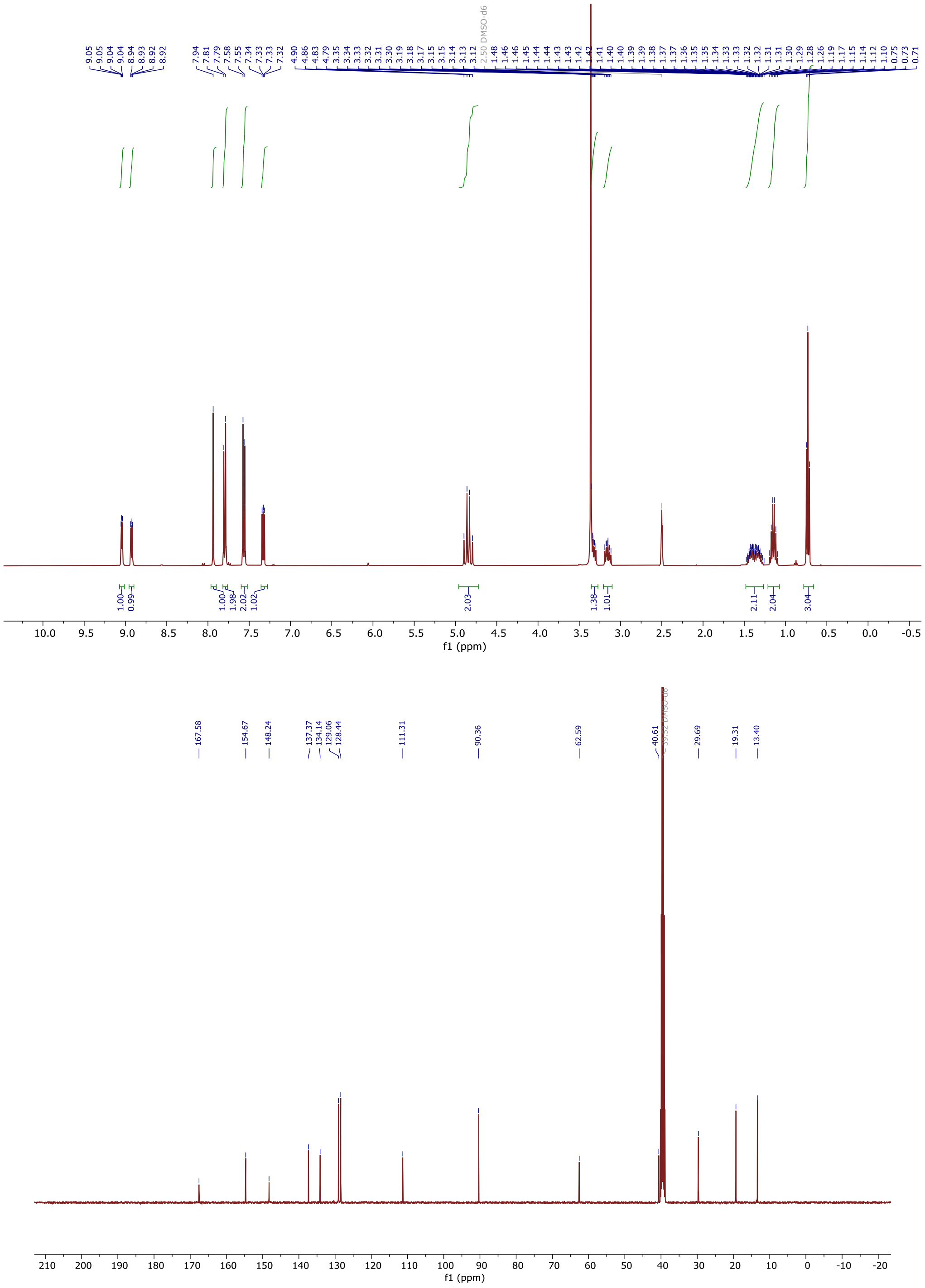

For the synthesis of compound 4a-4d: In a one-dram vial equipped with a magnetic stir bar, **2n** (0.019 mmol, 1.0 equiv), **3a-3d** (0.022 mmol, 1.1 equiv), and DMAP (10 mol%) were added, followed by 500 mL of acetonitrile. The reaction is allowed to stir in a oil-bath for 10 h. After 10 h, the reaction mixture was allowed to cool at room temperature. Subsequently, the reaction mixture was injected into the HPLC to determine the % conversion of **2n** to the labeled peptides **4a-4d** and their mass confirmed with LC-MS. HPLC analysis was carried out utilizing **HPLC Method A** at detection wavelength 220 nm. Refer to optimization table below for reaction conditions. The masses of the products were confirmed with LC-MS.

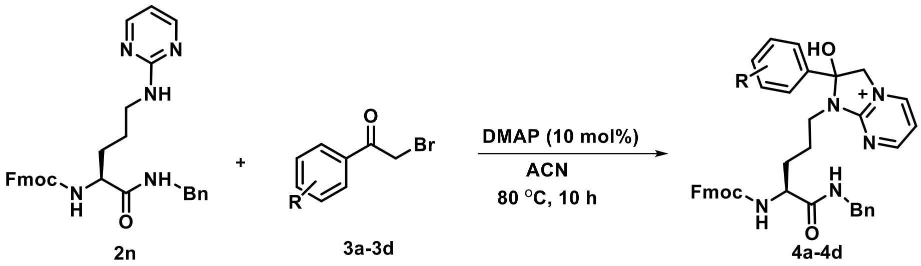

HPLC Trace of 2n at 220 nm

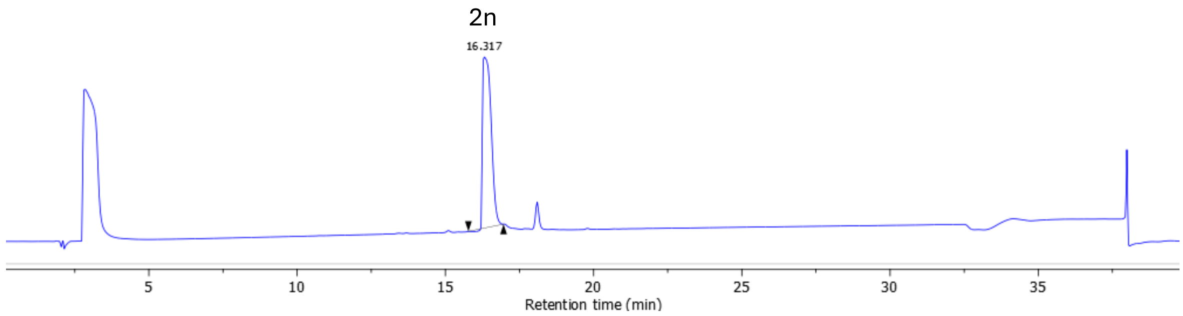

HRMS Trace of compound 2n

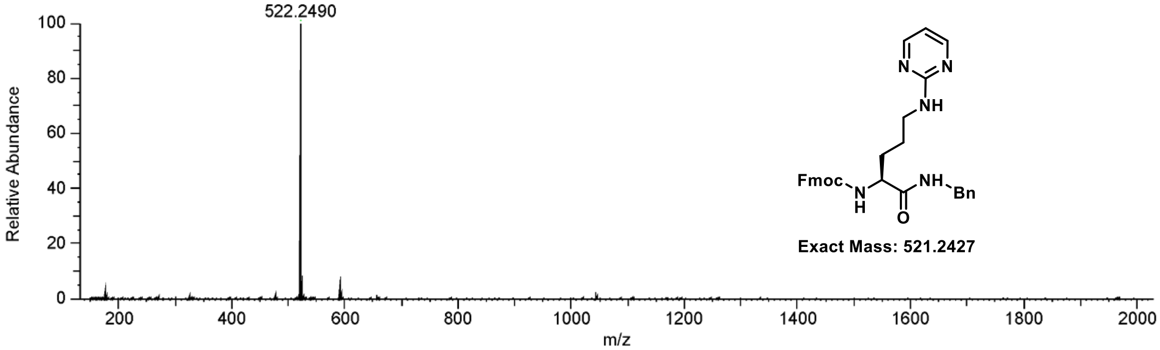

Compound 2n. LCMS for $C_{31}H_{32}N_5O_3$: *m/z* 522.2490 (calcd $[M+H]^+$ = 522.2500) (HPLC analysis at 220 nm). Retention time in HPLC: 16.3 min

HPLC Trace of 3a at 220 nm

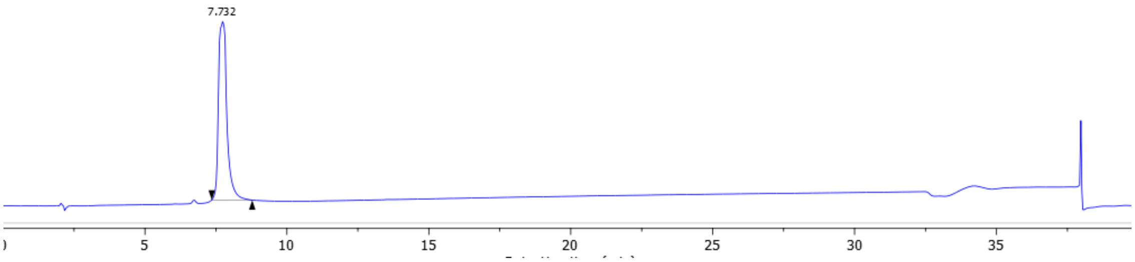

HPLC Trace of reaction mixture of 4a at 220 nm

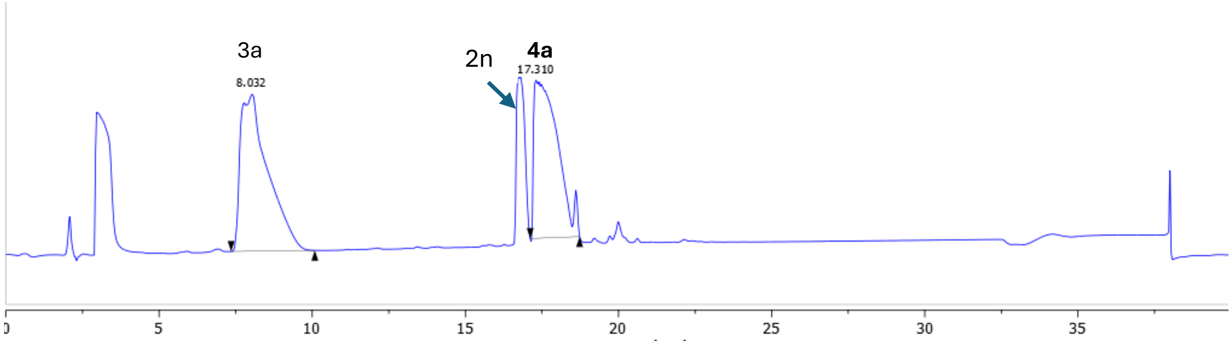

HRMS Trace of the peak at 17.3 min

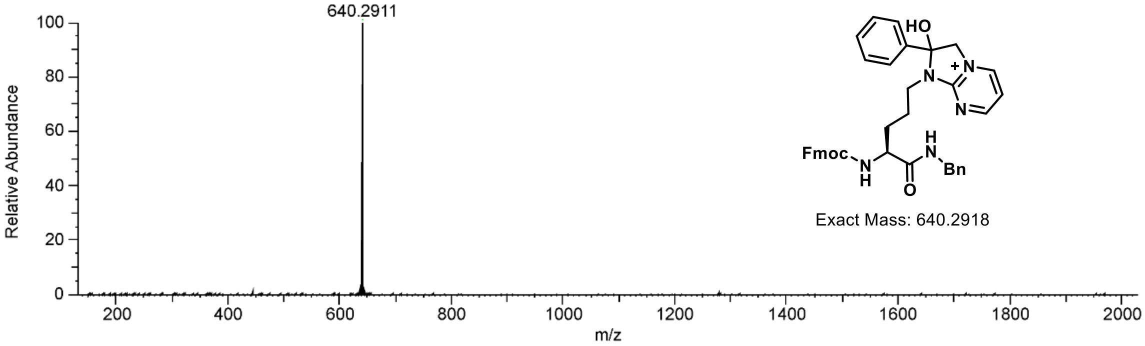

Compound 4a. LCMS for $C_{39}H_{38}N_5O_4$: *m/z* 640.2911 (calcd $[M]^+$ = 640.2918) (HPLC analysis at 220 nm). Retention time in HPLC: 17.3 min

HPLC Trace of 3b at 220 nm

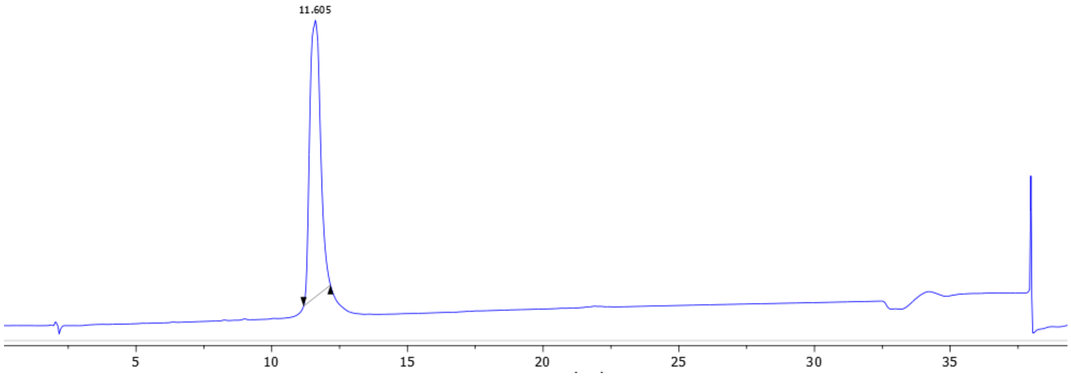

HPLC Trace of reaction mixture of 4b at 220 nm

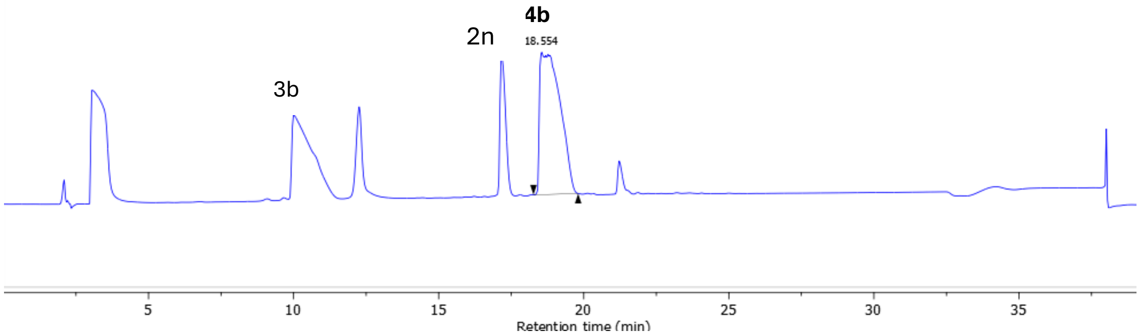

HRMS Trace of the peak at 18.5 min

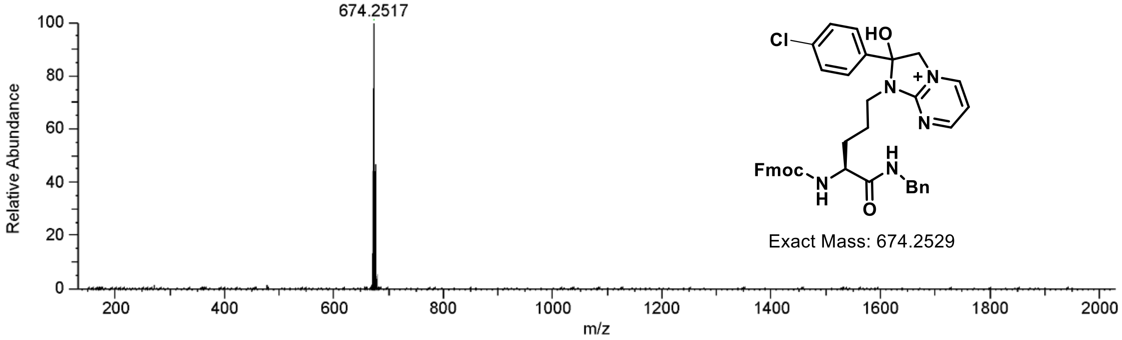

Compound 4b. LCMS for $C_{39}H_{37}N_5O_4Cl$: *m/z* 674.2517 (calcd $[M]^+$ = 674.2529) (HPLC analysis at 220 nm). Retention time in HPLC: 18.5 min

HPLC Trace of 3c at 220 nm

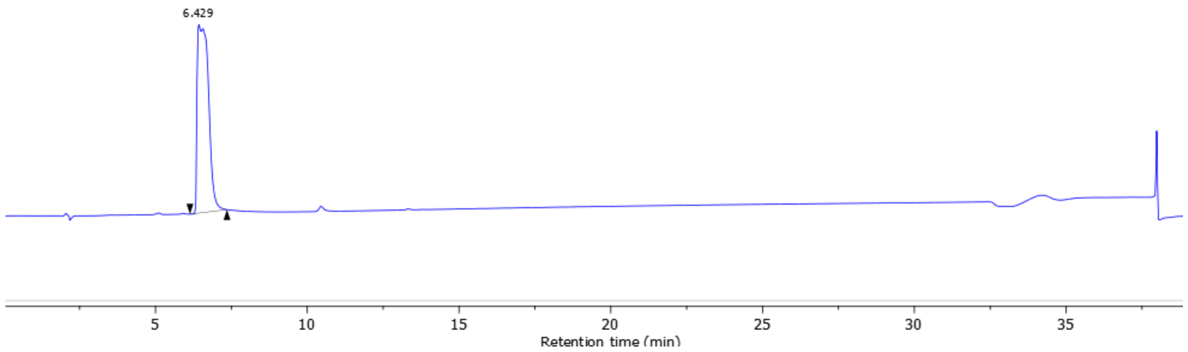

HPLC Trace of reaction mixture of 4c at 220 nm

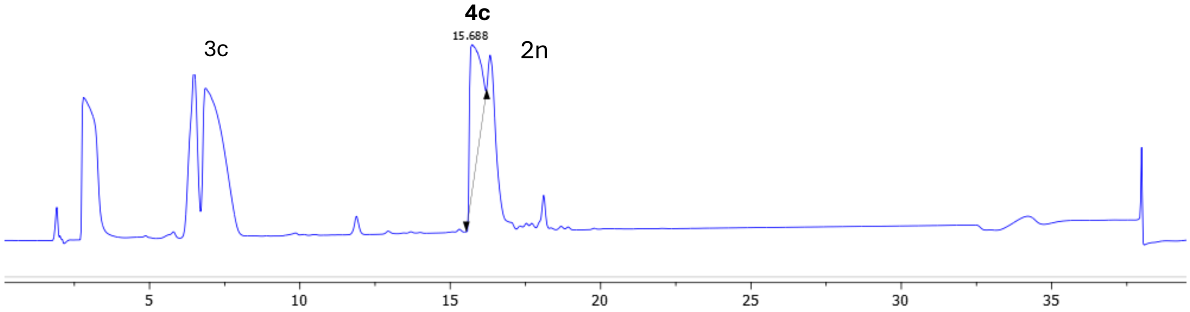

HRMS Trace of the peak at 15.6 min

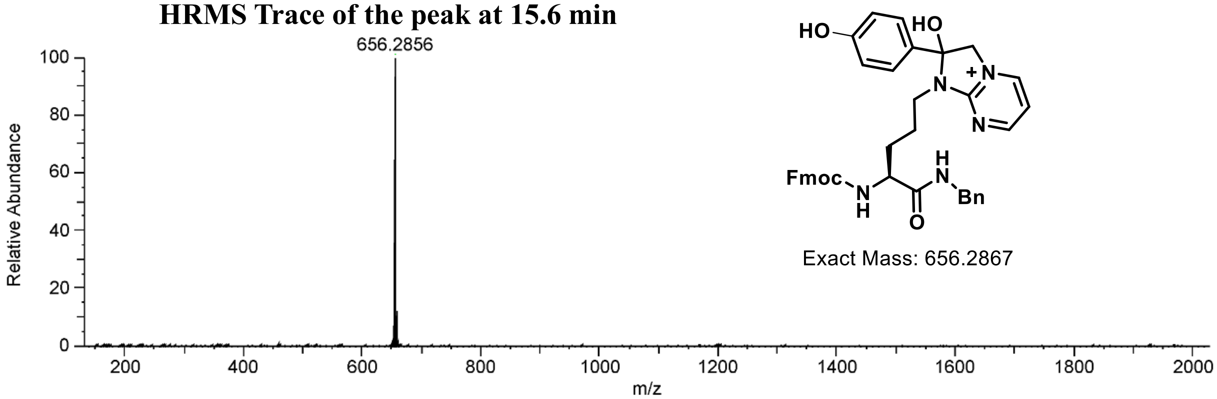

Exact Mass: 656.2867

Compound 4c. LCMS for $C_{39}H_{38}N_5O_5$: *m/z* 656.2856 (calcd $[M]^+$ = 656.2867) (HPLC analysis at 220 nm). Retention time in HPLC: 15.6 min

HPLC Trace of 3d at 220 nm

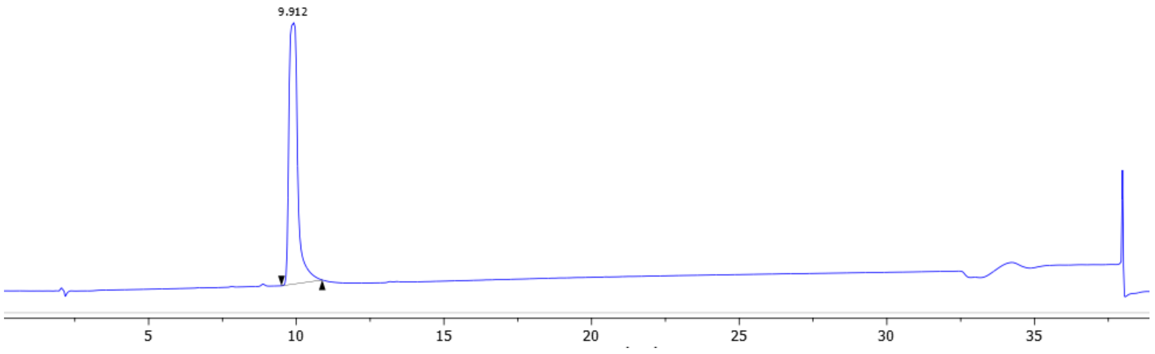

HPLC Trace of reaction mixture of 4d at 220 nm

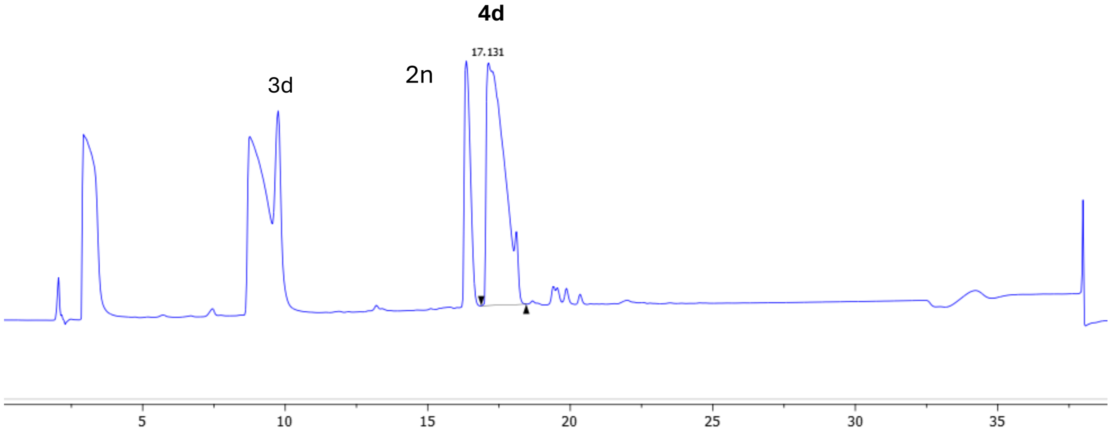

HRMS Trace of the peak at 17.1 min

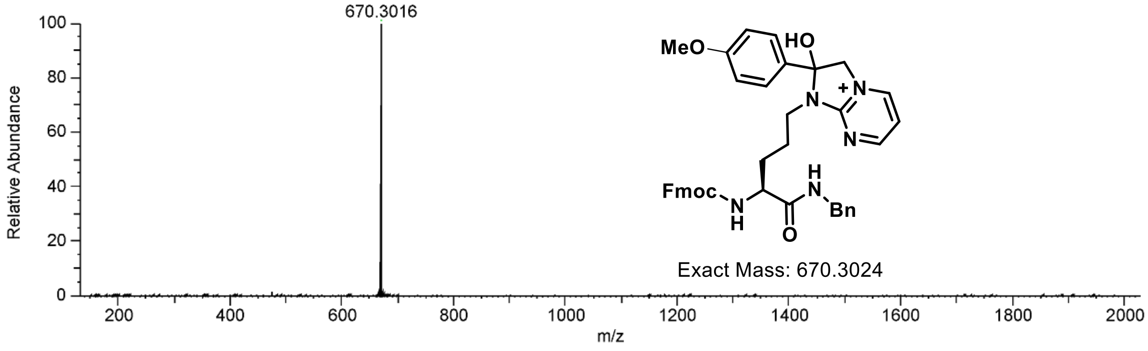

Compound 4d. LCMS for $C_{40}H_{40}N_5O_5$: *m/z* 670.3016 (calcd $[M]^+$ = 670.3024) (HPLC analysis at 220 nm). Retention time in HPLC: 17.3 min

XVII References:

1. Chan, W. C.; White, P. D. Fmoc solid phase peptide synthesis. *A practical approach (Oxford Univ. Press, New York)* **2000**.
2. Trivella, A.; Coussan, S.; Chiavassa, T. Malonaldehyde Synthesis. *Synthetic Communications*, **2008**, 3, 3285–3290.
3. (a) Steenackers, H. P. L.; Ermolat'ev, D. S.; Savaliya, B.; De Weerdt, A.; De Coster, D.; Shah, A.; Van der Eycken, E. V.; De Vos, D. E.; Vanderleyden, J.; De Keersmaecker, S. C. J. Structure‒activity relationship of 4(5)-aryl-2-amino-1H-imidazoles, N1-substituted 2-aminoimidazoles and imidazo[1,2-a]pyrimidinium salts as inhibitors of biofilm formation by Salmonella Typhimurium and Pseudomonas aeruginosa. *J. Med. Chem.* **2011**, *54*, 472-484. (b) Vandyshev, D. Y.; Mangusheva, D. A.; Shikhaliev, K. S.; Scherbakov, K. A.; Burov, O. N.; Zagrebaev, A. D.; Khmelevskaya, T. N.; Trenin, A. S.; Zubkov, F. I. Synthesis and Antimycotic Activity of New Derivatives of Imidazo[1,2-a]pyrimidines. *Beilstein J. Org. Chem.* **2024**, *20,* 2806-2817 and reference therein.
4. (a) Peraro, L.; Deprey, K. L.; Moser, M. K.; Zou, Z.; Ball, H. L.; Levine, B.; Kritzer, J. A. Cell penetration profiling using the chloroalkane penetration assay. *J. Am. Chem. Soc.* **2018**, *140*, 11360. (b) Deprey, K.; Kritzer, J. A. Quantitative measurement of cytosolic penetration using the chloroalkane penetration assay. *Methods Enzymol.* 2020, *641*, 277. (c) Klein, V. G.; Townsend, C. E.; Testa, A.; Zengerle, M.; Maniaci, C.; Hughes, S. J.; Chan, K.-H.; Ciulli, A.; Lokey, R. S. Understanding and improving the membrane permeability of VH032-Based PROTACs. *ACS Med. Chem. Lett*. **2020**, *11*, 9, 1732.
